# Predicting which colorectal cancer patients are most likely to improve their functional capacity with pre-surgery prehabilitation: a retrospective study based on the 6-min walk distance

**DOI:** 10.1007/s00520-026-11039-5

**Published:** 2026-07-27

**Authors:** M. de Klerk, M. J. W. van der Linden, A. P. M. Kerckhoffs, B. R. Meijboom, E. G. G. Verdaasdonk, E. de Vries

**Affiliations:** 1 Tranzo Scientific Center for Care and Wellbeing, Tilburg University, Tilburg, The Netherlands; 2 Jeroen Bosch Academy Research, Jeroen Bosch Hospital, ‘s-Hertogenbosch, The Netherlands; 3 Department of Dietetics, Jeroen Bosch Hospital, ‘s-Hertogenbosch, The Netherlands; 4 Department of Geriatrics, Jeroen Bosch Hospital, ‘s-Hertogenbosch, The Netherlands; 5 Department of Surgery, Jeroen Bosch Hospital, ‘s-Hertogenbosch, The Netherlands

**Keywords:** Colorectal cancer, Prehabilitation, 6-min walk distance, 6MWD, Predictive model, Functional capacity

## Abstract

**Purpose:**

Prehabilitation prior to colorectal cancer (CRC) surgery aims to improve functional capacity and postoperative outcomes, although results remain inconsistent due to variable programs. This study aimed to develop a predictive model for improvement in functional capacity during prehabilitation, using 6-min walk distance (6MWD) as the primary outcome. Identifying patients most likely to improve functional capacity during prehabilitation could support a more personalized use of prehabilitation.

**Methods:**

We conducted a retrospective observational study in CRC patients who underwent prehabilitation. Linear mixed-effects (lmer), linear (lm), generalized linear (glm) models, and machine learning algorithms (logistic regression, decision tree, bagged tree, boosted tree, random forest, and support vector machines) were compared to predict absolute and relative improvements and 14-, 20-, and 400-m pass in 6MWD outtake values. Model performance was assessed with ROC-AUC, confusion matrices, and Matthews correlation coefficient.

**Results:**

An lmer-based prediction model containing baseline 6MWD meters walked, sex, frailty, age, Charlson comorbidity index, and weight loss as fixed effects including interaction between frailty and age showed the best fit for predicting baseline and outtake 6MWD meters walked. A glm-based prediction model containing baseline 6MWD and age showed the best fit for predicting reaching the 400-m outtake threshold. Smaller improvements were not reliably predicted.

**Conclusion:**

Baseline and outtake 6MWD, as well as passing the 400-m threshold at outtake, can be reasonably estimated. Combining baseline 6MWD with the predicted probability of achieving the 400-m threshold identifies patients who are most likely to improve their functional capacity, thereby supporting personalized prehabilitation care.

**Supplementary Information:**

The online version contains supplementary material available at 10.1007/s00520-026-11039-5.

## Introduction

In recent decades, perioperative care for colorectal cancer (CRC) patients has been increasingly optimized, particularly through the implementation of the Enhanced Recovery After Surgery (ERAS®) guidelines. These guidelines aim to reduce perioperative stress, preserve postoperative physiologic function, and accelerate recovery [1]. Within this context, prehabilitation has emerged as a key component of preoperative care. Improved preoperative functional capacity is linked to fewer postoperative complications, shorter hospital stays, and lower readmission rates [2–4].

Prehabilitation programs ideally adopt a multimodal approach integrating physical training, nutritional optimization, mental support, and management of risk factors such as anemia, intoxications, and polypharmacy [5]. Additionally, identifying vulnerable patients is crucial to tailor interventions effectively and to ensure those at highest risk receive adequately adapted support. However, it is unclear whether vulnerable patients achieve the greatest improvement in functional capacity [6].

Functional capacity is a central target of exercise-based prehabilitation and is most commonly assessed using the 6-min walk distance (6MWD), a feasible and widely used field test in outpatient settings [7, 8]. Among reported benefits, improvement in aerobic fitness is the most consistently demonstrated outcome of physical exercise prehabilitation [9, 10]. Whether these improvements translate into better postoperative outcomes remains inconsistent across studies, partly due to heterogeneous protocols and populations but also because studies differ substantially in the types of outcomes assessed and the methods used to measure them [11, 12]. Patient characteristics such as baseline functional capacity, sex, nutritional status, and mental health further influence the magnitude of improvement [3, 13–15].

These patient-level differences in response, combined with growing pressure on healthcare systems, underscore the clinical and organizational need for a more personalized approach to prehabilitation. Not all patients need the same intensity or type of prehabilitation program; optimizing clinical and societal value depends on identifying those with the highest potential for improvement. As evidence for prehabilitation continues to grow, increasing attention is being directed toward understanding individual variation in functional response to prehabilitation. This study aims to develop a predictive model for improvement in functional capacity during prehabilitation in preoperative CRC patients, using the 6MWD as the primary outcome. By identifying patients most likely to improve their functional capacity as measured by the 6MWD, we aim to support a more personalized use of prehabilitation.

## Methods

### Study design

#### Setting

This retrospective single-center cohort study was performed at a large, nonacademic teaching hospital in the Netherlands (Jeroen Bosch Hospital [JBZ]). Prehabilitation was routinely offered preoperatively, but only patients who completed the program from October 2018 to September 2023 were included in this study. Data collected as part of usual care were retrieved from the JBZ electronic health records (EHRs) and records of the treating dietitians and physical therapists.

#### Participants

The study population included patients diagnosed with CRC who were scheduled for elective, curative-intent CRC resection, including those who had undergone chemoradiotherapy or radiotherapy. Although all patients scheduled for elective curative-intent CRC surgery were in principle eligible, participation in prehabilitation was voluntary. All participants underwent the prehabilitation program at one of the affiliated sites. Patients were excluded from this study when their functional capacity had not been evaluated or when they had declined the use of their medical data for research purposes.

#### Prehabilitation program

The multimodal prehabilitation program consisted of supervised exercise training, nutritional support, smoking and alcohol cessation counseling, anemia management, and medication optimization (in case of ≥ 5 medications/day) during the approximately 4-week period before surgery. Patients were referred by a member of the CRC team and participated in the program in either primary or secondary care. Before starting the program, patients underwent an individual assessment including the 6MWD, Steep Ramp Test (SRT), and one-repetition maximum (1RM) strength assessment to individualize the exercise program. The exercise program consisted of three supervised sessions per week, combining progressive resistance training and interval cycling, and four unsupervised home-based sessions per week of at least 1 h of walking or cycling. Nutritional counseling focused on achieving an adequate protein intake before surgery through personalized dietary advice. Further details of the prehabilitation program are provided in Supplementary Information 1.

#### Data extraction

The structured data were extracted pseudonymized from the EHRs of the JBZ (’s-Hertogenbosch, the Netherlands) using CTcue v4.14.2 (Amsterdam, the Netherlands) allowing full compliance with the General Data Protection Regulation. The query used is available in Supplementary Information 2. If necessary, unstructured or missing data were manually extracted from the EHR by researcher MK and verified by researchers ML and AK. These data were pseudonymized before further analysis was performed.

#### Primary outcome

The primary outcome of this study was improvement in functional capacity. Functional capacity can be assessed using various exercise tests, including the SRT and the 6-min walk test. We selected the 6MWD as the primary measure because the 6-min walk test is simple to administer and can be applied across a wide range of clinical settings. This makes it particularly suitable for assessment at the initial stage of the care pathway, when decisions regarding participation in prehabilitation may be made. The physical therapist assessed functional capacity at the beginning of the prehabilitation program (baseline) and at the end of the program (outtake). However, improvement in functional capacity based on the 6MWD lacks a standardized definition, with varying cutoff points used in the literature. After reviewing the literature (see Supplementary Information 3, including Table S1), multiple definitions of improvement based on the 6MWD were analyzed: (1) predicted sex- and age-adjusted distance [16], (2) absolute and relative change from baseline in meters, (3) ≥ 20-m improvement (clinically relevant threshold [17, 18]) [19, 20], (4) ≥ 14-m improvement (minimal clinically important difference) [21], and (5) outtake value of > 400 m (linked to morbidity and mortality) [22–25].

#### Variables

Other variables involved attended training sessions, location of the prehabilitation program, age, sex, body mass index (BMI), American Society of Anesthesiologists (ASA) score, intoxications, frailty (measured with the Clinical Frailty Scale [CFS] [26]), Charlson comorbidity index score [27], nutritional status (measured with Global Leadership Initiative on Malnutrition Statement [GLIM] criteria [28]), tumor-node-metastasis (TNM) status, oncologic data, SRT, postoperative complications (measured with the age-adjusted comprehensive complication index [29]), length of hospital stay, unplanned readmissions, and mortality 90 days after surgery.

### Study size

In this retrospective study, the sample size was determined by the available data. A post hoc power analysis was performed to quantify the power of the available sample to detect a clinically relevant within-patient change in 6MWD. To provide a conservative estimate, the smallest clinically relevant change examined in this study was used, corresponding to the minimal clinically important difference of 14 m reported in previous literature [21]. Based on this 14-m difference and the observed standard deviation (SD) of the individual change scores of 52.3 m, the standardized effect size for a paired comparison was 0.268 (Cohen’s *d*_*z*_). With 232 patients with complete baseline and outtake 6MWD measurements, a two-sided alpha of 0.05, and a paired-samples *t* test, the achieved power was > 90%.

### Descriptive analysis

Descriptive statistics were performed in IBM SPSS Statistics for Windows, Version 27. Normality was assessed with Kolmogorov–Smirnov and Shapiro–Wilk tests. Normally distributed variables were summarized as means and SDs, nonnormal variables as median and interquartile ranges (IQRs). Categorical variables were presented as frequencies and percentages per category. Data were visualized with histograms, scatterplots, and boxplots. Before building models, the independent numerical variables were transformed to *z*-scores (scaling). Correlations between independent variables were examined using Pearson’s correlation coefficient when distributions were approximately normal and Spearman’s rank correlation coefficient otherwise. Correlation strength was classified as mild (0.20–0.39), moderate (0.40–0.59), or strong (≥ 0.60).

The primary outcome variable (6MWD, baseline and outtake) was analyzed as a numeric (e.g., meters walked) or a categorical (e.g., norm for age and sex reached yes/no) variable as needed, along the line of the cutoffs identified in the literature (see above).

### Statistical analysis

Statistical analyses were conducted in R version 4.3 using the lme4, tidyverse, tidymodels, sjPlot, performance, rpart, xgboost, ranger, baguette, and ModelMetrics packages.

In the first statistical analysis (analysis A, details in Supplementary Information 4), models with the meters walked as longitudinal numerical outcome variable (whether measured before or after prehabilitation) were built using linear mixed-effects regression (lmer function) with the patient as the grouping variable. Model comparisons were based on the Akaike information criterion (AIC) using ANOVA (analysis of variance), with the lowest AIC indicating the best model. Independent variables were selected based on previous literature demonstrating their association with functional capacity or response to prehabilitation, combined with the domain expertise in the CRC team and their (inherent) availability before initiation of the prehabilitation program. We included comorbidities (measured with Charlson comorbidity index), CFS, age, sex, ASA score, hemoglobin level, intoxications (alcohol and drug use), tumor location, number of tumors, neoadjuvant therapies, location of prehabilitation program, and GLIM-defined malnutrition including BMI and weight loss [15, 30–32] (definitions in Table 1). Although adherence to the exercise program is likely to influence improvement in 6MWD, it cannot be incorporated into a prediction model intended for pre-prehabilitation decision-making because attendance is (inherently) unknown at baseline.

**Table 1:** Definitions of the independent variables used in the statistical analyses

Variable	Measurement time point	Definition
Charlson comorbidity index	Preoperative screening	Numeric composite score of comorbidities
Clinical frailty score	Preoperative screening	Categorized as nonfrail (CFS ≤ 3) and frail (CFS ≥ 4)
Age	Date of surgery	Numeric variable (years)
Sex	Preoperative screening	Categorized as male/female
ASA score	Preoperative screening	Categorized as ASA I-II/ASA III-IV
Hemoglobin level	First hospital appointment	Numeric variable categorized as anemia at baseline/no anemia at baseline (anemia defined as Hb < 6.8 g/L)
Hemoglobin level	Date of surgery	
Intoxications	First appointment dietitian	*Smoking:* categorized as non- or former smoker/active smoker *Alcohol use:* categorized as no or former/active
Intoxications	Last appointment dietitian	
Tumor location	Date of surgery	Categorized as colon/rectum
Number of tumors	Date of surgery	Numeric variable
Neoadjuvant therapy	Date of surgery	Categorized as chemoradiation/chemotherapy/radiotherapy
Location of prehabilitation program	First appointment dietitian or physiotherapist	Categorized as primary care/secondary care
GLIM	First appointment dietitian	Categorized as well-nourished/malnourished
BMI	First appointment dietitian	Numeric variable in kg/m^2^
Weight loss	First appointment dietitian	Numeric variable; percentage reduction in body weight compared to usual body weight

*ASA* American Society of Anesthesiologists, *BMI* body mass index, *CFS* clinical frailty score, *GLIM* Global Leadership Initiative on Malnutrition score, *Hb* hemoglobin, *L* liter, (*k)g *(kilo)gram, *m* meter

Independent variables were tested separately and in combinations as fixed effects, including two-way interaction. Final model performance was visualized with the check_model function and the marginal *R*^2^ (variance explained by the fixed effects), conditional *R*^2^ (variance explained by both fixed and random effects), and the root mean square error (RMSE, evaluates the average prediction error).

In the second analysis (analysis B, details in Supplementary Information 4), linear regression (lm function) was used to model the absolute and relative increases in walked meters following the same steps as in analysis A, using a *p* value threshold of 0.05 and inspection of residuals (lower).

In the third statistical analysis (analysis C, details in Supplementary Information 4), logistic regression (glm function) was used for categorical outcomes including at least 14 or 20 m increase (outtake minus baseline value), reaching 400 m (outtake value), and reaching the predicted normative value (sex-, age-, height-, and weight-adjusted [33]; outtake value). The same steps as in analyses A and B were followed, with the exception that both the *p* value (< 0.05) and the AIC (lowest) were used for model selection; the coefficient of discrimination (Tjur’s *R*^2^; 0 = no discrimination, 1 = perfect discrimination) was used for showing the variance explained.

### Machine learning

Machine learning models were built with the tidymodels package using all relevant variables and those included in the final models from analyses A to C (see above). For each model, the dataset was split into training (75%) and test (25%) sets using stratification based on the outcome variable. As algorithms incorporated in the machine learning models, logistic regression (glm), decision tree (rpart), bagged tree (rpart), boosted tree (xgboost), random forest (ranger), and support vector machines (kernlab) were used with automated tuning where applicable. Model evaluation was performed using resampling in the training dataset and validation in the test dataset, with the area under the receiver operating characteristic curve (ROC-AUC), confusion matrices (false/true positives and false/true negatives), and the Matthews correlation coefficient (MCC) as evaluation metrics.

## Results

### Participants

**Fig. 1:**
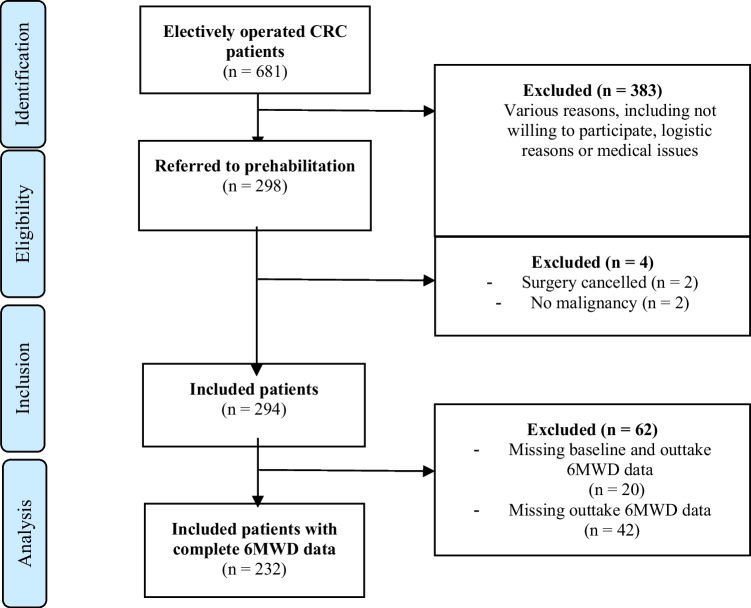
Flow diagram selection and inclusion process

From October 2018 up to September 2023 (including), 681 patients underwent elective surgery for colorectal cancer. A total of 298 patients were referred to the prehabilitation program and were eligible, and 232 patients were included in this study. Figure 1 shows the flow diagram of the selection and inclusion process.

### Descriptive analysis

We excluded 62 otherwise eligible patients due to the absence of 6MWD outtake data. Their mean baseline 6MWD was 410 m (SD 114; range 130–615) as compared to 422 m (SD 110; range 90–692) in the *included* patients (*p* = 0.513 using t-test). Various factors contributed to the absence of the 6MWD data, including logistical issues, illness, hospital admissions, physical limitations/injuries, and being on holiday.

The characteristics of the 232 *included* patients are shown in Table 2. The median age was 74 years (IQR 11; range 54–88) and 44% were female. The prevalence of comorbidities was reflected in a median Charlson comorbidity index of 6 (IQR 2; range 3–13), with 13% of the patients classified as frail. Patients attended a median of 12 supervised prehabilitation training sessions (IQR 4.75; range 2–23), with 82% participating in the prehabilitation program in the secondary care (hospital) setting.

**Table 2:** Baseline characteristics of the included patients

Characteristic	Included patients *n* = 232
	Value
*Demographic data*	
Sex (female), *n* (%)	102 (44)
Age (years), median (IQR; min–max)	74 (11; 54–88)
Charlson comorbidity index^a^ Median (IQR; min–max) Score 3–5, *n* (%) Score 6–8, *n* (%) Score 9–11, *n* (%) Score ≥ 12, *n* (%)	6 (2; 3–13) 119 (40) 140 (48) 32 (11) 3 (1)
Clinical Frailty Scale, *n* (%) Nonfrail (score 0–3) Frail (score ≥ 4)	202 (87) 30 (13)
Current smoker, *n* (%)	21 (9)
Current alcohol consumer, *n* (%) < 2 units/day 2–6 units/day > 6 units/day	111 (48) 89 (38) 22 (10) 0
BMI (kg/m^2^), median (IQR; min–max)	26.4 (6; 18–53)
Nutritional status^b^, *n* (%) Well-nourished Moderately malnourished Severely malnourished	(*n* = 225) 172 (74) 42 (18) 11 (5)
Weight loss (percentage), median (IQR; min–max)	(*n* = 228) −1 (−5; −18–+ 13)
Protein intake relative to need (%), median (IQR; min–max)	80 (26; 35–194) (*n* = 225)
*Functional capacity and prehabilitation data*	
Baseline 6MWD (m), mean (SD; min–max)	422 (110; 90–692)
Baseline SRT (watt), median (IQR; min–max)	160 (86.5; 37.5–583)
Supervised prehabilitation training sessions, median (IQR; min–max)	12 (4.75; 2–23)
Prehabilitation site, *n* (%) Primary care Secondary care	41 (18) 191 (82)
*Oncologic data*	
Baseline hemoglobin (g/L), median (IQR; min–max) Preoperative anemia, *n* (%) No anemia at baseline Anemia at baseline After correction^c^ Hb ≥ 6.8 After correction Hb < 6.8 No correction	7.7 (2; 4.6–10.7) 172 (74) 60 (26) 36 (16) 16 (7) 8 (3)
Tumor location, *n* (%) Colon Rectum	181 (78) 51 (22)
TNM classification^d^, *n* (%) I II III IV	70 (30) 75 (32) 78 (34) 4 (2)
Neoadjuvant therapy^e^, *n* (%) Chemoradiation Radiotherapy Chemotherapy	14 (6) 7 (3) 4 (2)
*Surgical data*	
ASA index, *n* (%) I–II III IV	129 (56) 93 (40) 10 (4)
Surgical technique, *n* (%) Laparoscopic Open	216 (93) 16 (7)
Number of patients with postoperative complications, *n* (%) Surgical complications Medical complications	75 (32.3) 61 (26.3) 35 (15.1)
Comprehensive complication index^f^, median (IQR; min–max)	11.8 (20.9; 0–100)
Surgical reintervention, *n* (%)	31 (13.4)
Intensive care admission, *n* (%)	9 (3.9)
Length of hospital stay (days), median (IQR; min–max) Tumor location colon Tumor location rectum Prolonged length of hospital stay^†^, *n* (%)	4 (3; 2–52) 4 (3; 2–52) 4 (3; 3–37) 19 (8.2)
Unplanned hospital readmission^g^, *n* (%)	22 (9.5)
Mortality^7^, *n* (%)	4 (1.7)

*6MWD* 6-min walk distance, *ASA* American Society of Anesthesiologists score, *BMI* body mass index, *IQR* interquartile range, *min* minimum, *max* maximum, *m* meter, *SD* standard deviation, *SRT* Steep Ramp Test, *watt* wattage, *TNM* tumor node metastasis

^a^Based on Glasheen et al. [27], age adjusted

^b^Based on Global Leadership Initiative on Malnutrition (GLIM) criteria [28]

^c^Correction with intravenous ferric carboxymaltose transfusion or blood transfusion

^d^Based on Union for International Cancer Control 8th edition for colorectal cancer

^e^Adjuvant therapies only apply to patients with rectal cancer or (in beginning) irresectable T4 colon tumors

^f^Based on Slankamenac et al. [29]

^g^Measured 90 days after surgery

^†^Prolonged length of hospital stay is defined as > 14 hospital days of stay

As previously mentioned, the literature identifies multiple cutoff points for (clinically relevant) improvement in the 6MWD; therefore, we present the patients’ 6MWD data related to these various cutoff points in Table 3.

**Table 3:** Six-minute walk distance results according to the various cutoff points described in the literature

Variable	Included patients *n* = 232
	Outcome
Baseline 6MWD (m), mean (SD; min–max)	422 (110; 90–692)
Outtake 6MWD (m), mean (SD; min–max)	468 (109; 180–717)
Difference between baseline 6MWD and outtake 6MWD (m), median (IQR; min–max)	33.5 (48.75; −75–300)
Relative improvement between baseline and outtake 6MWD (x), median (IQR; min–max)	1.08 (0.13; 0.71–2.67)
Improvement 6MWD, *n* (%) > 14 m > 20 m	180 (78) 157 (68)
6MWD > 400 m, *n* (%) Baseline Outtake	138 (60) 168 (72)
6MWD equal to or higher than predicted value^a^, *n* (%) Baseline Outtake	76 (33) 118 (51)

*6MWD* six-minute walk distance, *m* meter, *max* maximum, *min* minimum, *IQR* interquartile range, *SD* standard deviation, *x* multiplication factor

^a^Based on Enright and Sherill; sex- age-, height-, and weight-adjusted [33]

### Statistical analysis (for details, see supplementary 4 and 5)

#### Analysis A: linear mixed effects regression

**Fig. 2:**
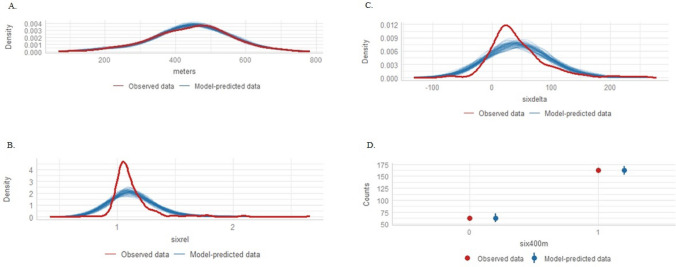
Posterior predictive check of the model **A** fit, **B** absolute improvement in 6MWD (variable name “sixdelta” in figure) fit, **C** relative improvement in 6MWD (variable name “sixrel” in figure) fit, and **D** 6MWD > 400 m at outtake (variable name “six400m” in the figure) fit. For models **A**, **B**, and **C**, good fit model is indicated when the model-predicted lines closely resemble the observed data line, which is observed for model **A**. For model **D**, good model fit is indicated when the model predicted intervals include the observed data points without overlap, which is also the case in this panel

**Fig. 3:**
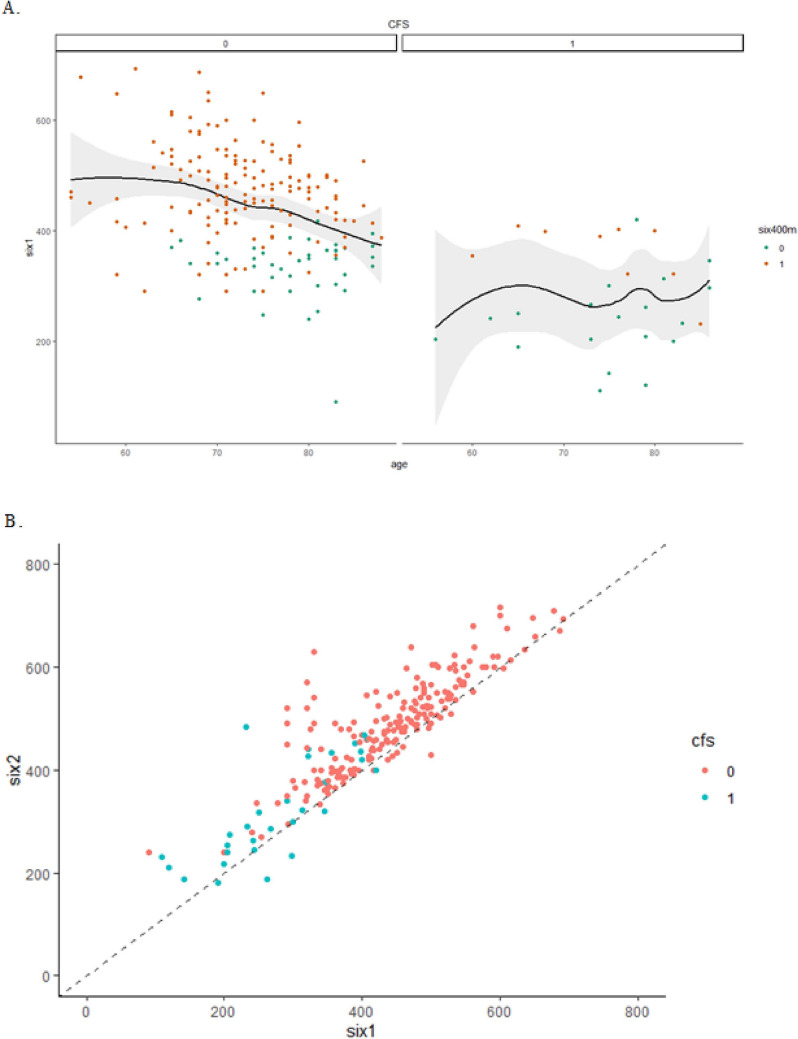
**A** The relationship between age, meters walked at baseline (six1 in the figure), CFS (0 = nonfrail on the left, 1 = frail on the right), and 6MWD > 400 m at outtake (six400 in the figure; 0 = false, 1 = true), as modeled using the loss function. **B** The relationship between meters walked at baseline (six1 in figure) and meters walked at outtake (six2 in figure) and CFS (0 = nonfrail, 1 = frail). The figure includes the 45 degree (dashed) line “*x* equals *y*.” Points above this line indicate better performance at outtake than at intake

Model A focused on predicting the meters walked using the 6MWD (baseline and outtake data). In the course of analysis A, the following variables were dropped: smoking, number of tumors, neoadjuvant therapy, number of training sessions, GLIM, ASA score, alcohol consumption, tumor location, location of prehabilitation program, BMI, and hemoglobin level. Only the two-way interaction between the variables CFS and age had a lower AIC than the model without and was included in further analysis. The final model therefore was meterswalked ~ 6MWDtimepoint + sex + (CFS * age) + Charlson comorbidity index + weight loss + (1|pseudoid)). Visual demonstration using the check_model function showed a reasonably good fit (Fig. 2A). The final model explained 58% of the fixed effects (marginal *R*^2^) and 94% of the combined fixed and random effects (conditional *R*^2^); the root mean squared error (RMSE) was 18.8 m. The relationship between age, meters walked at baseline, CFS, and 400 m walked at outtake achieved is shown in Fig. 3A; the relationship between meters walked at baseline and outtake and CFS is shown in Fig. 3B.

#### Analysis B: linear regression models

Model B-absolute focused on the absolute increase in meters walked (outtake minus baseline) during the 6MWD. In the course of analysis B-absolute, all variables except baseline 6MWD, location of prehabilitation program, and weight loss were dropped. Visual demonstration using the check_model function showed poor fit of the final model as shown in Fig. 2B.

Model B-relative focused on the relative increase in meters walked during the 6MWD. In the course of analysis B-relative, all variables except baseline 6MWD, location of prehabilitation program, and weight loss were dropped. Including a three-way interaction between the remaining independent variables showed significantly lower residuals [lm(relative improvement in 6MWD ~ baseline 6MWD*location of prehabilitation program*weight loss)]. However, visual demonstration using the check_model function showed poor fit of the final model as shown in Fig. 2C.

#### Analysis C: logistic regression models

Models C focused on the categorical outcome variables (see “Methods”) and used the same steps as in analysis B. Model C-14 m (≥ 14-m increase in 6MWD) and model C-20 m (≥ 20-m increase in 6MWD) did not show a good fit (Tjur’s *R*^2^ 0.064 and 0.060 respectively). Model C-pred (achieved predicted normative 6MWD at outtake) did show a reasonable fit (Tjur’s *R*^2^ = 0.471), but the model predicted data overlapped substantially between patients who achieved the predicted normative 6MWD and those who did not (for the posterior predictive check visual, see Supplementary Information 5, Figure S1). Model C-400 m (6MWD > 400 m at outtake) did show a good fit of the final model (glm(6MWD > 400 m at outtake ~ baseline 6MWD + age)); see Fig. 2D (posterior predictive check), with a Tjur’s *R*^2^ of 0.592.

### Machine learning (for details, see supplementary information 4)

The machine learning models compared predictive performance using logistic regression, decision tree, bagged tree, boosted tree, random forest, and support vector machine algorithms, respectively, both with all independent variables included in analyses A, B, and C in one set of models (except for boosted tree which does not accept categorical variables), as well as only the independent variables being part of the final models A, B, and C using the same outcome variable (14 m, 20 m, and 400 m, see above) in another set of models in combination with the various outcomes as described. For predicting at least 14 or 20 m improvement at outtake, none of the tested algorithms produced a suitable predictive model (MCCs < 0.2; ROC-AUCs 0.4–0.6). In contrast, for predicting whether patients reached 400 m on the 6MWD at outtake, reasonably good model fit could be obtained when using only the independent variables being part of the final models A, B, and C with all the used algorithms except support vector machines which consistently only predicted positives. Logistic regression and boosted tree algorithms showed the best visual fit; the latter also showed the best MCC (but is computationally more intensive and less straightforward to explain). See Supplementary Information 5 (Table S2 and Figure S2A-E).

### Wrap up for potential clinical application

The results of the analyses above show that it could be possible to obtain a reasonably good estimate of achieving more than 400 m walked at outtake 6MWD with a simple procedure including a 6MWD during the first outpatient visit (e.g., in the clinic’s corridor, performed by support personnel) and adding the result in meters and the patient’s age in years into the glm formula (see above; e.g., in the healthcare professional’s computer or in an app). A more complicated approach would include obtaining values for sex and age (easy) and weight loss, CFS, and Charlson comorbidity index (increasingly labor-intensive). These values can then be entered into the lmer model to generate a predicted baseline as well as outtake 6MWD value (or use the baseline value as input for the glm formula to estimate whether 400 m walked can be reached at outtake).

## Discussion

This study aimed to develop a model to predict improvement in functional capacity during prehabilitation in preoperative CRC patients, using the 6MWD as the primary outcome. Our findings show that linear mixed-effects regression provided a reasonably good estimate of walked meters in the 6MWD at baseline and at outtake, based on baseline performance, sex, CFS and age in interaction, the Charlson comorbidity index, and weight loss (see “Results”). Logistic regression further demonstrated that baseline walked meters in the 6MWD and age predicted reasonably good whether patients reached the clinically meaningful threshold of 400 m at outtake (see “Results”). This threshold offers a practical clinical application, as the model may help identify patients who are (un)likely to reach this threshold following prehabilitation. Notably, improvements were observed across almost all patients, including those who started with higher baseline performance, although smaller improvements (14 or 20 m) could not be predicted reliably.

Previous research identified baseline functional capacity, comorbidity burden, and nutritional status as key determinants of physical performance [34, 35]. Van Vught et al. [36] showed that malnutrition and sarcopenia are associated with reduced physiological reserve and poorer recovery outcomes. Our findings align with these observations, as patients with greater weight loss showed poorer physical performance. Furthermore, our study adds nuance by explicitly examining how frailty (CFS) modifies the relationship between age and functional performance. Although previous studies often focus on chronological age as a predictor of poorer physical performance, as shown in Fig. 3A, our study shows that chronological age primarily influences the meters walked in the 6MWD in *non*frail patients (with a wide spread). However, among frail patients, performance is largely constrained by frailty itself, with age adding no explanatory value. This pattern shows that chronological age alone is an insufficient indicator of recovery potential. Instead, frailty and chronological age together provide a more accurate reflection of a patient’s biological resilience and capacity for improvement. This finding underscores the need to shift beyond age-based stratification in clinical practice.

Clinically, our findings suggest two potential approaches through which prediction models may support personalized prehabilitation. The first approach is predicting the absolute distance a patient is likely to walk before and after prehabilitation. This may help identify patients who already achieve sufficient functional performance and may be suitable for adapted, home-based or hybrid programs, while prioritizing those with a predicted limited improvement for more personalized prehabilitation. The second approach involves predicting whether a patient will exceed the clinically meaningful 400-m threshold at outtake, a marker of functional independence and postoperative resilience [14]. This can be estimated using a regression model using only age and baseline 6MWD, making it practical for clinical use. Having patients complete a baseline 6MWD and estimating their expected outtake value could provide a practical and resource-efficient basis for treatment planning, particularly in settings with limited resources. Although the 400-m threshold is not an established standard, it may serve as a meaningful benchmark for clinical decision-making. Using the 6MWD as outcome measure also improves feasibility in both hospital and primary care settings compared with the more accurate but less practical cardiopulmonary exercise test (CPET). However, before widespread implementation, this approach requires further validation in independent CRC cohorts.

These approaches illustrate the potential of predictive modeling to support the future personalization of prehabilitation according to functional capacity. Although functional capacity is an important treatment target, it represents only one dimension of the potential benefits of multimodal prehabilitation. Patients may also experience improvements in muscle strength, nutritional status, psychological well-being, quality of life, and postoperative recovery [37, 38]. Therefore, these models should be interpreted as potential tools to predict changes in functional capacity rather than the overall benefits of multimodal prehabilitation. The tools are not intended to determine which patients should or should not receive prehabilitation but to support recognition of patients who may need different or longer prehabilitation programs to which they may still respond. Future research is needed to elaborate this further.

The present findings should be interpreted in light of the limitations of this study. Attrition due to COVID 19-related program discontinuation led to missing follow-up data which may have introduced bias. Selection bias is also possible, as more motivated patients may be overrepresented. In addition, detailed information on exercise dose, including achieved training intensity, workload, and duration, was not consistently available for all patients, limiting the inclusion of actual training exposure to the number of training sessions attended. Finally, randomization to a no-prehabilitation control group was considered inappropriate given growing evidence for the benefits of prehabilitation.

Strengths of the study include the consistent application of standardized perioperative care based on the national Dutch guideline across all patients which enhances generalizability, a relatively large and homogeneous CRC cohort, and the comprehensive use of all program components, including nutritional support and supervised high-intensity training. The broad inclusion of patients including those of varying age, comorbidity, and baseline functional capacity supports generalizability.

## Conclusion

It was possible to develop a reasonably well-fitting prediction model of improvement in the 6-min walk test after prehabilitation in a retrospective cohort of high-risk surgical CRC patients. Baseline and outtake 6MWD, as well as reaching the 400-m threshold at outtake, could be estimated with a reasonably good model fit. A model combining baseline 6MWD and age in particular showed potential for use in outpatient hospital settings to estimate whether patients will exceed 400 m at outtake. Follow-up studies should focus on contributing to more personalized prehabilitation and resource allocation in preoperative CRC care.

## Supplementary Information

Below is the link to the electronic supplementary material.

ESM 1 (DOCX 17.8 KB)

ESM 2 (DOCX 67.8 KB)

ESM 3 (DOCX 26.5 KB)

ESM 4 (DOCX 24.5 KB)

ESM 5 (DOCX 435 KB)

## Data Availability

Data supporting the findings of this study are available from the corresponding author upon reasonable request.
