# Supplementary material for: Predicting which colorectal cancer patients are most likely to improve their functional capacity with pre-surgery prehabilitation: a retrospective study based on the 6-min walk distance
Source: Support Care Cancer. 2026 Jul 27;34(8):805. doi: 10.1007/s00520-026-11039-5 (PMC13407944; doi:10.1007/s00520-026-11039-5)
Supplement: Supplementary file 1 — (DOCX 17.8 KB) [file 520_2026_11039_MOESM1_ESM.docx]

Predicting which colorectal cancer patients are most likely to improve their functional capacity with pre-surgery prehabilitation: A retrospective study based on the six-minute walk distance. Supportive Care in Cancer. M. de Klerk, M.J.W. van der Linden, A.P.M. Kerckhoffs, B.R. Meijboom, E.G.G. Verdaasdonk, E. de Vries. Tranzo Scientific Centre for Care and Wellbeing, Tilburg School of Social and Behavioral Sciences, Tilburg University, Warandelaan 2 5037 AB Tilburg, The Netherlands, m.deklerk@tilburguniversity.edu

**Supplementary Information 1. Detailed description of the multimodal prehabilitation program**

**Program setting**

The multimodal prehabilitation program was delivered during the approximately four-week period preceding elective colorectal cancer surgery. Patients were referred by a member of the colorectal cancer treatment team and participated either at the hospital outpatient physiotherapy department or at an affiliated primary care physiotherapy practice. Hospital supervised exercise sessions were delivered by two physiotherapists in groups of approximately six patients (maximum ten participants). In primary care, supervised exercise sessions were delivered by one or two physiotherapists and were organized in group settings whenever possible. The maximum group size varied between physiotherapy practices.

**Baseline assessment**

Before entering the program, each patient underwent an individual 45-minute to one hour physiotherapy assessment including medical history, risk assessment, and physical performance testing. Functional capacity was assessed using the six-minute walk test (6MWT), Steep Ramp Test (SRT), and a one-repetition maximum (1RM) strength assessment. Based on the results of the baseline assessment, an individualized exercise program was developed for each patient. All assessments were repeated after completion of the program to evaluate changes in physical performance.

**Exercise program according to the FITT-VP principles[1]**

| **Principle** | **Description** |
| --- | --- |
| **Frequency** | Three supervised exercise sessions per week and home-based sessions on non-supervised days. |
| **Intensity** | Resistance training was individualized based on the baseline 1RM assessment. Patients started at approximately 70% of the estimated 1RM during week one and progressed to approximately 80% of the estimated 1RM during weeks two to four, depending on individual tolerance. Aerobic interval training intensity was based on the Maximum Short Exercise Capacity (MSEC) and Borg Rating of Perceived Exertion (low intensity: approximately 18% MSEC or Borg score 11; high intensity: approximately 36% MSEC or Borg score 15). |
| **Time** | Supervised sessions lasted approximately one hour. Aerobic interval training consisted of three 7-minute cycling blocks, each comprising 3 minutes of low-intensity cycling followed by 4 minutes of high-intensity cycling. Home-based exercise consisted of at least one hour of walking, cycling, or a combination of both on non-supervised training days. |
| **Type** | Supervised exercise combined progressive resistance training and interval cycling on a cycle ergometer. Home-based exercise consisted of low-intensity walking or cycling. |
| **Volume** | Approximately seven exercise sessions per week (three supervised and four home-based). Resistance training consisted of three sets of ten repetitions for each prescribed exercise during both week one and weeks two to four. |
| **Progression** | Exercise intensity was progressively increased from approximately 70% to 80% of the estimated 1RM after the first week where tolerated. Individual adjustments were made according to each patient's physical capacity and training performance. |

**Nutritional intervention**

Patients received dietary counselling from a dietitian aimed at optimizing preoperative nutritional status through an adequate protein intake. Nutritional advice was individualized according to the patient's nutritional assessment.

**Six-minute walk test**

The 6MWT was performed according to the American Thoracic Society recommendations[2] on a 30-meter indoor course when possible. Participants were instructed to walk as far as possible within six minutes without running. They were permitted to slow down, stop, or rest if necessary and resume walking as soon as they were able. Standardized encouragement was provided at predetermined time points during the test. The total distance walked after six minutes was recorded in meters.

**Steep Ramp Test**

The Steep Ramp Test (SRT) was performed on a cycle ergometer. Following a three-minute warm-up at 0 W, the test started at a workload of 25 W, which was increased by 25 W every 10 seconds until volitional exhaustion. Participants were instructed to maintain a cadence of 70–80 revolutions per minute (rpm) and were verbally encouraged to continue for as long as possible. The test was terminated when the cadence dropped below 60 rpm. The maximum workload, total exercise time in seconds excluding the three-minute warm-up, and maximum heart rate were recorded. Maximum Short Exercise Capacity (MSEC) was calculated by multiplying the total exercise time in seconds by 2.5 W.

**References**

1. Garber C, Blissmer B, Deschenes M, Franklin B, Lamonte M, Lee I, et al. American College of Sports Medicine. American College of Sports Medicine position stand. Quantity and quality of exercise for developing and maintaining cardiorespiratory, musculoskeletal, and neuromotor fitness in apparently healthy adults: guidance for prescribing exercise. Med Sci Spors Exerc. 2011;43(7):1334–59. doi:10.1249/MSS.0b013e318213fefb.

2. ATS Committee on Proficiency Standards for Clinical Pulmonary Function Laboratories A. ATS statement: guidelines for the six-minute walk test. Am J Respir Crit Care Med. 2002;16(1):111–7. doi:10.1164/ajrccm.166.1.at1102.
