# Supplementary material for: Predicting which colorectal cancer patients are most likely to improve their functional capacity with pre-surgery prehabilitation: a retrospective study based on the 6-min walk distance
Source: Support Care Cancer. 2026 Jul 27;34(8):805. doi: 10.1007/s00520-026-11039-5 (PMC13407944; doi:10.1007/s00520-026-11039-5)
Supplement: Supplementary file 2 — (DOCX 67.8 KB) [file 520_2026_11039_MOESM2_ESM.docx]

Predicting which colorectal cancer patients are most likely to improve their functional capacity with pre-surgery prehabilitation: A retrospective study based on the six-minute walk distance. Supportive Care in Cancer. M. de Klerk, M.J.W. van der Linden, A.P.M. Kerckhoffs, B.R. Meijboom, E.G.G. Verdaasdonk, E. de Vries. Tranzo Scientific Centre for Care and Wellbeing, Tilburg School of Social and Behavioral Sciences, Tilburg University, Warandelaan 2 5037 AB Tilburg, The Netherlands, m.deklerk@tilburguniversity.edu

**Supplementary Information 2. Logical query used for patient selection and data extraction in CTcue**

In this supplementary, we provide a comprehensive dictionary for each query extracted from CTcue, detailing the mapping of Dutch terms and phrases to their English equivalents to ensure consistent interpretation and reproducibility across analyses.

**FIND PATIENTS**

INCLUDE patients

WHERE

((Appointment.Specialism = "Dietetiek"

AND Appointment.Description CONTAINS "NP voedingsadviezen binnen de prehabilitatie zorg"

AND Appointment.Status IN ["Voldaan", "Onbekend"]

AND Appointment.EndDate < '2023-09-01')

AND

(Appointment.Specialism = "Fysiotherapie"

AND Appointment.Description CONTAINS "Intake Pre Hab" OR "Pre Habilitatie"

AND Appointment.Status IN ["Voldaan", "Onbekend"]

AND Appointment.StartDate BETWEEN DietetiekAppointment.EndDate AND Surgery.StartDate)

AND

(Surgery.ProcedureCode IN ["034738", "034739", “034733”, “034735”, “034732”, “034734”, “034736”, “034024”, “034025”, “034023”, “034027”, “034026”, “334704C”, “334720C”, “334720”, “334704”, “334720G”, “334710B”, “334710”, “334704A”, “334704D”, “334702A”, “334702E”, “334720E”, “334730A”, “334703A”, “334703D”, “334703F”, “334703B”, “334704B”, “334702B”, “334702D”, “334702”, “334702C”, “334711D”, “334710A”, “334711F”, “334711”, “334711A”, “334711C”, “335711E”, “334720H”, “334720B”, “334720A”, “334720F”, “334810”, “334756”, “334730C”, “334731A”, “334824”, “334730E”, “334752L”, “334752R”, “334823”, 334828E”, “334822R”, “334828D”, “334821”, “334827”, “334828”, “334822L”, “334828B”, “334811”, “334814B”, “334759E”, “334755”, “335025C”, “334025C”, “335024C”, “335025B, “335015”, “335024A”, “335025F”, “335024”, “335025N”, “335025E”, “335025J”, “335024B”, “335024D”, “335023”, “335026A”, “335022”, “335025I”, “334759D”, “334649”, “334742”, “334761E”, “334742A”, “334750A”, “334793”, “334759B”, “334759A”, “334757D”, “034752”]

AND Surgery.Status NOT IN ["Geannuleerd", "Gepland", "Wachtlijst", "Peroperatief", "Geaccordeerd"]

AND Surgery.StartDate BETWEEN DietetiekAppointment.EndDate AND Surgery.StartDate))

EXCLUDE patients

WHERE (Consent.Policy = "Bezwaar med. dossier"

AND Consent.PermissionAnswer = "J")

*Dictionary*

| **Dutch** | **English** |
| --- | --- |
| Dietetiek | Dietetics |
| NP voedingsadviezen binnen de prehabilitatie zorg | Nutritional counselling within prehabilitation care |
| Voldaan | Completed |
| Onbekend | Unknown |
| Fysiotherapie | Physiotherapy |
| Intake Pre Hab / Pre Habilitatie | Prehabilitation intake |
| Geannuleerd | Cancelled |
| Gepland | Planned |
| Wachtlijst | Waiting list |
| Peroperatief | Peroperative |
| Geaccordeerd | Approved |
| Bezwaar med. dossier | Objection to use of medical record |

**COLLECT DATA**

*Medical history*

**Query 1 Myocardial Infarction**

DEFINE condition MyocardialInfarction AS

((Diagnosis.Description CONTAINS "myocardinfarct"

OR Diagnosis.Description CONTAINS "vroeger myocardinfarct"

OR Diagnosis.Description CONTAINS "acuut subendocardiaal myocardinfarct"

OR Diagnosis.Description CONTAINS “non-ST-elevatie myocardinfarct"

OR Diagnosis.Description CONTAINS "anteroseptal mycardinfarct”

OR Diagnosis.Description CONTAINS "infero-postero-lateraal myocardinfarct”

OR Diagnosis.Description CONTAINS "onderwand myocardinfarkt”

OR Diagnosis.Description CONTAINS "acuut inferolateraal myocardinfarct”

OR Diagnosis.Description CONTAINS "acuut myocardinfarct”

OR Diagnosis.Description CONTAINS "acuut myocardinfarkt”

OR Diagnosis.Description CONTAINS "voorwand myocardinfarkt”

OR Diagnosis.Description CONTAINS "anterolateral myocardinfarkt”

OR Diagnosis.Description CONTAINS "inferolateraal myocardinfarkt”

OR Diagnosis.Description CONTAINS "infero-posterior myocardinfarkt”

OR Diagnosis.Description CONTAINS "antero-septo-lateraal myocardinfarkt”

OR Diagnosis.Description CONTAINS "lateral myocardinfarkt”

OR Diagnosis.Description CONTAINS "onderwand myocardinfarkt beperkt”

OR Diagnosis.Description CONTAINS "voorwand myocardinfarkt beperkt”

OR Diagnosis.Description CONTAINS "posterior myocardinfarkt”

OR Diagnosis.Description CONTAINS "acuut transmuraal myocardinfarct lokalisatie niet gespecificeerd”

OR Diagnosis.Description CONTAINS "acuut transmuraal myocardinfarct van onderwand”

OR Diagnosis.Description CONTAINS "acuut myocardinfarct zonder ST-elevatie”

OR Diagnosis.Description CONTAINS "acuut transmuraal myocardinfarct van voorwand”

OR Diagnosis.Description CONTAINS "acuut myocardinfarct met ST-elevatie”

OR Diagnosis.Description CONTAINS "acuut myocardinfarct niet gespecificeerd”

OR Diagnosis.Description CONTAINS "acuut transmuraal myocardinfarct van overige gespecificeerde lokalisaties”

OR Diagnosis.Description CONTAINS "acuut myocardinfarct van voorwand”)

OR

(Diagnosis.Code IN ["I21.4", "I21.0", "I21.1", "I21.2", "I21.3", “I21.9”, “I21.0”, “I22.9”, “I22.0”, “I22.1”, “I22.8”, “I22.1”]))

WHERE

Diagnosis.StartDate < Surgery.Date

DEFINE condition MyocardInfarct_DCRA AS

(Form.Description = "DCRA"

AND FormResponse.Question = "Myocardinfarct"

AND FormResponse.Answer = "Yes)

*Dictionary*

| **Dutch** | **Engish** |
| --- | --- |
| Myocardinfarct/myocardinfarkt | myocardial infarction |
| vroeger | previous |
| acuut | acute |
| subendocardiaal | subendocardial |
| non-ST-elevatie | non-ST-elevation |
| anteroseptaal | anteroseptal |
| infero-postero-lateraal | inferoposterolateral |
| onderwand | inferior wall |
| inferolateraal | inferolateral |
| voorwand | anterior wall |
| anterolateraal | anterolateral |
| infero-posterior | inferoposterior |
| antero-septo-lateraal | anteroseptolateral |
| lateraal | lateral |
| beperkt | limited |
| posterior | posterior |
| transmuraal | transmural |
| lokalisatie niet gespecificeerd | location not specified |
| overige gespecificeerde lokalisaties | other specified locations |
| zonder ST-elevatie | without ST elevation |
| met ST-elevatie | with ST elevation |

**Query 2 Congestive Heart Failure**

DEFINE condition Congestive Heart Failure AS

((Diagnosis.Description CONTAINS "hartfalen"

OR Diagnosis.Description CONTAINS “decompensatio cordis”

OR Diagnosis.Description CONTAINS “chronisch hartfalen”

OR Diagnosis.Description CONTAINS “acuut hartfalen”

OR Diagnosis.Description CONTAINS “linkszijdig hartfalen”

OR Diagnosis.Description CONTAINS “rechtszijdig hartfalen”

OR Diagnosis.Description CONTAINS “diastolisch hartfalen”

OR Diagnosis.Description CONTAINS “linker hartfalen”

OR Diagnosis.Description CONTAINS “systolisch hartfalen”

OR Diagnosis.Description CONTAINS “congestief hartfalen”

OR Diagnosis.Description CONTAINS “acuut linkszijdig hartfalen”

OR Diagnosis.Description CONTAINS “acuut rechtszijdig hartfalen”

OR Diagnosis.Description CONTAINS “hypertensief acuut hartfalen”

OR Diagnosis.Description CONTAINS “decompensation cordis/hartfalen”

OR Diagnosis.Description CONTAINS “ischemisch bepaald hartfalen”

OR Diagnosis.Description CONTAINS “circulatoir hartfalen”

OR Diagnosis.Description CONTAINS “respiratoir hartfalen”

OR Diagnosis.Description CONTAINS “pre-terminaal hartfalen”

OR Diagnosis.Description CONTAINS “hartfalen door infusie”

OR Diagnosis.Description CONTAINS “start begeleiding hartfalenpolikliniek”

OR Diagnosis.Description CONTAINS “begeleiding op hartfalen-poli”

OR Diagnosis.Description CONTAINS “begeleiding op hartfalenpoli”

OR Diagnosis.Description CONTAINS “exacerbatie van chronisch hartfalen”

OR Diagnosis.Description CONTAINS “acuut linkszijdig congestief hartfalen”

OR Diagnosis.Description CONTAINS “pleura-effusie door congestief hartfalen”

OR Diagnosis.Description CONTAINS “rechtszijdig hartfalen door pulmonale hypertensie”

OR Diagnosis.Description CONTAINS “acuut hartfalen plaats niet nader gespecificeerd”

OR Diagnosis.Description CONTAINS “decompensatio cordis latent”

OR Diagnosis.Description CONTAINS “decompensatio cordis links”

OR Diagnosis.Description CONTAINS “ lichte decompensatio cordis”

OR Diagnosis.Description CONTAINS “links decompensatio cordis”

OR Diagnosis.Description CONTAINS “matige decompensatio cordis”

OR Diagnosis.Description CONTAINS “Ernstige decompensatio cordis”

OR Diagnosis.Description CONTAINS “rechts decompensatio cordis”

OR Diagnosis.Description CONTAINS “enige decompensatio cordis”

OR Diagnosis.Description CONTAINS “heropname decompensatio cordis”

OR Diagnosis.Description CONTAINS “chronische decompensatio cordis”

OR Diagnosis.Description CONTAINS “decompensatio cordis al dan niet na toediening van medicatie”

OR Diagnosis.Description CONTAINS “mogelijk decompensatio cordis”

OR Diagnosis.Description CONTAINS “opname ivm decompensatio cordis”

OR Diagnosis.Description CONTAINS “opname voor decompensatio cordis”

OR Diagnosis.Description CONTAINS “opname voor rechts- en links- decompensatio cordis”

OR Diagnosis.Description CONTAINS “links en rechts decompensatio cordis”

OR Diagnosis.Description CONTAINS “opname ivm decompensatio cordis en boezemfibrilleren”

OR Diagnosis.Description CONTAINS “status na decompensatio cordis”

OR Diagnosis.Description CONTAINS “enige rechts decompensatio cordis”

OR Diagnosis.Description CONTAINS “wsch. Links decompensatio cordis”

OR Diagnosis.Description CONTAINS “heropname ivm decompensatio cordis”

OR Diagnosis.Description CONTAINS “chronische rechts decompensatio cordis”

OR Diagnosis.Description CONTAINS “status na links decompensatio cordis”

OR Diagnosis.Description CONTAINS “Ernstige rechts decompensatio cordis”

OR Diagnosis.Description CONTAINS “gecompliceerd door decompensatio cordis”

OR Diagnosis.Description CONTAINS “dyspnoe wsch. li. decompensatio cordis”

OR

(Diagnosis.Code IN ["I43.1*”, “I43.8*”, “I50.1”, “I50.9”, “I50.0”, “I13.0”, “I11.0”. “I25.5”, “I42.0”, “I42.5”, “I42.6”, “I42.7”, “I42.8”, “I42.9”, “I13.2”, “P29.0”]))

WHERE

Diagnosis.StartDate < Surgery.Date

DEFINE condition Hartfalen DCRA AS

(Form.Description = "DCRA"

AND FormResponse.Question = "Congestief hartfalen"

AND FormResponse.Answer = "Yes)

*Dictionary*

| **Dutch** | **English** |
| --- | --- |
| hartfalen | heart failure |
| decompensatio cordis | cardiac decompensation |
| chronisch | chronic |
| acuut | acute |
| linkszijdig | left-sided |
| rechtszijdig | right-sided |
| diastolisch | diastolic |
| systolisch | systolic |
| congestief | congestive |
| hypertensief | hypertensive |
| ischemisch bepaald | ischemic in origin |
| circulatoir | circulatory |
| respiratoir | respiratory |
| pre-terminaal | preterminal |
| door infusie | due to infusion |
| begeleiding | management or follow up |
| Polikliniek/poli | outpatient clinic |
| exacerbatie | exacerbation |
| pleura-effusie | pleural effusion |
| door pulmonale hypertensie | due to pulmonary hypertension |
| plaats niet nader gespecificeerd | site not further specified |
| lichte | mild |
| matige | moderate |
| ernstige | severe |
| enige | some or mild |
| heropname | readmission |
| al dan niet na toediening van medicatie | with or without medication administration |
| mogelijk | possible |
| opname | admission |
| ivm | because of |
| status na | status after |
| wsch. | probably |
| gecompliceerd door | complicated by |
| dyspnoe | dyspnea |
| li. | left |

**Query 3 Peripheral Vascular Disease**

DEFINE condition Peripheral Vascular Disease AS

((Diagnosis.Description CONTAINS "perifeer vaatlijden"

OR Diagnosis.Description CONTAINS “perifeer arterieel vaatlijden”

OR Diagnosis.Description CONTAINS “perifere vaatlijden”

OR Diagnosis.Description CONTAINS “ernstig perifeer vaatlijden”

OR Diagnosis.Description CONTAINS “beenamputatie ivm vaatlijden”

OR Diagnosis.Description CONTAINS “gegeneraliseerd perifeer vaatlijden”

OR Diagnosis.Description CONTAINS “onderbeensamputatie ivm vaatlijden”

OR Diagnosis.Description CONTAINS “gegeneraliseerd vaatlijden”

OR Diagnosis.Description CONTAINS “bovenbeensamputatie ivm vaatlijden”

OR Diagnosis.Description CONTAINS “Ernstige perifere vaatlijden”

OR Diagnosis.Description CONTAINS “dilaterend arterieel vaatlijden”

OR Diagnosis.Description CONTAINS “inoperabel perifeer vaatlijden”

OR Diagnosis.Description CONTAINS “perifeer ischemisch vaatlijden”

OR Diagnosis.Description CONTAINS “perifeer arterieel vaatlijden Fontaine stadium 2”

OR Diagnosis.Description CONTAINS “perifeer arterieel vaatlijden Fontaine stadium 4”

OR Diagnosis.Description CONTAINS “verdenking op perifeer vaatlijden”

OR Diagnosis.Description CONTAINS “perifeer arterieel vaatlijden Fontaine stadium 3”

OR Diagnosis.Description CONTAINS “perifeer vaatlijden bij diabetes mellitus”

OR Diagnosis.Description CONTAINS “perifeer arterieel vaatlijden klasse 2”

OR Diagnosis.Description CONTAINS “verdenking op cardiovasculair genetisch risico bij aneurysmatisch vaatlijden”

OR Diagnosis.Description CONTAINS “perifeer arterieel vaatlijden klasse 1”

OR Diagnosis.Description CONTAINS “perifeer arterieel vaatlijden klasse 3”

OR Diagnosis.Description CONTAINS “claudicatio”

OR Diagnosis.Description CONTAINS “claudicatio intermittens”

OR Diagnosis.Description CONTAINS “claudicatio intermittens beiderzijds”

OR Diagnosis.Description CONTAINS “claudicatio intermittens links”

OR Diagnosis.Description CONTAINS “claudicatio intermittens rechts”

OR Diagnosis.Description CONTAINS “matige claudicatieklachten”

OR Diagnosis.Description CONTAINS “geringe claudicatieklachten”

OR Diagnosis.Description CONTAINS “geringe claudicatie klachten”

OR Diagnosis.Description CONTAINS “neurogene claudicatie”

OR Diagnosis.Description CONTAINS “neurogene claudicatio”

OR Diagnosis.Description CONTAINS “intermitterende neurogene claudicatio”

OR Diagnosis.Description CONTAINS “CABG”

OR Diagnosis.Description CONTAINS “CABG+Aortaklep+MitraalklepOK”

OR Diagnosis.Description CONTAINS “CABG veneuze grafts”

OR Diagnosis.Description CONTAINS “CABG en AortaklepOK”

OR Diagnosis.Description CONTAINS “CABG mbv LIMA”

OR Diagnosis.Description CONTAINS “re-CABG”

OR Diagnosis.Description CONTAINS “CABG + aneurysmectomie”

OR Diagnosis.Description CONTAINS “CABG en aneurysmectomie”

OR Diagnosis.Description CONTAINS “CABG mbv RIMA”

OR Diagnosis.Description CONTAINS “CABG LIMA en veneuze grafts”

OR Diagnosis.Description CONTAINS “hernieuwde CABG”

OR Diagnosis.Description CONTAINS “CABG en mitralisklep operatie”

OR Diagnosis.Description CONTAINS “AVR + CABG”

OR Diagnosis.Description CONTAINS “status na CABG”

OR Diagnosis.Description CONTAINS “CABG LIMA en RIMA”

OR Diagnosis.Description CONTAINS “CABG met LIMA RIMA en veneuze grafts”

OR Diagnosis.Description CONTAINS “CABG met LIMA en free RIMA”

OR Diagnosis.Description CONTAINS “CABG = 2 art. Grafts DBC”

OR Diagnosis.Description CONTAINS “re-CABG in Breda”

OR Diagnosis.Description CONTAINS “Re CABG operatie”

OR Diagnosis.Description CONTAINS “pericardeffusie na CABG”

OR Diagnosis.Description CONTAINS “re-CABG in Utrecht”

OR Diagnosis.Description CONTAINS “geaccepteerd voor CABG”

OR Diagnosis.Description CONTAINS “CABG met free LIMA en veneuze grafts”

OR Diagnosis.Description CONTAINS “CABG vene grafts en max. 1 arteriele graft DBC”

OR Diagnosis.Description CONTAINS “CABG RIMA en veneuze grafts”

OR Diagnosis.Description CONTAINS “frenicusparese links na CABG”

OR Diagnosis.Description CONTAINS “hoogstand linker diafragma na CABG”

OR Diagnosis.Description CONTAINS “post-CABG hoogstand linker diafragma”

OR Diagnosis.Description CONTAINS “femoro-popliteale bypass links”

OR Diagnosis.Description CONTAINS “femoro-popliteale bypass rechts”

OR Diagnosis.Description CONTAINS “femoropopliteale bypass”

OR Diagnosis.Description CONTAINS “bypass operatie in de klokkenberg”

OR Diagnosis.Description CONTAINS “bypass operatie: meerdere grafts”

OR Diagnosis.Description CONTAINS “bypass operatie met linker arteria mammaria”

OR Diagnosis.Description CONTAINS “bypass operatie”

OR Diagnosis.Description CONTAINS “bypass operatie: snake graft”

OR Diagnosis.Description CONTAINS “CAG na bypass-operatie”

OR Diagnosis.Description CONTAINS “bypass operatie in Eindhoven”

OR Diagnosis.Description CONTAINS “bypass met 1 prox.en 2 distale anast.”

OR Diagnosis.Description CONTAINS “bypass OK met 1 single graft”

OR Diagnosis.Description CONTAINS “bypassOK met 2 prox. En 3 distale anast.”

OR Diagnosis.Description CONTAINS “bypass met 2 veneuze prox dist anastom”

OR Diagnosis.Description CONTAINS “bypass OK met 1 prox.en 3 perif.anastom”

OR Diagnosis.Description CONTAINS “2e bypass operatie”

OR Diagnosis.Description CONTAINS “bypass operatie met single graft”

OR Diagnosis.Description CONTAINS “aanwezigheid van transplantaat voor aortocoronaire bypass”

OR Diagnosis.Description CONTAINS “follow-up na coronary-artery bypass graft”

OR Diagnosis.Description CONTAINS “aanwezigheid van bypass en anastomose van darm”

OR Diagnosis.Description CONTAINS “operatie met anastomose bypass of transplantaat”

OR Diagnosis.Description CONTAINS “onvoldoende funktie van de bypass”

OR Diagnosis.Description CONTAINS “PTCA van de bypass”

OR Diagnosis.Description CONTAINS “goede funktie van de bypass”

OR Diagnosis.Description CONTAINS “matige funktie van de bypass”

OR Diagnosis.Description CONTAINS “slechte funktie van de bypass”

OR Diagnosis.Description CONTAINS “redelijke funktie van de bypass”

OR Diagnosis.Description CONTAINS “PTCA van veneuze bypass graft”

OR Diagnosis.Description CONTAINS “percutane coronaire interventie”

OR Diagnosis.Description CONTAINS “coronaire bypass met de a. radialis”

OR Diagnosis.Description CONTAINS “status na percutane coronaire interventie”

OR Diagnosis.Description CONTAINS “status na coronaire bypass”

OR Diagnosis.Description CONTAINS “acuut myocardinfarct met ST-elevatie door occlusie van rechter coronaire arterie”

OR Diagnosis.Description CONTAINS “stenose van coronaire arterie”

OR Diagnosis.Description CONTAINS “aandoening van coronaire arterie”

OR Diagnosis.Description CONTAINS “occlusie van coronaire arterie”

OR Diagnosis.Description CONTAINS “follow-up na percutane transluminale coronaire angioplastiek”

OR Diagnosis.Description CONTAINS “PTCA”

OR Diagnosis.Description CONTAINS “PTCA LAD”

OR Diagnosis.Description CONTAINS “PTCA met drug-eluting-stent”

OR Diagnosis.Description CONTAINS “PTCA RCA”

OR Diagnosis.Description CONTAINS “PTCA RCX”

OR Diagnosis.Description CONTAINS “PTCA ramus circumflexus”

OR Diagnosis.Description CONTAINS “PTCA met bare-metal-stent”

OR Diagnosis.Description CONTAINS “re-PTCA”

OR Diagnosis.Description CONTAINS “PTCA diagonale tak”

OR Diagnosis.Description CONTAINS “PTCA in Utrecht”

OR Diagnosis.Description CONTAINS “PTCA MO-tak”

OR Diagnosis.Description CONTAINS “PTCA en aansluitend stemtimplantatie”

OR Diagnosis.Description CONTAINS “PTCA ramus descendens anterior”

OR Diagnosis.Description CONTAINS “PTCA ramus coronaira dextra”

OR Diagnosis.Description CONTAINS “CAG na PTCA”

OR Diagnosis.Description CONTAINS “re PTCA”

OR Diagnosis.Description CONTAINS “Rescue PTCA”

OR Diagnosis.Description CONTAINS “PTCA van 1e diagonale tak”

OR Diagnosis.Description CONTAINS “PTCA van de LAD met stent-plaatsing”

OR Diagnosis.Description CONTAINS “PTCA van de OM1-tak”

OR Diagnosis.Description CONTAINS “PTCA van de RCA met stent-plaatsing”

OR Diagnosis.Description CONTAINS “PTCA van recidief stenose”

OR Diagnosis.Description CONTAINS “PTCA van de anterolaterale tak”

OR Diagnosis.Description CONTAINS “PTCA van de hoofdstam”

OR Diagnosis.Description CONTAINS “re-PTCA van de LAD”

OR Diagnosis.Description CONTAINS “goed resultaat na eerder PTCA”

OR Diagnosis.Description CONTAINS “2e PTCA van de RCA”

OR Diagnosis.Description CONTAINS “2e PTCA van de ramus descendens ant.”

OR Diagnosis.Description CONTAINS “Dotter-procedure”

OR Diagnosis.Description CONTAINS “Dotter-behandeling aorta-bifurcatie”

OR Diagnosis.Description CONTAINS “PTA Dotter-dilatatie”

OR Diagnosis.Description CONTAINS “Dotter-behandeling arteria femoralis superficialis rechts”

OR Diagnosis.Description CONTAINS “Dotter-dilatatie van de arteria femoralis links”

OR Diagnosis.Description CONTAINS “Dotter-behandeling arteria iliaca communis rechts”

OR Diagnosis.Description CONTAINS “Dotter-dilatatie arteria iliaca links”

OR Diagnosis.Description CONTAINS “Dotter-dilatatie arteria iliaca communis rechts”

OR Diagnosis.Description CONTAINS “Dotter-dilatatie arteria iliaca communis links”

OR Diagnosis.Description CONTAINS “Dotter-dilatatie van de arteria femoralis rechts”

OR Diagnosis.Description CONTAINS “Dotterprocedure van een perifeer vat”

OR Diagnosis.Description CONTAINS “Dotter-dilatatie iliacale traject links”

OR Diagnosis.Description CONTAINS “Dotter-dilatatie arteria poplitea links”

OR Diagnosis.Description CONTAINS “Dotter-dilatatie arteria poplitea rechts”

OR Diagnosis.Description CONTAINS “Dotter-dilatatie arteria renalis stenose”

OR Diagnosis.Description CONTAINS “Dotter-dilatatie van diverse perifere vaten”

OR Diagnosis.Description CONTAINS “Dotterdilatatie arteria femoralis links”

OR Diagnosis.Description CONTAINS “Dotter van de nierarteriestenose rechts”

OR Diagnosis.Description CONTAINS “Dotter-behandeling met stent-plaatsing bdz in de arteria renalis”

OR Diagnosis.Description CONTAINS “Dotter-dilatatie iliacale traject rechts”

OR Diagnosis.Description CONTAINS “PTA Dotter van de arteria iliaca links”

OR Diagnosis.Description CONTAINS “PTA Dotter arteria iliaca links”

OR Diagnosis.Description CONTAINS “PTA Dotter van de femoralis links”

OR Diagnosis.Description CONTAINS “PTA Dotter-behandeling van de arteria femoralis links”

OR Diagnosis.Description CONTAINS “art. Renalisstenose re: dotterprocedure”

OR Diagnosis.Description CONTAINS “nabloeding lies bij dotter”

OR Diagnosis.Description CONTAINS “5^e^ dotter van de bypass”

OR Diagnosis.Description CONTAINS “mislukte dotter”

OR Diagnosis.Description CONTAINS “aneurysma”

OR Diagnosis.Description CONTAINS “aneurysma aortae abdominalis”

OR Diagnosis.Description CONTAINS “Aneurysma van hart”

OR Diagnosis.Description CONTAINS “aneurysma cordis”

OR Diagnosis.Description CONTAINS “aneurysma aorta abdominalis”

OR Diagnosis.Description CONTAINS “aneurysma aortae thoracalis”

OR Diagnosis.Description CONTAINS “aneurysma linker ventrikel”

OR Diagnosis.Description CONTAINS “aneurysma aortae”

OR Diagnosis.Description CONTAINS “Aneurysma van aorta abdominalis zonder vermelding van ruptuur”

OR Diagnosis.Description CONTAINS “Aneurysma van aorta thoracalis zonder vermelding van ruptuur”

OR Diagnosis.Description CONTAINS “Aneurysma en dissectie van arteria iliaca”

OR Diagnosis.Description CONTAINS “Aneurysma en dissectie van arterie van onderste extremiteit”

OR Diagnosis.Description CONTAINS “Aneurysma van aorta abdominalis met ruptuur”

OR Diagnosis.Description CONTAINS “Aneurysma van niet gespecificeerd deel van aorta zonder vermelding van ruptuur”

OR Diagnosis.Description CONTAINS “Aneurysma van de abdominale aorta”

OR Diagnosis.Description CONTAINS “Aneurysma en dissectie van overige gespecificeerde arterien”

OR Diagnosis.Description CONTAINS “Aneurysma en dissectie van niet gespecificeerde lokalisatie”

OR Diagnosis.Description CONTAINS “Aneurysma van aorta thoracalis”

OR Diagnosis.Description CONTAINS “Aneurysma en dissectie van arteria carotis”

OR Diagnosis.Description CONTAINS “Aneurysma en dissectie van coronairarterie”

OR Diagnosis.Description CONTAINS “Aneurysma van arteria iliaca”

OR Diagnosis.Description CONTAINS “Aneurysma van arteria poplitea”

OR Diagnosis.Description CONTAINS “Aneurysma en dissectie van arteria renalis”

OR Diagnosis.Description CONTAINS “Aneurysma en dissectie van arterie van bovenste extremiteit”

OR Diagnosis.Description CONTAINS “Aneurysma van aorta thoracalis met ruptuur”

OR Diagnosis.Description CONTAINS “Aneurysma van arteria femoralis”

OR Diagnosis.Description CONTAINS “Cerebraal aneurysma zonder ruptuur”

OR Diagnosis.Description CONTAINS “Operatie aneurysma aorta abdominalis”

OR Diagnosis.Description CONTAINS “Ruptuur van aneurysma aortae abdominalis”

OR

(Diagnosis.Code IN ["I70.0”, “I70.2”, “I70.8”, “I70.20”, “I70.21”, “I70.80”, “I70.90”. “I70.1”, “I70.9”, “I71.4”, “I71.3”, “I71.2”, “I71.9”, “I71.0”, “I71.1”, “I71.6”. “I71.8”, “I71.5”, “I73.0”, “I73.1”, “I73.9”, “I73.8”, “I77.1”, “I70.81”, “K55.1”, “K55.8”, “K55.9”, “Z95.8”, “Z95.9”, “I79.8”]))

WHERE

Diagnosis.StartDate < Surgery.Date

DEFINE condition Perifeer vaatlijden DCRA AS

(Form.Description = "DCRA"

AND FormResponse.Question = "Perifeer vaatlijden aneurysma aorta"

AND FormResponse.Answer = "Yes)

*Dictionary*

| **Dutch** | **English** |
| --- | --- |
| perifeer vaatlijden | peripheral vascular disease |
| perifeer arterieel vaatlijden | peripheral arterial disease |
| ernstig | severe |
| beenamputatie | leg amputation |
| gegeneraliseerd | generalized |
| onderbeensamputatie | lower leg amputation |
| bovenbeensamputatie | upper leg amputation |
| dilaterend | dilating |
| inoperabel | inoperable |
| ischemisch | ischemic |
| stadium | stage |
| verdenking op | suspected |
| klasse | class |
| cardiovasculair genetisch risico | cardiovascular genetic risk |
| claudicatio | claudication |
| claudicatio intermittens | intermittent claudication |
| beiderzijds | bilateral |
| geringe | mild |
| neurogene claudicatie | neurogenic claudication |
| CABG | coronary artery bypass graft |
| veneuze grafts | venous grafts |
| mitraalklep | mitral valve |
| LIMA | left internal mammary artery |
| RIMA | right internal mammary artery |
| aneurysmectomie | aneurysmectomy |
| pericardeffusie | pericardial effusion |
| diafragma | diaphragm |
| femoro popliteale bypass | femoropopliteal bypass |
| anastomose | anastomosis |
| darm | bowel |
| onvoldoende functie | insufficient function |
| redelijke functie | fair function |
| percutane coronaire interventie | percutaneous coronary intervention |
| a. radialis | radial artery |
| acuut myocardinfarct | acute myocardial infarction |
| occlusie | occlusion |
| stenose | stenosis |
| ramus circumflexus | circumflex branch |
| ramus descendens anterior | left anterior descending branch |
| dotter procedure | angioplasty |
| arteria femoralis | femoral artery |
| arteria iliaca | iliac artery |
| arteria poplitea | popliteal artery |
| arteria renalis | renal artery |
| nierarteriestenose | renal artery stenosis |
| funktie | function |
| aneurysma | aneurysm |
| aortae abdominalis | abdominal aorta |
| cordis | of the heart |
| aortae thoracalis | thoracic aorta |
| linker ventrikel | left ventricle |
| ruptuur | rupture |
| lokalisatie | location |
| arteria carotis | carotid artery |
| coronairarterie | coronary artery |
| arteria renalis | renal artery |
| bovenste extremiteit | upper extremity |
| cerebraal aneurysma | cerebral aneurysm |
| operatie aneurysma aorta abdominalis | abdominal aortic aneurysm repair |
| ruptuur van aneurysma aortae abdominalis | rupture of abdominal aortic aneurysm |

**Query 4 Cerebrovascular Disease**

DEFINE condition Cerebrovascular Disease AS

((Diagnosis.Description CONTAINS "CVA"

OR Diagnosis.Description CONTAINS “CVA met afasie”

OR Diagnosis.Description CONTAINS “cva ischemisch”

OR Diagnosis.Description CONTAINS “CVA-overig hersenen”

OR Diagnosis.Description CONTAINS “ischemisch CVA”

OR Diagnosis.Description CONTAINS “CVA met hemibeeld links”

OR Diagnosis.Description CONTAINS “bloedig CVA”

OR Diagnosis.Description CONTAINS “CVA met hemibeeld rechts”

OR Diagnosis.Description CONTAINS “beperkt CVA”

OR Diagnosis.Description CONTAINS “recidief CVA”

OR Diagnosis.Description CONTAINS “ischemisch CVA rechts”

OR Diagnosis.Description CONTAINS “CVA met hemiparese rechts en afasie”

OR Diagnosis.Description CONTAINS “pijn na CVA”

OR Diagnosis.Description CONTAINS “CVA met cerebellair syndroom”

OR Diagnosis.Description CONTAINS “CVA/beroerte nno”

OR Diagnosis.Description CONTAINS “CVA bloeding in hoofd overig”

OR Diagnosis.Description CONTAINS “CVA met passagere afasie”

OR Diagnosis.Description CONTAINS “CVA met visusstoornissen en afasie”

OR Diagnosis.Description CONTAINS “CVA door een geneesmiddel buiten de therapeutische range”

OR Diagnosis.Description CONTAINS “CVA na staken van medicatie”

OR Diagnosis.Description CONTAINS “CVA na toediening vitamine K ivm vaccinatie voor de tropen”

OR Diagnosis.Description CONTAINS “CVA rechts met rest hemibeeld links”

OR Diagnosis.Description CONTAINS “peroperatief klein CVA”

OR Diagnosis.Description CONTAINS “neurologische slikstoornissen door CVA”

OR Diagnosis.Description CONTAINS “analyse cardiale embolie-bron ivm een CVA”

OR Diagnosis.Description CONTAINS “bloedig CVA onder sintrommitis”

OR Diagnosis.Description CONTAINS “status na meerdere CVAs”

OR Diagnosis.Description CONTAINS “cerebrovasculair incident”

OR Diagnosis.Description CONTAINS “ischemisch cerebrovasculair accident”

OR Diagnosis.Description CONTAINS “bloedig cerebrovasculair accident”

OR Diagnosis.Description CONTAINS “cerebrovasculair accident door occlusive van hersenarterie”

OR Diagnosis.Description CONTAINS “cerebrovasculair accident met restverschijnselen in voorgeschiedenis”

OR Diagnosis.Description CONTAINS “cerebrovasculair accident in rechter hemisfeer”

OR Diagnosis.Description CONTAINS “cerebrovasculair accident van nog onbekende aard”

OR Diagnosis.Description CONTAINS “verandering in zintuiglijke waarneming als restverschijnsel na cerebrovasculair accident”

OR Diagnosis.Description CONTAINS “hemiparese door cerebrovasculair accident”

OR Diagnosis.Description CONTAINS “centrale pijn na cerebrovasculair accident”

OR Diagnosis.Description CONTAINS “hemiparese als restverschijnsel na cerebrovasculair accident”

OR Diagnosis.Description CONTAINS “epilepsie na cerebrovasculair accident”

OR Diagnosis.Description CONTAINS “onbloedig cerebrovasculair accident linker hemisfeer”

OR Diagnosis.Description CONTAINS “cathepsine A-gerelateerde arteriopathie met cerebrovasculaire accidenten en leukencefalopathie”

OR Diagnosis.Description CONTAINS “cognitieve stoornis als restverschijnsel na cerebrovasculair accident”

OR Diagnosis.Description CONTAINS “TIA”

OR Diagnosis.Description CONTAINS “TIA-storm”

OR Diagnosis.Description CONTAINS “TIA bij atriumfibrilleren”

OR Diagnosis.Description CONTAINS “TIA in anamneses”

OR Diagnosis.Description CONTAINS “recidiverende TIAs”

OR Diagnosis.Description CONTAINS “meerdere TIAs”

OR Diagnosis.Description CONTAINS “TIA met uitval links”

OR Diagnosis.Description CONTAINS “TIA met uitvalsverschijnselen rechts”

OR Diagnosis.Description CONTAINS “TIA met uitvalsverschijnselen links”

OR Diagnosis.Description CONTAINS “TIA met hemianopsie links”

OR Diagnosis.Description CONTAINS “transient cerebral ischaemic attack TIA, niet gespecificeerd”

OR Diagnosis.Description CONTAINS “overige gespecificeerde transient cerebral inschaemic attacks TIA en verwante syndromen”

OR Diagnosis.Description CONTAINS “status na recidiverende TIAs”

OR Diagnosis.Description CONTAINS “multipele TIAs in rechter hemisfeer”

OR Diagnosis.Description CONTAINS “transient ischemic attack”

OR Diagnosis.Description CONTAINS “transient ischaemic attack”

OR Diagnosis.Description CONTAINS “transient ischemic attack van vertebrobasilaris”

OR Diagnosis.Description CONTAINS “linkszijdig transient ischemic attack”

OR Diagnosis.Description CONTAINS “transient ischemic attack van voorste hersencirculatie”

OR Diagnosis.Description CONTAINS “rechtszijdig transient ischemic attack”

OR

(Diagnosis.Code IN ["G45.0”, “G45.1”, “G45.3”, “G45.4”, “G45.2”, “G45.9”, “G45.8”, “G46.2*”, “G46.7*”, “G46.7”, “G46.5*”, “G46.6*”, “G46.8”, “G46.4”, “G46.3*”, “G46.8*”, “G46.5”, “G46.6”, “G46.4*”, “G46.3”, “I60.9”, “I60.8”, “I60.6”, “I60.2”, “I60.1”, “I60.3”, “I60.4”, “I60.0”, “I60.7”, “I61.5”, “I61.9”, “I61.8”, “I61.2”, “I61.0”, "I61.1”, “I61.4”, “I61.6”, “I61.3”, “I62.0”, “I62.1”, “I62.9”, “I63.9”, “I63.5”, “I63.2”, “I63.8”, “I63.4”, “I63.4”, “I63.0”, “I63.6”, “I63.1”, “I64”, “I65.2”, “I65.0”, “I65.1”, “I65.8”, “I65.9”, “I65.3”, “I66.9”, “I66.0”, “I66.3”, “I66.2”, “I66.8”, “I66.1”, “I66.4”, “I67.5”, “I67.4”, “I67.2”, "I67.3”, “I67.8”, “I67.9”, “I67.1”, “I67.7”, “I67.0”, “I67.6”, “I68.0*”, “I68.8*”, “I68.0”, “I69.4”, “I69.4”, “I69.3”, “I69.8”, “I69.1”, “I69.0”, “I69.2”, “H34.0”, “H34.1”, “H34.2”]))

WHERE

Diagnosis.StartDate < Surgery.Date

DEFINE condition CVA of TIA DCRA AS

(Form.Description = "DCRA"

AND FormResponse.Question = "Cerebrovasculaire aandoening o.a. CVA TIA"

AND FormResponse.Answer = "Yes)

*Dictionary*

| **Dutch** | **English** |
| --- | --- |
| CVA | Cerebrovascular indicent/stroke |
| TIA | Transient Ischemic Attack |
| met afasie | with aphasia |
| ischemisch | ischemic |
| overig hersenen | other cerebral |
| hemibeeld | hemiparesis pattern |
| bloedig | hemorrhagic |
| beperkt | minor |
| recidief | recurrent |
| cerebellair syndroom | cerebellar syndrome |
| beroerte nno | stroke not otherwise specified |
| passagere afasie | transient aphasia |
| visusstoornissen | visual disturbances |
| door een geneesmiddel buiten de therapeutische range | due to drug outside therapeutic range |
| na staken van medicatie | after discontinuation of medication |
| peroperatief | intraoperative |
| neurologische slikstoornissen | neurologic swallowing disorder |
| analyse cardiale embolie-bron | evaluation of cardiac embolic source |
| onder sintrommitis | under acenocoumarol therapy |
| status na meerdere | history of multiple |
| cerebrovasculair incident | cerebrovascular incident |
| door occlusie van hersenarterie | due to occlusion of cerebral artery |
| met restverschijnselen | with residual symptoms |
| in rechter hemisfeer | in the right hemisphere |
| van nog onbekende aard | of yet unknown nature |
| verandering in zintuiglijke waarneming | change in sensory perception |
| centrale pijn | central pain |
| hemiparese | hemiparesis |
| epilepsie na | epilepsy after |
| onbloedig | non hemorrhagic |
| cathepsine A gerelateerde arteriopathie | cathepsin A related arteriopathy |
| leukencefalopathie | leukoencephalopathy |
| cognitieve stoornis | cognitive impairment |
| TIA storm | TIA storm |
| bij atriumfibrilleren | with atrial fibrillation |
| in anamneses | in history |
| recidiverende | recurrent |
| hemianopsie | hemianopia |
| transient cerebral ischaemic attack niet gespecificeerd | transient cerebral ischemic attack unspecified |
| overige gespecificeerde transient cerebral ischemic attacks | other specified transient cerebral ischemic attacks |
| vertebrobasilaris | vertebrobasilar |
| van voorste hersencirculatie | of the anterior cerebral circulation |
| cerebrovasculaire aandoening | cerebrovascular disease |

**Query 5 Dementia**

DEFINE condition Dementia AS

((Diagnosis.Description CONTAINS "dementie"

OR Diagnosis.Description CONTAINS “dementieel beeld”

OR Diagnosis.Description CONTAINS “Alzheimer-dementie”

OR Diagnosis.Description CONTAINS “dementieel syndroom”

OR Diagnosis.Description CONTAINS “niet gespecificeerde dementie”

OR Diagnosis.Description CONTAINS “dementie bij ziekte van Alzheimer niet gespecificeerd”

OR Diagnosis.Description CONTAINS “dementie bij ziekte van alzheimer atypisch of gemengd type”

OR Diagnosis.Description CONTAINS “dementie bij ziekte van Parkinson”

OR Diagnosis.Description CONTAINS “dementie bij overige gespecificeerde elders geclassificeerde ziekten”

OR Diagnosis.Description CONTAINS “vasculaire dementie”

OR Diagnosis.Description CONTAINS “dementie bij ziekte van Alzheimer”

OR Diagnosis.Description CONTAINS “lewy body-dementie”

OR Diagnosis.Description CONTAINS “subcorticale vasculaire dementie”

OR Diagnosis.Description CONTAINS “gemengde vasculaire dementie”

OR Diagnosis.Description CONTAINS “dementie bij ziekte van Pick”

OR Diagnosis.Description CONTAINS “multi-infarct dementie”

OR Diagnosis.Description CONTAINS “dementie door ziekte van Parkinson”

OR Diagnosis.Description CONTAINS “beginnende dementie”

OR Diagnosis.Description CONTAINS “frontotemporale dementie”

OR Diagnosis.Description CONTAINS “dementie bij laat optredende ziekte van Alzheimer”

OR Diagnosis.Description CONTAINS “dementie bij ziekte van Parkinson”

OR Diagnosis.Description CONTAINS “dementie bij ziekte van Alzheimer niet gespecificeerd”

OR Diagnosis.Description CONTAINS “dementie bij vroeg optredende ziekte van Alzheimer”

OR Diagnosis.Description CONTAINS “vasculaire dementie niet gespecificeerd”

OR Diagnosis.Description CONTAINS “gemengde corticale en subcorticale vormen van vasculaire dementie”

OR Diagnosis.Description CONTAINS “delirium niet gesuperponeerd op dementie en aldus beschreven”

OR Diagnosis.Description CONTAINS “delirium gesuperponeerd op dementie”

OR Diagnosis.Description CONTAINS “overige specificeerde vormen van vasculaire dementie”

OR Diagnosis.Description CONTAINS “mild cognitive impairment”

OR Diagnosis.Description CONTAINS “milde cognitieve stoornissen”

OR Diagnosis.Description CONTAINS “cognitieve stoornis”

OR Diagnosis.Description CONTAINS “cognitieve stoornissen”

OR Diagnosis.Description CONTAINS “lichte cognitieve stoornis”

OR Diagnosis.Description CONTAINS “belangrijke cognitieve stoornissen”

OR Diagnosis.Description CONTAINS “matige cognitieve achteruitgang”

OR Diagnosis.Description CONTAINS “cognitieve stoornis als restverschijnsel na cerebrovasculair accident”

OR Diagnosis.Description CONTAINS “cognitieve functiestoornis van taal”

OR Diagnosis.Description CONTAINS “Ernstige cognitieve achteruitgang”

OR Diagnosis.Description CONTAINS “leeftijdsgerelateerde cognitieve achteruitgang”

OR Diagnosis.Description CONTAINS “cognitieve veranderingen door organische stoornis”

OR Diagnosis.Description CONTAINS “verdenking op cognitieve stoornis”

OR Diagnosis.Description CONTAINS “alzheimerdementie met gedragsstoornis”

OR Diagnosis.Description CONTAINS “Alzheimer-dementie laat begin”

OR Diagnosis.Description CONTAINS “ziekte van Alzheimer”

OR Diagnosis.Description CONTAINS “morbus Alzheimer”

OR Diagnosis.Description CONTAINS “Alzheimer-dementie laat begin met delier”

OR Diagnosis.Description CONTAINS “Alzheimer-dementie met progressieve afasie”

OR Diagnosis.Description CONTAINS “Ziekte van Alzheimer niet gespecificeerd”

OR Diagnosis.Description CONTAINS “Overige gespecificeerde vormen van ziekte van Alzheimer”

OR Diagnosis.Description CONTAINS “Vroeg optredende ziekte van Alzheimer”

OR Diagnosis.Description CONTAINS “Laat optredende ziekte van Alzheimer”

OR Diagnosis.Description CONTAINS “Dementie bij laat optredende ziekte van Alzheimer”

OR Diagnosis.Description CONTAINS “Dementie bij vroeg optredende ziekte van Alzheimer”

OR Diagnosis.Description CONTAINS “Dementie bij ziekte van Alzheimer atypisch of gemengd type”
 OR Diagnosis.Description CONTAINS “Familiaire ziekte van Alzheimer”

OR Diagnosis.Description CONTAINS “uitgebreide neurocognitieve stoornis door de ziekte van alzheimer met gedragsstoornissen”

OR Diagnosis.Description CONTAINS “uitgebreide neurocognitieve stoornis door de ziekte van alzheimer met gedragsstoornissen”

OR Diagnosis.Description CONTAINS “dementie bij laat optredende ziekte van Alzheimer”

OR Diagnosis.Description CONTAINS “dementie bij ziekte van Alzheimer niet gespecificeerd”

OR Diagnosis.Description CONTAINS “vroege ziekte van Alzheimer”

OR Diagnosis.Description CONTAINS “ziekte van Alzheimer laat begin met delier”

OR

(Diagnosis.Code IN ["F00.9*”, “F00.2*”, “F00.1*”, “F00.9”, “F00.0*”, “F00.1”, “F00.0”, “F00.9”, “F01.2”, “F01.1”, “F01.9”, “F01.3”, “F01.8”, “F01.0”, “F02.3*”, “F02.8*”, “F02.0*”, “F02.3”, “F02.0”, “F02.2*”, “F03”, “G30.9”, “G30.8”, “G30.0”, “G30.1”, “F05.1”, “G31.1”, “F04”, “F06.1”, “F06.8”, “G31.0”, “G31.2”, “G94.3”, “G94.8”, "G94.1*”, “G94.0*”, “G94.2*”, “R54”, “R41.8”, “R41.1]))

WHERE

Diagnosis.StartDate < Surgery.Date

DEFINE condition Dementie DCRA AS

(Form.Description = "DCRA"

AND FormResponse.Question = "Dementie"

AND FormResponse.Answer = "Yes)

*Dictionary*

| **Dutch** | **English** |
| --- | --- |
| dementie | dementia |
| cognitieve stoornis | cognitive impairment |
| Alzheimer | Alzheimer |
| dementieel beeld | demential presentation |
| dementieel syndroom | demential syndrome |
| niet gespecificeerde | unspecified |
| atypisch of gemengd type | atypical or mixed type |
| frontotemporale | frontotemporal |
| Lewy body | Lewy body |
| subcorticale vasculaire | subcortical vascular |
| gemengde vasculaire | mixed vascular |
| multi-infarct | multi-infarct |
| beginnende | early-stage |
| vroeg optredende ziekte van | early-onset |
| laat optredende ziekte van | late-onset |
| mild | mild |
| milde | mild |
| lichte | mild |
| matige | moderate |
| ernstige | severe |
| leeftijdsgerelateerde | age-related |
| door organische stoornis | due to organic disorder |
| verdenking op | suspected |
| met gedragsstoornis | with behavioral disorder |
| uitgebreide neurocognitieve | major neurocognitive |
| met gedragsstoornissen | with behavioral disturbances |
| delirium niet gesuperponeerd op | delirium not superimposed on |
| delirium gesuperponeerd op | delirium superimposed on |

**Query 6 Chronic Pulmonary Disease**

DEFINE condition Chronic Pulmonary Disease AS

((Diagnosis.Description CONTAINS "COPD"

OR Diagnosis.Description CONTAINS “COPD GOLD II”

OR Diagnosis.Description CONTAINS “COPD GOLD III”

OR Diagnosis.Description CONTAINS “COPD GOLD IV”

OR Diagnosis.Description CONTAINS “COPD GOLD I”

OR Diagnosis.Description CONTAINS “COPD in anamnese”

OR Diagnosis.Description CONTAINS “COPD met OSAS”

OR Diagnosis.Description CONTAINS “astma-COPD-overlapsyndroom”

OR Diagnosis.Description CONTAINS “exacerbatie COPD”

OR Diagnosis.Description CONTAINS “enig COPD”

OR Diagnosis.Description CONTAINS “CARA/COPD”

OR Diagnosis.Description CONTAINS “acute exacerbatie van COPD”

OR Diagnosis.Description CONTAINS “opname ivm exacerbatie COPD”

OR Diagnosis.Description CONTAINS “chronisch obstructief longlijden”

OR Diagnosis.Description CONTAINS “chronisch obstruktief longlijden”

OR Diagnosis.Description CONTAINS “chronische obstructieve longaandoening niet gespecificeerd”

OR Diagnosis.Description CONTAINS “chronische obstructieve longaandoening niet gespecifieerd GOLD2”

OR Diagnosis.Description CONTAINS “chronische obstructieve longaandoening met acute exacerbatie niet gespecificeerd GOLD-NNO”

OR Diagnosis.Description CONTAINS “chronische obstructieve longaandoening met acute exacerbatie niet gespecificeerd”

OR Diagnosis.Description CONTAINS “chronische obstructieve longaandoening niet gespecificeerd GOLD3”

OR Diagnosis.Description CONTAINS “chronische obstructieve longaandoening met acute infectie van onderste luchtwegen”

OR Diagnosis.Description CONTAINS “chronische obstructieve longaandoening met acute exacerbatie niet gespecificeerd GOLD2”

OR Diagnosis.Description CONTAINS “chronische obstructieve longaandoening niet gespecificieerd GOLD1”

OR Diagnosis.Description CONTAINS “chronische obstructieve longaandoening met acute exacerbatie niet gespecificeerd GOLD4”

OR Diagnosis.Description CONTAINS “chronische obstructieve longaandoening niet gespecificeerd GOLD4”

OR Diagnosis.Description CONTAINS “chronische obstructieve longaandoening met acute infectie van onderste luchtwegen GOLD3”

OR Diagnosis.Description CONTAINS “chronische obstructieve longaandoening met acute infectie van onderste luchtwegen GOLD-NNO”

OR Diagnosis.Description CONTAINS “chronische obstructieve longaandoening met acute infectie van onderste luchtwegen GOLD2”

OR Diagnosis.Description CONTAINS “chronische obstructieve longaandoening met acute infectie van onderste luchtwegen GOLD4”

OR Diagnosis.Description CONTAINS “chronische obstructieve longaandoening met acute infectie van onderste luchtwegen GOLD1”

OR Diagnosis.Description CONTAINS “chronische obstructieve longaandoening met acute exacerbatie niet gespecificeerd GOLD1”

OR Diagnosis.Description CONTAINS “overige gespecificeerde chronische obstructieve luchtwegaandoeningen GOLD2”

OR Diagnosis.Description CONTAINS “overige gespecificeerde chronische obstructieve luchtwegaandoeningen”

OR Diagnosis.Description CONTAINS “overige gespecificeerde chronische obstructieve luchtwegaandoeningen GOLD3”

OR Diagnosis.Description CONTAINS “overige gespecificeerde chronische obstructieve luchtwegaandoeningen GOLD1”

OR Diagnosis.Description CONTAINS “overige gespecificeerde chronische obstructieve luchtwegaandoeningen GOLD-NNO”

OR Diagnosis.Description CONTAINS “overige gespecificeerde chronische obstructieve luchtwegaandoeningen GOLD4”

OR

(Diagnosis.Code IN ["J40”, “J41.1”, “J42”, “J43.9”, “J43.2”, “J43.1”, “J43.8”, “J44.9”, “J44.99”, “J44.92”, “J44.19”, “J44.1”, “J44.93”, “J44.91”, “J44.13”, “J44.0”, “J44.14”, “J44.12”, “J44.09”, “J44.94”, “J44.03”, “J44.02”, “J44.04”, “J44.82”, “J44.8”, “J44.83”, “J44.81”, “J44.01”, “J44.11”, “J44.89”, “J44.84”, “J45.9”, “J45.0”, “J45.1”, "J45.8”, “J46”, “J47”, “J60”, “J61”, “J62.8”, “J64”, “J66.8”, “J67.2”, “J67.5”, “J67.9”, “J67.8”, “J68.4”, “J70.1”]))

WHERE

Diagnosis.StartDate < Surgery.Date

DEFINE condition Chronische longziekte DCRA AS

(Form.Description = "DCRA"

AND FormResponse.Question = "Chronische longziekte"

AND FormResponse.Answer = "Yes)

*Dictionary*

| **Dutch** | **English** |
| --- | --- |
| COPD | COPD |
| GOLD I | GOLD I |
| GOLD II | GOLD II |
| GOLD III | GOLD III |
| GOLD IV | GOLD IV |
| in anamnese | in history |
| met OSAS | with OSAS |
| astma-overlapsyndroom | asthma-overlap syndrome |
| exacerbatie | exacerbation |
| enig | single |
| acute | acute |
| opname ivm exacerbatie | admission due to exacerbation |
| chronisch obstructief longlijden | chronic obstructive pulmonary disease |
| niet gespecificeerd | unspecified |
| met acute infectie van onderste luchtwegen | with acute lower respiratory tract infection |
| overige gespecificeerde | other specified |

**Query 7 Rheumatic Disease**

DEFINE condition Rheumatic Disease AS

((Diagnosis.Description CONTAINS "systeemziekte van bindweefsel"

OR Diagnosis.Description CONTAINS “ongedifferentieerde bindweefsel ziekte”

OR Diagnosis.Description CONTAINS “systeemziekte van bindweefsel niet gespecificeerd”

OR Diagnosis.Description CONTAINS “overige gespecificeerde systeemziekten van bindweefsel”

OR Diagnosis.Description CONTAINS “polyneuropathie bij systeemziekten van bindweefsel”

OR Diagnosis.Description CONTAINS “gelokaliseerde bindweefselaandoening niet gespecificeerd”

OR Diagnosis.Description CONTAINS “reumatoide artritis”

OR Diagnosis.Description CONTAINS “reumatoide arthritis”

OR Diagnosis.Description CONTAINS “reumatoide artritis niet gespecificeerd van lokalisatie niet gespecificeerd”

OR Diagnosis.Description CONTAINS “reumatoide artritis niet gespecificeerd van multipele lokalisaties”

OR Diagnosis.Description CONTAINS “reumatoide artritis niet gespecificeerd van onderbeen”

OR Diagnosis.Description CONTAINS “reumatoide artritis niet gespecificeerd van enkel en voet”

OR Diagnosis.Description CONTAINS “reumatoide artritis niet gespecificeerd van onderarm”

OR Diagnosis.Description CONTAINS “reumatoide artritis met aandoening van overige organen en orgaansystemen van lokalisatie niet gespecificeerd”

OR Diagnosis.Description CONTAINS “reumatoide artritis van knie”

OR Diagnosis.Description CONTAINS “reumatoide artritis van voet”

OR Diagnosis.Description CONTAINS “overige autoimmuun-/reumatologische aandoeningen”

OR Diagnosis.Description CONTAINS “reumatoide vasculitis van lokalisatie niet gespecificeerd”

OR Diagnosis.Description CONTAINS “reumatoide artritis van hand”

OR Diagnosis.Description CONTAINS “reumatoide nodulus van lokalisatie niet gespecificeerd”

OR Diagnosis.Description CONTAINS “reumatoide artritis niet gespecificeerd van bovenarm”

OR Diagnosis.Description CONTAINS “reumatoide artritis niet gespecificeerd van hand”

OR Diagnosis.Description CONTAINS “seropositieve reumatoide artritis”

OR Diagnosis.Description CONTAINS “reumatoide artritis niet gespecificeerd van schoudergebied”

OR Diagnosis.Description CONTAINS “seropositieve reumatoide artritis niet gespecificeerd van lokalisatie niet gespecificeerd”

OR Diagnosis.Description CONTAINS “seronegatieve reumatoide artritis van lokalisatie niet gespecificeerd”

OR Diagnosis.Description CONTAINS “juveniele reumatoide artritis van lokalisatie niet gespecificeerd”

OR Diagnosis.Description CONTAINS “reumafactor-positieve en antiCCP-positieve reumatoide artritis”

OR Diagnosis.Description CONTAINS “reuma niet gespecificeerd van lokalisatie niet gespecificeerd”

OR Diagnosis.Description CONTAINS “polymyalgia reumatica”

OR Diagnosis.Description CONTAINS “reumafactor negatieve en antiCCP negatieve reumatoide artritis”

OR Diagnosis.Description CONTAINS “overige gespecificeerde seropositieve reumatoide artritis van lokalisatie niet gespecificeerd”

OR Diagnosis.Description CONTAINS “gonartrose bij reumatische aandoening”

OR Diagnosis.Description CONTAINS “overige gespecificieerde reumatoide artritis van lokalisatie niet gespecificeerd”

OR Diagnosis.Description CONTAINS “reumatische aortastenose”

OR Diagnosis.Description CONTAINS “acuut reuma zonder vermelding van hartaandoening”

OR

(Diagnosis.Code IN ["M05”, “M05.99”, “M05.89”, “M05.39+”, “M05.29”, “M05.90”, “J99.0*”, “M05.19+”, “M05.09”, “M05.84”, “M05.28”, “M05.80”, “M05.30+”, “M05.98”, “M05.94”, “M05.83”, “M05.32+”, “M05.38+”, “M05.18+”, “M05.9”, “M05.97”, “M05.93”, “M32.9”, “M32.1”, “M32.8”, “M32.0”, “M33.2”, “M33.1”, “M33.0”, “M33.9”, “M34.0”, “M34”, “M34.9”, “M34.8”, "M34.1”, “M06”, “M06.99”, “M06.90”, “M06.09”, “M06.96”, “M06.97”, “M06.19”, “M06.89”, “M06.93”, “M06.39”, “M06.00”, “M06.92”, “M06.91”, “M06.94”, “M06.49”, “M06.44”, “M06.34”, “M06.98”, “M06.43”, “M06.80”, “M06.95”, “M06.35”, “M06.9”, “M06.32”, “M06.33”, “M06.29”, “M06.37”, “M06.30”, “M06.08”, “M06.04”, “M031.5”, “M35.3”, “M36.0*”, “M31.5”]))

WHERE

Diagnosis.StartDate < Surgery.Date

DEFINE condition Bindweefselziekte DCRA AS

(Form.Description = "DCRA"

AND FormResponse.Question = "Bindweefselziekte o.a. reumatoide ziekten"

AND FormResponse.Answer = "Yes)

*Dictionary*

| **Dutch** | **English** |
| --- | --- |
| systeemziekte van bindweefsel | systemic connective tissue disease |
| ongedifferentieerde bindweefsel ziekte | undifferentiated connective tissue disease |
| niet gespecificeerd | unspecified |
| overige gespecificeerde systeemziekten van bindweefsel | other specified systemic connective tissue diseases |
| polyneuropathie bij systeemziekten van bindweefsel | polyneuropathy in connective tissue disease |
| gelokaliseerde bindweefselaandoening | localized connective tissue disorder |
| reumatoide artritis | rheumatoid arthritis |
| van knie | of the knee |
| van voet | of the foot |
| van hand | of the hand |
| van bovenarm | of the upper arm |
| van onderarm | of the forearm |
| van enkel en voet | of ankle and foot |
| van schoudergebied | of shoulder region |
| met aandoening van overige organen en orgaansystemen | with involvement of other organs and organ systems |
| seropositief | seropositive |
| seronegatief | seronegative |
| juveniel | juvenile |
| reumafactor-positief en antiCCP-positief | rheumatoid factor positive and anti-CCP positive |
| reumafactor-negatief en antiCCP-negatief | rheumatoid factor negative and anti-CCP negative |
| polymyalgia reumatica | polymyalgia rheumatica |
| overige gespecificeerde seropositieve | other specified seropositive |
| overige gespecificeerde | other specified |
| gonartrose bij reumatische aandoening | gonarthrosis in rheumatic disorder |
| reumatische aortastenose | rheumatic aortic stenosis |
| acuut reuma zonder vermelding van hartaandoening | acute rheumatism without mention of heart disease |

**Query 8 Peptic Ulcer Disease**

DEFINE condition Peptic Ulcer Disease AS

((Diagnosis.Description CONTAINS "ulcus maag duodenum"

OR Diagnosis.Description CONTAINS “ulcus pepticum”

OR Diagnosis.Description CONTAINS “recidiverend ulcus pepticum”

OR Diagnosis.Description CONTAINS “ulcus pepticum lokalisatie niet gespecifieerd acuut met bloeding”

OR Diagnosis.Description CONTAINS “ulcus pepticum lokalisatie niet gespecificeerd niet gespecificeerd als acuut of chronisch zonder bloeding of perforatie”

OR Diagnosis.Description CONTAINS “ulcus pepticum lokalisatie niet gespecificeerd chronisch of niet gespecificeerd met bloeding”

OR Diagnosis.Description CONTAINS “acute bloeding van ulcus pepticum”

OR Diagnosis.Description CONTAINS “ulcus pepticum van maag”

OR Diagnosis.Description CONTAINS “ulcus pepticum acuut met bloeding”

OR Diagnosis.Description CONTAINS “ulcus pepticum acuut met perforatie”

OR Diagnosis.Description CONTAINS “ulcus pepticum lokalisatie niet gespecificeerd chronisch of niet gespecificeerd met perforatie”

OR Diagnosis.Description CONTAINS “ulcus pepticum of complicatie van ulcera”

OR Diagnosis.Description CONTAINS “operatie ivm ulcus pepticum”

OR Diagnosis.Description CONTAINS “ulcus pepticum acuut zonder bloeding of perforatie”

OR Diagnosis.Description CONTAINS “maagbloeding”

OR Diagnosis.Description CONTAINS “opname ivm een maagbloeding”

OR

(Diagnosis.Code IN ["K25.9”, “K25.4”, “K25.1”, “K25.5 “K25.3”, “K25.7”, “K25.0”, “K25.6”, “K25.2”, “K26”, “K26.9”, “K26.4”, “K26.1”, “K26.3”, “K26.5”, “K26.7”, “K26.0”, “K26.6”, “K27.0”, “K27.9”, “K27.4”, “K27.5”, “K28.1”, “K28.9”, “K28.4”, “K28.3”, “K28.7”, “K28.5”]))

WHERE

Diagnosis.StartDate < Surgery.Date

DEFINE condition Maagzweer DCRA AS

(Form.Description = "DCRA"

AND FormResponse.Question = "gastrointestinal ulcuslijden"

AND FormResponse.Answer = "Yes)

*Dictionary*

| **Dutch** | **English** |
| --- | --- |
| Maagzweer / ulcus pepticum | peptic ulcer |
| ulcus maag duodenum | stomach/duodenal ulcer |
| recidiverend | recurrent |
| lokalisatie niet gespecificeerd | unspecified location |
| acuut | acute |
| chronisch | chronic |
| met bloeding | with bleeding |
| zonder bloeding | without bleeding |
| met perforatie | with perforation |
| complicatie van ulcera | complication of ulcers |
| operatie ivm | surgery due to |
| maagbloeding | gastric bleeding |
| opname ivm een maagbloeding | hospitalization due to gastric bleeding |

**Query 9 Liver disease**

DEFINE condition Mild Liver Disease AS

((Diagnosis.Description CONTAINS "levercirrose"

OR Diagnosis.Description CONTAINS “levercirrhosis”

OR Diagnosis.Description CONTAINS “levercirrhose”

OR Diagnosis.Description CONTAINS “levercirrose NNO”

OR Diagnosis.Description CONTAINS “levercirrose fibrose gedecompenseerd”

OR Diagnosis.Description CONTAINS “alcoholische levercirrose”

OR Diagnosis.Description CONTAINS “alcoholische levercirrose gedecompenseerd”

OR Diagnosis.Description CONTAINS “gedecompenseerde levercirrose”

OR Diagnosis.Description CONTAINS “gecompenseerde levercirrose”

OR Diagnosis.Description CONTAINS “alcoholische levercirrose gecompenseerd”

OR Diagnosis.Description CONTAINS “overige gespecificeerde en niet gespecificeerde levercirrose”

OR Diagnosis.Description CONTAINS “toxische leverziekte met leverfibrose en levercirrose”

OR Diagnosis.Description CONTAINS “portale hypertensie”

OR Diagnosis.Description CONTAINS “chronische hepatitis C”

OR Diagnosis.Description CONTAINS “chronische hepatitis B”

OR Diagnosis.Description CONTAINS “chronische hepatitis”

OR Diagnosis.Description CONTAINS “chronische autoimmuun hepatitis”

OR Diagnosis.Description CONTAINS “chronische persisterende hepatitis”

OR Diagnosis.Description CONTAINS “chronische hepatitis niet gespecificeerd”

OR Diagnosis.Description CONTAINS “chronische hepatitis B zonder Delta-agens”

OR Diagnosis.Description CONTAINS “chronische actieve hepatitis niet elders geclassificeerd”

OR Diagnosis.Description CONTAINS “chronische actieve hepatitis B”

OR Diagnosis.Description CONTAINS “chronische hepatitis B met Delta-agens”

OR Diagnosis.Description CONTAINS “chronische persisterende hepatitis niet elders geclassificeerd”

OR Diagnosis.Description CONTAINS “chronische actieve hepatitis C”

OR Diagnosis.Description CONTAINS “chronische persisterende hepatitis B”

OR Diagnosis.Description CONTAINS “chronische lobulaire hepatitis niet elders geclassificeerd”

OR Diagnosis.Description CONTAINS “chronische lobulaire hepatitis B”

OR Diagnosis.Description CONTAINS “chronische persisterende hepatitis C”

OR Diagnosis.Description CONTAINS “overige gespecificeerde chronische hepatitis niet elders geclassificeerd”

OR Diagnosis.Description CONTAINS “toxische leverziekte met chronische actieve hepatitis”

OR

(Diagnosis.Code IN ["B18.2”, “B18.1”, “B18.19”, “B18.8”, “B18.9”, “B18.0”, “K73.9”, “K73.2”, “K73.8”, “K73.0”, “K73.1”, “K74.0”, “K74.1”, “K74.3”, “K74.6”, “K74.5”, “K70.0”, “K70.1”, “K70.2”, “K70.3”, “K70.9”, “K71.7”, “K71.5”, “K76.0”, “K76.2”, “K76.3”, “K76.4”, “K76.8”, “K76.9”, “Z94.4”]))

WHERE

Diagnosis.StartDate < Surgery.Date

DEFINE condition Leverziekte DCRA AS

(Form.Description = "DCRA"

AND FormResponse.Question = "Leverziekte"

AND FormResponse.Answer = "Yes)

DEFINE condition Moderate of severe Liver Disease AS

(Diagnosis.Code IN "I85.0”, “K70.4”, “K71.1”, “K72.1”, “K72.9”, “K76.6”, “K76.7”, “Z94.4”])

WHERE

Diagnosis.StartDate < Surgery.Date

*Dictionary*

| **Dutch** | **English** |
| --- | --- |
| Leverziekte / levercirrose | liver disease / cirrhosis |
| levercirrhose | cirrhosis |
| NNO | unspecified |
| fibrose gedecompenseerd | fibrotic decompensated |
| alcoholische | alcoholic |
| gedecompenseerd | decompensated |
| gecompenseerd | compensated |
| overige gespecificeerde en niet gespecificeerde | other specified and unspecified |
| toxische leverziekte | toxic liver disease |
| met leverfibrose en levercirrose | with fibrosis and cirrhosis |
| portale hypertensie | portal hypertension |
| chronische hepatitis (B/C) | chronic hepatitis (B/C) |
| chronische autoimmuun hepatitis | chronic autoimmune hepatitis |
| chronische persisterende hepatitis | chronic persistent hepatitis |
| chronische hepatitis niet gespecificeerd | chronic hepatitis unspecified |
| zonder Delta-agens | without delta agents |
| niet elders geclassificeerd | not elsewhere classified |
| actieve | active |
| lobulaire | lobular |

**Query 10 Diabetes Mellitus**

DEFINE condition Diabetes Without Chronic Complications AS

((Diagnosis.Description CONTAINS "diabetes"

OR Diagnosis.Description CONTAINS “diabetes mellitus”

OR Diagnosis.Description CONTAINS “diabetes mellitus type 2”

OR Diagnosis.Description CONTAINS “diabetes mellitus type II”

OR Diagnosis.Description CONTAINS “diabetes mellitus type 1”

OR Diagnosis.Description CONTAINS “niet insuline-afhankelijke diabetes”

OR Diagnosis.Description CONTAINS “type 2 diabetes mellitus zonder complicaties”

OR Diagnosis.Description CONTAINS “niet gespecificeerde diabetes mellitus zonder complicaties”

OR Diagnosis.Description CONTAINS “niet gespecificeerde diabetes mellitus met oogcomplicaties”

OR Diagnosis.Description CONTAINS “niet gespecificeerde diabetes mellitus met niet gespecificeerde complicaties”

OR Diagnosis.Description CONTAINS “niet gespecificeerde diabetes mellitus met complicaties van perifere circulatie”

OR Diagnosis.Description CONTAINS “type 1 diabetes mellitus zonder complicaties”

OR Diagnosis.Description CONTAINS “type 2 diabetes mellitus met niet gespecificeerde complicaties”

OR Diagnosis.Description CONTAINS “type 1 diabetes mellitus met niet gespecificeerde complicaties”

OR Diagnosis.Description CONTAINS “niet gespecificeerde diabetes mellitus met neurologische complicaties”

OR Diagnosis.Description CONTAINS “type 2 diabetes mellitus met oogcomplicaties”

OR Diagnosis.Description CONTAINS “type 2 diabetes mellitus met neurologische complicaties”

OR Diagnosis.Description CONTAINS “glomerulaire aandoeningen bij diabetes mellitus”

OR Diagnosis.Description CONTAINS “gecompliceerde diabetes mellitus type 2”

OR Diagnosis.Description CONTAINS “type 2 diabetes met niercomplicaties”

OR Diagnosis.Description CONTAINS “overige gespecificeerde vormen van diabetes mellitus zonder complicaties”

OR Diagnosis.Description CONTAINS “reeds bestaande type 1 diabetes mellitus”

OR Diagnosis.Description CONTAINS “niet gespecificeerde diabetes mellitus met ketoacidose”

OR Diagnosis.Description CONTAINS “reeds bestaande type 2 diabetes mellitus”

OR Diagnosis.Description CONTAINS “type 1 diabetes mellitus met oogcomplicaties”

OR

(Diagnosis.Code IN "E10.0”, “E10.1”, “E10.8”, “E10.9”, “E11.0”, “E11.1”, “E11.8”, “E11.9”, “E13.1”, “E13.9”])

WHERE

Diagnosis.StartDate < Surgery.Date

DEFINE condition Diabetes With Chronic Complications AS

(Diagnosis.Code IN "E10.2+”, “E10.3+”, “E10.4+”, “E10.5”, “E11.2+”, “E11.3+”, “E11.4+”, “E11.5”, “E13.2+”, “E13.3+”, “E13.4+”, “E13.5”, “E10.6”, “E11.6”, “E13.6”])

WHERE

Diagnosis.StartDate < Surgery.Date

DEFINE condition Diabetes DCRA AS

(Form.Description = "DCRA"

AND FormResponse.Question = "Diabetes mellitus"

AND FormResponse.Answer = "Yes)

*Dictionary*

| **Dutch** | **English** |
| --- | --- |
| Diabetes / diabetes mellitus | diabetes / diabetes mellitus |
| type 1 | type 1 |
| type 2 / type II | type 2 |
| niet insuline-afhankelijke | non-insulin-dependent |
| zonder complicaties | without complications |
| met oogcomplicaties | with ocular complications |
| met niet gespecificeerde complicaties | with unspecified complications |
| met complicaties van perifere circulatie | with peripheral circulation complications |
| met neurologische complicaties | with neurological complications |
| glomerulaire aandoeningen bij | glomerular disorders in |
| gecompliceerde | complicated |
| met niercomplicaties | with renal complications |
| overige gespecificeerde vormen van | other specified forms of |
| reeds bestaande | pre-existing |
| met ketoacidose | with ketoacidosis |

**Query 11 Renal disease**

DEFINE condition Mild or moderate renal AS

((Diagnosis.Description CONTAINS "dialyse patient"

OR Diagnosis.Description CONTAINS “dialyse patiente”

OR Diagnosis.Description CONTAINS “peritoneaal dialyse”

OR Diagnosis.Description CONTAINS “acute nierinsufficientie met dialyse”

OR Diagnosis.Description CONTAINS “nierinsufficientie waarvoor continue ambulante peritoneale dialyse”

OR Diagnosis.Description CONTAINS “nierinsufficientie waarvoor automatische peritoneale dialyse”

OR Diagnosis.Description CONTAINS “peritoneale dialyse CAPD thuis”

OR Diagnosis.Description CONTAINS “CAPD dialyse”

OR Diagnosis.Description CONTAINS “overige dialyse”

OR Diagnosis.Description CONTAINS “start dialyse”

OR Diagnosis.Description CONTAINS “acute nierinsufficientie met tubulusnecrose met dialyse”

OR Diagnosis.Description CONTAINS “extracorporale dialyse”

OR Diagnosis.Description CONTAINS “niertransplantatie”

OR Diagnosis.Description CONTAINS “niertransplantatie ontvanger”

OR Diagnosis.Description CONTAINS “aanwezigheid van niertransplantaat”

OR Diagnosis.Description CONTAINS “status na niertransplantatie”

OR Diagnosis.Description CONTAINS “afstoting van niertransplantaat”

OR Diagnosis.Description CONTAINS “afstoting na niertransplantatie”

OR Diagnosis.Description CONTAINS “tweede niertransplantatie”

OR Diagnosis.Description CONTAINS “mislukken van niertransplantatie en afstoting”

OR Diagnosis.Description CONTAINS “status na lever-niertransplantatie ontvanger”

OR Diagnosis.Description CONTAINS “pre-transplantatie screening van ontvanger bij niertransplantatie”

OR Diagnosis.Description CONTAINS “nacontrole na niertransplantatie 1 jaar 3 jaar”

OR Diagnosis.Description CONTAINS “acute rejectie van niertransplantaat”

OR Diagnosis.Description CONTAINS “cardiale beoordeling ivm mogelijk niertransplantatie”

OR Diagnosis.Description CONTAINS “chronische rejectie van niertransplantaat”

OR Diagnosis.Description CONTAINS “nacontrole na niertransplantatie 3 jaar”

OR Diagnosis.Description CONTAINS “vertraagde functie van niertransplantaat”

OR

(Diagnosis.Code IN ["I12.9”, “I13.0”, “N03.9”, “N03.8”, “N03.2”, “N03.0”, “N03.4”, “N03.1”, “N05.9”, “N05.1”, “N05.2”, “N05.8”, “N05.0”, “N05.5”, “N05.6”, “N05.4”, “N05.7”, “N18.1”, “N18.2”, “N18.3”, “N18.4”, “N18.9”, “Z94.0”, “I13.1”]))

WHERE

Diagnosis.StartDate < Surgery.Date

DEFINE condition Servere Renal AS

(Diagnosis.Code IN "I12.0”, “N18.5”, “N19”, “N25.0”, “Z49.1”, “Z49.2”, “Z49.0”, “Z99.2”, “I13.1”])

WHERE

Diagnosis.StartDate < Surgery.Date

DEFINE condition Nierziekten DCRA AS

(Form.Description = "DCRA"

AND FormResponse.Question = "Nierziekte"

AND FormResponse.Answer = "Yes)

*Dictionary*

| **Dutch** | **English** |
| --- | --- |
| dialyse patient / patiente | dialysis patient |
| peritoneaal dialyse | peritoneal dialysis |
| acute nierinsufficientie | acute renal insufficiency |
| continue ambulante peritoneale dialyse | continuous ambulatory peritoneal dialysis |
| automatische peritoneale dialyse | automated peritoneal dialysis |
| CAPD thuis / CAPD dialyse | CAPD at home / CAPD dialysis |
| overige dialyse | other dialysis |
| start dialyse | start of dialysis |
| tubulusnecrose | tubular necrosis |
| extracorporale dialyse | extracorporeal dialysis |
| niertransplantatie / ontvanger / afstoting | kidney transplant / recipient / rejection |
| status na niertransplantatie | post-kidney transplant status |
| tweede niertransplantatie | second kidney transplant |
| mislukken van niertransplantatie | failed kidney transplant |
| lever-niertransplantatie | liver-kidney transplant |
| pre-transplantatie screening | pre-transplant screening |
| nacontrole 1-3 jaar | follow-up 1-3 years |
| acute rejectie / chronische rejectie | acute rejection / chronic rejection |
| vertraagde functie | delayed function |

**Query 12 Hemiplegia**

DEFINE condition Hemiplegia AS

((Diagnosis.Description CONTAINS "hemiplegie"

OR Diagnosis.Description CONTAINS “hemiplegie niet gespecificeerd”

OR Diagnosis.Description CONTAINS “infantiele hemiplegie”

OR Diagnosis.Description CONTAINS “spastische hemiplegie”

OR Diagnosis.Description CONTAINS “congenitale hemiplegie”

OR Diagnosis.Description CONTAINS “hypertone hemiplegie”

OR Diagnosis.Description CONTAINS “hypotone hemiplegie”

OR Diagnosis.Description CONTAINS “spastische hemiplegische cerebrale paralyse”

OR Diagnosis.Description CONTAINS “cerebral palsy hemiplegic postnatal infantile”

OR Diagnosis.Description CONTAINS “hemiparese”

OR Diagnosis.Description CONTAINS “hemiparese door cerebrovasculair accident”

OR Diagnosis.Description CONTAINS “linkszijdige hemiparese”

OR Diagnosis.Description CONTAINS “hemiparese als restverschijnsel na cerebrovasculair accident”

OR Diagnosis.Description CONTAINS “hemiparese rechts en afasie”

OR Diagnosis.Description CONTAINS “atactische hemiparase”

OR Diagnosis.Description CONTAINS “alternerende hemiparese”

OR Diagnosis.Description CONTAINS “rechtzijdige hemiparese”

OR Diagnosis.Description CONTAINS “CVA met hemiparese rechts en afasie”

OR

(Diagnosis.Code IN ["G11.4”, “G80.0”, “G80.1”, “G80.2”, “G81.9”, “G81.1”, “G81.0”, “G82.2”, “G82.5”, “G82.1”, “G82.4”, “G82.0”, “G83.4”, “G83.2”, “G83.1”, “G83.3”, “G83.0”, “G83.5”, “G83.9”, “G83.8”]))

WHERE

Diagnosis.StartDate < Surgery.Date

DEFINE condition Hemiplegie DCRA AS

(Form.Description = "DCRA"

AND FormResponse.Question = "Para-/hemiplegie"

AND FormResponse.Answer = "Yes)

*Dictionary*

| **Dutch** | **English** |
| --- | --- |
| hemiplegie | hemiplegia |
| niet gespecificeerd | unspecified |
| infantiele | infantile |
| spastische | spastic |
| congenitale | congenital |
| hypertone | hypertonic |
| hypotone | hypotonic |
| cerebrale paralyse | cerebral palsy |
| postnataal | postnatal |
| hemiparese | hemiparesis |
| door cerebrovasculair accident | due to cerebrovascular accident |
| linkszijdig | left-sided |
| rechtszijdig | right-sided |
| als restverschijnsel | as residual symptom |
| met afasie | with aphasia |
| atactische | ataxic |
| alternerende | alternating |

**Query 13 Any malignancy**

DEFINE condition Any malignancy AS

(Diagnosis.Code IN "C20”, “C25.9”, “C22.9”, “C22.0”, “C21.0”, “C25.2”, “C22.1”, “C25.1”, “C24.0”, “C26.0”, “C24.1”, “C23”, “C26.9”, “C25.3”, “C25.4”, “C22.2”, “C26.1”, “C25.8”, “C25.7”, “C22.3”, “C21.1”, “C21.8”, “C25.0”, “C24.9”, “C50.9”, “C61”, “C62.9”, “C34.9”, “C18.9”, “C18.7”, “C18.2”, “C15.9”, “C34.1”, “C43.9”, “C34.3”, “C18.0”, “C43.5”, “C18.4”, “C19”, “C16.9”, “C09.9”, “C07”, “C01”, “C06.9”, “C02.9”, “C02.1”, “C04.9”, “C08.0”, “C06.0”, “C00.9”, “C05.2”, “C05.0”, “C06.2”, “C08.9”, “C05.9”, “C02.4”, “C00.0”, “C18.6”, “C16.0”, “C17.0”, “C18.3”, “C10.9”, “C15.5”, “C13.9”, “C18.1”, “C11.9”, “C18.5”, “C17.2”, “C17.1”, “C15.8”, “C16.3”, “C16.2”, “C15.2”, “C15.3”, “C17.9”, “C16.4”, “C30.0”, “C31.9”, “C31.3”, “C31.0”, “C31.1”, “C32.0”, “C32.1”, “C32.9”, “C33”, “C34.2”, “C34.0”, “C34.8”, “C37”, “C38.3”, “C38.1”, “C38.2”, “C38.0”, “C38.4”, “C40.2”, “C40.0”, “C40.3”, “C40.9”, “C40.1”, “C41.9”, “C41.2”, “C41.4”, “C41.3”, “C41.0”, “C41.1”, “C43.3”, “C43.6”, “C43.7”, “C43.2”, “C43.0”, “C43.1”, “C45.0”, “C45.9”, “C45.1”, “C45.7”, “C46.9”, “C46.1”, “C46.0”, “C47.2”, “C47.6”, “C47.9”, “C47.4”, “C47.1”, “C48.2”, “C48.0”, “C48.1”, “C49.9”, “C49.2”, “C49.1”, “C49.4”, “C49.3”, “C49.6”, “C49.0”, “C49.5”, “C50.4”, “C50.8”, “C50.2”, “C50.5”, “C50.3”, “C50.1”, “C50.6”, “C50.0”, “C51.9”, “C51.0”, “C51.1”, “C52”, “C53.9”, “C53.0”, “C53.1”, “C53.8”, “C54.1”, “C54.9”, “C54.2”, “C54.8”, “C55”, “C56”, “C57.4”, “C57.9”, “C57.0”, “C58”, “C60.9”, “C60.1”, “C60.2”, “C60.0”, “C62.0”, “C62.1”, “C63.2”, “C63.7”, “C63.0”, “C76.1”, “C76.0”, “C76.2”, “C76.3”, “C76.7”, “C76.4”, “C76.5”, “C80.9”, “C80”, “C81.9”, “C81.0”, “C81.1”, “C81.4”, “C81.2”, “C81.7”, “C82.6”, “C82.0”, “C82.9”, “C82.7”, “C82.2”, “C82.3”, “C82.1”, “C82.4”, “C83.1”, “C83.0”, “C83.7”, “C83.3”, “C83.9”, “C83.8”, “C83.5”, “C84.0”, “C84.1”, “C84.5”, “C84.4”, “C84.7”, “C84.6”, “C84.9”, “C84.8”, “C85.1”, “C85.9”, “C85.7”, “C85.2”, “C88.0”, “C88.4”, “C88.7”, “C88.3”, “C88.9”, “C90.1”, “C90.0”, “C90.3”, “C90.2”, “C94.3”, “C92.0”, “C91.0”, “C91.4”, “C93.1”, “C95.9”, “C96.8”, “C92.5”, “C93.3”, “C91.1”, “C92.1”, “C96.2”, “C96.9”, “C95.0”, “C91.7”, “C97”, “C92.7”, “C92.4”, “C91.3”, “C95.1”, “C91.9”, “C91.5”, “C96.6”, “C92.2”, “C91.6”, “C93.0”])

WHERE

Diagnosis.StartDate < Surgery.Date

DEFINE condition Maligniteit DCRA AS

(Form.Description = "DCRA"

AND FormResponse.Question = "Maligniteit excl. PCC BCC"

AND FormResponse.Answer = "Yes)

DEFINE condition Metastatic Solid Tumor AS

(Diagnosis.Code IN "C77.9”, “C77.1”, “C77.2”, “C77.3”, “C77.0”, “C77.5”, “C77.8”, “C77.4”, “C78.7”, “C78.0”, “C78.6”, “C78.2”, “C78.8”, “C78.5”, “C78.1”, “C78.4”, “C78.3”, “C79.5”, “C79.3”, “C79.8”, “C79.4”, “C79.9”, “C79.7”, “C79.2”, “C79.0”, “C79.1”, “C79.6”, “C80.0”])

WHERE

Diagnosis.StartDate < Surgery.Date

*Dictionary*

| **Dutch** | **English** |
| --- | --- |
| Maligniteit | malignancy |

**Query 14 HIV/AIDS**

DEFINE condition HIV Infection AS

(Diagnosis.Code IN "B20.9”, “B20.2”, “B20.6”, “B20.4”, “B20.1”, “B20.7”, “B20.3”, “B21.0”, “B21.3”, “B21.2”, “B22.2”, “B22.0”, “B22.1”, “B23.1”, “B23.8”, “B23.0”])

WHERE

Diagnosis.StartDate < Surgery.Date

DEFINE condition AIDS AS

(Diagnosis.Code IN "B24”])

WHERE

Diagnosis.StartDate < Surgery.Date

DEFINE condition AIDS DCRA AS

(Form.Description = "DCRA"

AND FormResponse.Question = "HIV AIDS"

AND FormResponse.Answer = "Yes)

*Preoperative medical data*

**Query 1 Frailty**

DEFINE condition Kwetsbaarheid AS

(Form.Description CONTAINS "Clinical Frailty Scale")

WHERE

Form.StartDate < Surgery.Date

*Dictionary*

| **Dutch** | **English** |
| --- | --- |
| Kwetsbaarheid | frailty |

**Query 2 ASA-score**

DEFINE variable ASA_Score AS

((Form.Description = "Gastro-Enterologische chirurgie Operatieverslag"

AND FormResponse.Question CONTAINS "ASA score"

OR FormResponse.Question CONTAINS "ASA score zoals gerapporteerd door de anesthesist"

OR FormResponse.Question CONTAINS "ASA-score"

AND FormResponse.Answer IS NOT NULL)

OR

(Report.ReportType = "Preoperatieve screening"

AND Report.Specialism = "Anesthesiologie"

AND Report.ReportDate < Surgery.Date

AND Report.Content CONTAINS "ASA-score")

**Query 3 MET-score**

DEFINE variable MET_Score AS

((Form.Description = "Preoperatieve screening"

AND FormResponse.Question CONTAINS "MET score”)

OR

(Report.ReportType = "Preoperatieve screening"

AND Report.Content CONTAINS "MET-score"))

*Dictionary*

| **Dutch** | **English** |
| --- | --- |
| ASA score / ASA-score | ASA score |
| zoals gerapporteerd door de anesthesist | as reported by the anesthesiologist |
| MET score / MET-score | MET score |
| Preoperatieve screening | Preoperative screening |
| Anesthesiologie | Anesthesiology |
| Gastro-enterologische chirurgie operatieverslag | Gastroenterological surgery operative report |

**Query 4 Hemoglobin level before start prehabilitation**

DEFINE variable Hb_BeforePrehab AS

(Lab.ResultType = "Hemoglobine"

AND Lab.ResultDate BETWEEN (Prehabilitation.IntakeDate - 30 days) AND Prehabilitation.IntakeDate

AND Lab.ResultValue IS NOT NULL)

*Dictionary*

| **Dutch** | **English** |
| --- | --- |
| Hemoglobine | Hemoglobin level |

**Query 5 Hemoglobin level before surgery**

DEFINE variable Hb_BeforeSurgery AS

(Lab.ResultType = "Hemoglobine"

AND Lab.ResultDate BETWEEN (Surgery.Date - 30 days) AND Surgery.Date

AND Lab.ResultValue IS NOT NULL)

*Dictionary*

| **Dutch** | **English** |
| --- | --- |
| Hemoglobine | Hemoglobin level |

**Query 6 Ferinject (iron infusion)**

DEFINE variable Ferinject_Admin AS

((Medication.MedicationName CONTAINS "Ferinject"

OR Medication.MedicationName CONTAINS "ijzer carboxymaltose"

OR Medication.MedicationName CONTAINS "ferinjectinfuus”

OR Medication.MedicationName CONTAINS "ferinject infusie”

OR Medication.MedicationName CONTAINS "ferinject 18 jaar”

OR Medication.MedicationName CONTAINS "ferinject IV”

OR Medication.MedicationName CONTAINS "ferinject 1000mg”

OR Medication.MedicationName CONTAINS "ferinject 1000 mg”

OR Medication.MedicationName CONTAINS "ferinject + 2 LVs

OR Medication.MedicationName CONTAINS "toedienen Ferinject”)

AND Medication.AdministrationDate < Surgery.Date)

*Dictionary*

| **Dutch** | **English** |
| --- | --- |
| Ferinject | Ferinject |
| ijzer carboxymaltose | iron carboxymaltose |
| infuus / infusie | infusion |
| 18 jaar | ≥18 years |
| IV | intraveneous |
| toedienen | administration |

**Query 7 Blood transfusion**

DEFINE variable Transfusion AS

((Procedure.Description CONTAINS "bloedtransfusie"

OR Medication.MedicationName CONTAINS "erytrocytenconcentraat"

OR Medication.MedicationName CONTAINS "packed cells"

OR Medication.MedicationName CONTAINS "bloedtransfusie 2 LVE 65 jaar + Ferinject”

OR Medication.MedicationName CONTAINS "bloedtransfusie 3 LVE 65 jaar + Ferinject”

OR Medication.MedicationName CONTAINS "bloedtransfusie 2 LVE 65 jaar+ferinject

OR Medication.MedicationName CONTAINS "bloedtransfusie 2l.v 65 + ferinject”

OR Medication.MedicationName CONTAINS "bloedtransfusie 2 LVE 65 jaar+ferinject)

AND Procedure.Date < Surgery.Date)

OR (Measurement.Name CONTAINS “Transfusiegegev.”

AND Lab.ResultDate < (OK datum - 30 days)

AND Lab.ResultValue IS NOT NULL)

*Dictionary*

| **Dutch** | **English** |
| --- | --- |
| Ferinject | Ferinject |
| Bloedtransfusie | Blood transfusion |
| erytrocytenconcentraat | erythrocyte concentrate |
| packed cells | packed cells |
| 2 LVE 65 jaar | 2 units, age ≥65 |
| 3 LVE 65 jaar | 3 units, age ≥65 |
| 2 LVE 65 jaar | 2 units, age ≥65 |
| 2l.v 65+ | 2 units, age ≥65 |
| Transfusiegegev. | Transfusion data |

**Query 8 Date first POS appointment**

DEFINE variable POS_Appointment1_Date AS

(Appointment.Specialism = "Anesthesiologie"

AND Appointment.Description CONTAINS "POS"

OR Appointment.Code IN [“FP40”, “FP”, “TC P”]

AND Appointment.Status = "Voldaan"

AND Appointment.StartDate < (Surgery.Date – 6 months))

*Dictionary*

| **Dutch** | **English** |
| --- | --- |
| Anesthesiologie | Anesthesiology |
| POS | Preoperative screening |
| Voldaan | Completed |

**Query 9 Body length at first POS appointment**

DEFINE variable Height_POS AS

((Form.Description = "Preoperatieve screening"

AND FormResponse.Question CONTAINS "Lengte"

AND FormResponse.Answer IS NOT NULL)

OR

(VitalSigns.MeasurementType = "Lengte"

AND VitalSigns.MeasurementDate BETWEEN (POS_Appointment1_Date – 3 months) AND (POS_Appointment1_Date + 1 day)))

*Dictionary*

| **Dutch** | **English** |
| --- | --- |
| Preoperatieve screening | Preoperative screening |
| Lengte | Body length |

**Query 10 Body weight at first POS appointment**

DEFINE variable Weight_POS AS

((Form.Description = "Preoperatieve screening"

AND FormResponse.Question CONTAINS "Gewicht"

AND FormResponse.Answer IS NOT NULL)

OR

(VitalSigns.MeasurementType = "Gewicht"

AND VitalSigns.MeasurementDate BETWEEN (POS_Appointment1_Date – 3 months) AND (POS_Appointment1_Date + 1 day)))

*Dictionary*

| **Dutch** | **English** |
| --- | --- |
| Preoperatieve screening | Preoperative screening |
| Gewicht | Body weigth |

*Intoxications*

**Query 1 Alcohol use**

Define variable Alcohol AS

((Form.Description = “Alcohol”

AND FormResponse.Question CONTAINS “Alcohol”

AND FormReponse.Answer IS NOT NULL) BETWEEN

AND FormResponse.StartDate < (Surgery.Date – 2 months))

**Query 2 Amount of alcohol**

Define variable Hoeveelheid alcohol AS

((Form.Description = “Alcohol”

AND FormReponse.Question CONTAINS “Hoeveelheid alcohol”

AND FormResponse.Question CONTAINS “Opmerkingen alcohol”

AND FormResponse.Answer IS NOT NULL) BETWEEN

AND FormResponse.StartDate < (Surgery.Date – 2 months))

*Dictionary*

| **Dutch** | **English** |
| --- | --- |
| Hoeveelheid alcohol | Amount of alcohol |
| Opmerkingen alcohol | Alcohol remarks |

**Query 3 Smoking**

Define variable Roken AS

((Form.Description = “Roken”

AND FormResponse.Question CONTAINS “Roken”

AND FormReponse.Answer IS NOT NULL) BETWEEN

AND FormResponse.StartDate < (Surgery.Date – 2 months))

*Dictionary*

| **Dutch** | **English** |
| --- | --- |
| Roken | Smoking |

**Query 4 Smoking packyears**

Define variable Packyears AS

((Form.Description = “Roken”

AND FormResponse.Question CONTAINS “Aantal packyears”

AND FormReponse.Answer IS NOT NULL) BETWEEN

AND FormResponse.StartDate < (Surgery.Date – 2 months))

*Dictionary*

| **Dutch** | **English** |
| --- | --- |
| Roken | Smoking |
| Aantal packyears | Amount of packyears |

*Surgical data*

**Query 1 Date of first appointment surgeon**

DEFINE variable Datum afspraak chirurg 1 AS

(Appointment.Code IN ["NPSZCORE", "NPSZCOLO", "NPSZCOL0", "NP GEO", "NP GEO20"]

AND Appointment.StartDate BETWEEN '2018-01-01' AND '2023-09-01'

AND Appointment.Status IN ["Voldaan”])

*Dictionary*

| **Dutch** | **English** |
| --- | --- |
| Datum afspraak chirurg | Date surgical appointment |
| Voldaan | Completed |

**Query 2 Number of tumors**

DEFINE variable Aantal tumoren AS

(Form.Description = “Gastro-Enterologische Chirurgie Operatieverslag”

AND FormResponse.Question CONTAINS “Aantal gediagnosticeerde carcinomen”)

*Dictionary*

| **Dutch** | **English** |
| --- | --- |
| Aantal tumoren | Number of tumors |
| Aantal gediagnosticeerde carcinomen | Number of diagnosed carcinomas |
| Gastro-Enterologische Chirurgie Operatieverslag | Gastro-enterological surgery operation report |

**Query 3 Surgery**

DEFINE variable Operatie AS

(Form.Description = “Gastro-Enterologische Chirurgie Operatieverslag”

AND FormResponse.Question CONTAINS “1e chirurgische procedure”

AND FormReponse.TextualAnswer CONTAINS ANY OF [“(extended) hemicolectomie rechts”, “ileocoecaal resectie”, “transversectomie”, “(extended) hemicolectomie links”, “partiele mesorectal excisie (PME)”, “totaal Mesorectale Excisie (TME)”, “subtotale colectomie (coecum tot rectum)”, “abdomino-perineale resectie (APR)”, “proctolectomie (coecum tot en met rectum)”, “lokale excisie colon”, “ sigmoid resectie”, “transanale lokale resectie”, “overig”, “resectie gelijk aan procedure 1^e^ tumor”)

*Dictonairy*

| **Dutch** | **English** |
| --- | --- |
| Operatie | Surgery |
| 1e chirurgische procedure | First surgical procedure |
| (extended) hemicolectomie rechts | (extended) right hemicolectomy |
| ileocoecaal resectie | ileocecal resection |
| transversectomie | transversectomy |
| (extended) hemicolectomie links | (extended) left hemicolectomy |
| partiele mesorectal excisie (PME) | partial mesorectal excision (PME) |
| totaal Mesorectale Excisie (TME) | total mesorectal excision (TME) |
| subtotale colectomie (coecum tot rectum) | subtotal colectomy (cecum to rectum) |
| abdomino-perineale resectie (APR) | abdominoperineal resection (APR) |
| proctolectomie (coecum tot en met rectum) | proctocolectomy (cecum to rectum) |
| lokale excisie colon | local excision colon |
| sigmoid resectie | sigmoid resection |
| transanale lokale resectie | transanal local resection |
| overig | other |
| resectie gelijk aan procedure 1e tumor | resection same as first tumor procedure |
| Gastro-Enterologische Chirurgie Operatieverslag | Gastro-enterological surgery operation report |

**Query 4 Date of surgery**

DEFINE variable OK datum AS

(SurgeryProcedures.Name IN [“034738”, “034739”, “034733”, “034735”, “034732”, “034734”, “034736”, “035024”, “035025”, “035023”, “035027”, “035026”, “334704C”, “334720C”, “334720”, “334704”, “334720G”, “334710B”, “334710”, “334704A”, “334704D”, “334702A”, “334720E”, “334702E”, “334730A”, “334703A”, “334703D”, “334703F”, “334703B”, “334704B”, “334702B”, “334702D”, “334702”, “334702C”, “334711D”, “334710A”, “334711F”, “334711”, “334711A”, “334711C”, “334711E”, “334720H”, “334720B”, “334720A”, “334720F”, “334810:”, “334756”, “334730C”, “334731A”, “334824”, “334730E”, “334752L”, “334752R”, “334823”, “334828E”, “334822R”, “334828D”, “334821”, “334827”, “334828”, “334822L”, “334828B”, “334811”, “334814B”, “334759E”, “334755”, “335025C”, “335024C”, “335025B”, “335015”, “335024A”, “335025F”, “335024”, “335025N”, “335025E”, “335025J”, “335024B”, “335024D”, “335023”, “335026A”, “335022”, “335025I”, “334759D”, “334649”, “334742”, “334761E”, “334742A”, “334750A”, “334793”, “334759B”, “334759A”, “334757D”, “334752”].

*Dictonairy*

| **Dutch** | **English** |
| --- | --- |
| OK datum | Surgery date |

**Query 5 Length of hospital stay**

DEFINE variable Opnameduur AS

Admission.InitialSpecialism = "Chirurgie gastro-enterologie / oncologie"

AND Admission.StartDate BETWEEN (Surgery.Date - 7 days) AND Surgery.Date

AND Admission.EndDate >= Surgery.Date)

*Dictonairy*

| **Dutch** | **English** |
| --- | --- |
| Opnameduur | Length of hospital stay |
| Chirurgie gastro-enterologie / oncologie | Gastro-enterological surgery / oncology |

**Query 6 Level of urgency surgery**

DEFINE variable Urgentie operatie AS

(Form.Description = “Gastro-Enterologische Chirurgie Operatieverslag”

AND FormResponse.Question CONTAINS “Urgentie operatie”)

*Dictionary*

| **Dutch** | **English** |
| --- | --- |
| Urgentie operatie | Surgery urgency |
| Gastro-Enterologische Chirurgie Operatieverslag | Gastro-enterological surgery operation report |

**Query 6 Surgery procedure**

DEFINE variable Procedure AS

(Form.Description = “Gastro-Enterologische Chirurgie Operatieverslag”

AND FormResponse.Question CONTAINS “Benadering 1e chirurgische procedure”)

*Dictionary*

| **Dutch** | **English** |
| --- | --- |
| Urgentie operatie | Surgery urgency |
| gastro-enterologische chirurgie operatieverslag | Gastro-enterological surgery operation report |

**Query 6 Converted surgery**

DEFINE variable Conversie AS

(Form.Description = “Gastro-Enterologische Chirurgie Operatieverslag”

AND FormResponse.Question CONTAINS “Heeft er een conversie plaatsgevonden”)

*Dictionary*

| **Dutch** | **English** |
| --- | --- |
| gastro-enterologische chirurgie operatieverslag | gastroenterological surgery operative report |
| conversie | conversion |

**Query 7 TNM pathology**

DEFINE variable TNM pathologie AS

(Report.ReportType = "Pathologieverslag"

AND Report.ReportDate > Surgery.Date

AND Report.Content CONTAINS ANY OF "TNM classificatie Colon en Rectum")

*Dictionary*

| **Dutch** | **English** |
| --- | --- |
| pathologieverslag | pathology report |
| TNM classificatie Colon en Rectum | TNM classification colon and rectum |

**Query 8 Neoadjuvant therapies**

DEFINE variable Neoadjuvante therapie AS

(Report.ReportType = "Pathologieverslag"

AND Report.ReportDate > Surgery.Date

AND Report.Content CONTAINS ANY OF "Neo-adjuvante Therapie")

*Dictionary*

| **Dutch** | **English** |
| --- | --- |
| pathologieverslag | pathology report |
| Neo-adjuvante therapie | Neoadjuvant therapy |

**Query 9 Tumor location**

DEFINE variable Tumorlocatie AS

(Form.Description = “Gastro-Enterologische Chirurgie Operatieverslag”

AND FormResponse.Question CONTAINS “Lokalisatie 1e colorectaal carcinoom”)

OR FormResponse.Question CONTAINS “Lokalisatie 2e colorectaal carcinoom”)

OR FormResponse.Question CONTAINS “Waaraan wordt de patient nu geopereerd”)

*Dictionary*

| **Dutch** | **English** |
| --- | --- |
| gastro-enterologische chirurgie operatieverslag | gastroenterological surgery operative report |
| Lokalisatie 1e colorectaal carcinoom | location first colorectal carcinoma |
| Lokalisatie 2e colorectaal carcinoom | location second colorectal carcinoma |
| Waaraan wordt de patient nu geopereerd | current surgical site |

*Postoperative complications*

**Query 1 Complications in total**

DEFINE variable Complicaties totaal AS

(Form.Description = “DCRA”

AND FormResponse.Question CONTAINS “Zijn er complicaties opgetreden binnen 90 dagen na de resectie”)

*Dictionary*

| **Dutch** | **English** |
| --- | --- |
| Complicaties totaal | Total complications |
| Zijn er complicaties opgetreden binnen 90 dagen na de resectie | any complications within 90 days post-resection |

**Query 2 Surgical complications**

DEFINE variable Chirurgische complicaties AS

(Form.Description = “DCRA”

AND FormResponse.Question CONTAINS “Is er een chirurgische complicatie opgetreden”)

*Dictionary*

| **Dutch** | **English** |
| --- | --- |
| Chirurgische complicatie | Surgical complication |
| Is er een chirurgische complicatie opgetreden | Any surgical complication |

**Query 3 Pulmonary complication**

DEFINE variable Pulmonale complicatie AS

(Form.Description = “DCRA”

AND FormResponse.Question CONTAINS “Pulmonale complicatie”)

*Dictionary*

| **Dutch** | **English** |
| --- | --- |
| Pulmonale complicatie | Pulmonary complication |

**Query 4 Cardiac complication**

DEFINE variable Cardiale complicatie AS

(Form.Description = “DCRA”

AND FormResponse.Question CONTAINS “Cardiale complicatie”)

*Dictionary*

| **Dutch** | **English** |
| --- | --- |
| Cardiale complicatie | Cardiac complication |

**Query 5 Tromboembolic complication**

DEFINE variable Tromboembolische complicatie AS

(Form.Description = “DCRA”

AND FormResponse.Question CONTAINS “Tromboembolische complicatie”)

*Dictionary*

| **Dutch** | **English** |
| --- | --- |
| Tromboembolische complicatie | Tromboembolic complication |

**Query 6 Infectious complication**

DEFINE variable Infectieuze complicatie AS

(Form.Description = “DCRA”

AND FormResponse.Question CONTAINS “Infectieuze complciatie anders dan pulmonale of chirurgische infectie”)

*Dictionary*

| **Dutch** | **English** |
| --- | --- |
| Infectieuze complicatie | Infectious complication |

**Query 7 Neurologic complication**

DEFINE variable Neurologische complicatie AS

(Form.Description = “DCRA”

AND FormResponse.Question CONTAINS “Neurologische complicatie”)

*Dictionary*

| **Dutch** | **English** |
| --- | --- |
| Neurologische complicatie | Neurologic complication |

**Query 8 Other complication**

DEFINE variable Andere complicatie AS

(Form.Description = “DCRA”

AND FormResponse.Question CONTAINS “Andere complicatie”)

*Dictionary*

| **Dutch** | **English** |
| --- | --- |
| Andere complicatie | Other complication |

**Query 9 Reintervention**

DEFINE variable Re-interventie AS

(Form.Description = “DCRA”

AND FormResponse.Question CONTAINS “Heeft hiervoor een re-interventie plaatsgevonden”)

*Dictionary*

| **Dutch** | **English** |
| --- | --- |
| Re-interventie | Re-intervention |
| Heeft hiervoor een re-interventie plaatsgevonden | Was a re-intervention performed |

**Query 10 Blood transfusion**

DEFINE variable Bloedtransfusie AS

(Form.Description = “DCRA”

AND FormResponse.Question CONTAINS “Bloedtransfusie tijdens opname”)

*Dictionary*

| **Dutch** | **English** |
| --- | --- |
| Bloedtransfusie | Bloodtransfusion |
| Bloedtransfusie tijdens opname | Bloodtransfusion during hospital stay |

**Query 11 Readmission**

DEFINE variable Heropname AS

(Form.Description = “DCRA”

AND FormResponse.Question CONTAINS “Is de patient 90 dagen na ontslag heropgenomen”)

*Dictionary*

| **Dutch** | **English** |
| --- | --- |
| Heropname | Re-admission |
| Is de patiënt 90 dagen na ontslag heropgenomen | Was the patient readmitted within 90 days after discharge |

*Dietitian intake data*

*Dictionary*

**Query 1 Time of date first appointment**

DEFINE variable Datum afspraak 1 AS

(Appointment.Specialism = “Dietetiek”

AND Appointment.Description CONTAINS “NP PREHA”

AND Appointment.Status IN [“Voldaan”, “Onbekend”])

*Dictionary*

| **Dutch** | **English** |
| --- | --- |
| Datum afspraak 1 | date of first appointment |
| Dietetiek | Dietitian |
| Voldaan | completed |
| Onbekend | unknown |

**Query 2 Body length at intake dietitian**

DEFINE variable Lengte 1 AS

(Form.Description = “Meet- en weeggegevens”

AND FormRespons.Question CONTAINS “Lengte cm”

OR FormRespons.Question CONTAINS “Meetmethode voor lengte”

AND Form.StartDate = DietetiekAfspraak1.StartDate)

*Dictionary*

| **Dutch** | **English** |
| --- | --- |
| Meet- en weeggegevens | measurement and weighing data |
| Lengte 1 | body length at intake |
| Lengte cm | length cm |
| Meetmethode voor lengte | measurement method for length |

**Query 3 Body weight at intake dietitian**

DEFINE variable Gewicht 1 AS

(Form.Description = “Meet- en weeggegevens”

AND FormRespons.Question CONTAINS “Gewicht”

AND Form.StartDate = DietetiekAfspraak1.StartDate)

*Dictionary*

| **Dutch** | **English** |
| --- | --- |
| Meet- en weeggegevens | measurement and weighing data |
| Gewicht 1 | body weight at intake |

**Query 4 BMI at intake dietitian**

DEFINE variable BMI 1 AS

(Form.Description = “Meet- en weeggegevens”

AND FormRespons.Question CONTAINS “BMI”

AND Form.StartDate = DietetiekAfspraak1.StartDate)

*Dictionary*

| **Dutch** | **English** |
| --- | --- |
| Meet- en weeggegevens | measurement and weighing data |

**Query 5 Percentage weight loss at intake dietitian**

DEFINE variable Gewichtsverlies AS

(Form.Description = “Meet- en weeggegevens”

AND FormRespons.Question CONTAINS “Toelichting gewichtsverandering”

OR FormRespons.Question CONTAINS “Gewichtsverandering %”

AND Form.StartDate = DietetiekAfspraak1.StartDate)

*Dictionary*

| **Dutch** | **English** |
| --- | --- |
| Meet- en weeggegevens | measurement and weighing data |
| Gewichtsverlies | percentage weight loss at intake |
| Toelichting gewichtsverandering | explanation of weight change |
| Gewichtsverandering % | weight change % |

**Query 6 Physical limitations handgrip strength at intake dietitian**

DEFINE variable Beperkingen handknijpkracht 1 AS

((Form.Description = “Meet- en weeggegevens”

AND FormRespons.Question CONTAINS “Geeft de patient eventuele beperkingen aan de hand aan”

AND Form.StartDate = DietetiekAfspraak1.StartDate)

OR (Form.Description = “Handknijpkrachtmeting”

AND FormRespons.Question CONTAINS “Geeft de patient eventuele beperkingen aan de hand aan”

AND Form.StartDate = DietetiekAfspraak1.StartDate))

*Dictionary*

| **Dutch** | **English** |
| --- | --- |
| Meet- en weeggegevens | measurement and weighing data |
| Handknijpkrachtmeting | handgrip strength measurement |
| Beperkingen handknijpkracht 1 | handgrip limitations at intake |
| Geeft de patient eventuele beperkingen aan de hand aan | patient indicates any hand limitations |

**Query 7 Left handgrip strength at intake dietitian**

DEFINE variable Handknijpkracht L1 AS

((Report.ReportType = "Consult”, “Polikliniek: vervolgconsult”, “Kliniek: vervolgconsult”, “Polikliniek: eerste consult”, “Telefonisch consult”, “Kliniek: eerste consult””

AND Report.CareProvider IN ["diëtist", "diëtetiek", "Diëtist", “Dietist JBZ”, “Dietist assistente”, ‘Inval Dietist”]

AND Report.Content CONTAINS "Maximale handknijpkracht"

AND Report.ReportDate = DietetiekAfspraak1.StartDate)

OR

(Form.Description = "Meet- en weeggegevens"

AND (FormResponse.Question CONTAINS "Maximale handknijpkracht is")

AND Form.StartDate = DietetiekAfspraak1.StartDate)

OR

(Form.Description CONTAINS "Handknijpkrachtmeting, meting linkerhand"

AND (FormResponse.Question CONTAINS "Maximale handknijpkracht is")

AND Form.StartDate = DietetiekAfspraak1.StartDate)

OR

(Form.Description CONTAINS "meting linkerhand"

AND (FormResponse.Question CONTAINS "maximale handknijpkracht is"))

*Dictionary*

| **Dutch** | **English** |
| --- | --- |
| Meet- en weeggegevens | measurement and weighing data |
| Handknijpkrachtmeting, meting linkerhand | handgrip measurement, left hand |
| meting linkerhand | left hand measurement |
| Handknijpkracht L1 | Left handgrip strength at intake |
| Maximale handknijpkracht | maximal handgrip strength |
| Consult | consultation |
| Polikliniek: vervolgconsult | outpatient: follow-up consultation |
| Kliniek: vervolgconsult | clinic: follow-up consultation |
| Polikliniek: eerste consult | outpatient: first consultation |
| Telefonisch consult | telephone consultation |
| Kliniek: eerste consult | clinic: first consultation |
| diëtist / diëtetiek / Dietist JBZ / Dietist assistente / Inval Dietist | dietitian / dietetics / Dietitian JBZ / Dietitian assistant / Locum dietitian |

**Query 8 Right handgrip strength at intake dietitian**

DEFINE variable Handknijpkracht R1 AS

((Report.ReportType = "Consult”, “Polikliniek: vervolgconsult”, “Kliniek: vervolgconsult”, “Polikliniek: eerste consult”, “Telefonisch consult”, “Kliniek: eerste consult””

AND Report.CareProvider IN ["diëtist", "diëtetiek", "Diëtist", “Dietist JBZ”, “Dietist assistente”, ‘Inval Dietist”]

AND Report.Content CONTAINS "Maximale handknijpkracht"

AND Report.ReportDate = DietetiekAfspraak1.StartDate)

OR

(Form.Description = "Meet- en weeggegevens"

AND (FormResponse.Question CONTAINS "Maximale handknijpkracht is")

AND Form.StartDate = DietetiekAfspraak1.StartDate)

OR

(Form.Description CONTAINS "Handknijpkrachtmeting, meting rechterhand"

AND (FormResponse.Question CONTAINS "Maximale handknijpkracht is")

AND Form.StartDate = DietetiekAfspraak1.StartDate)

OR

(Form.Description CONTAINS "meting rechterhand"

AND (FormResponse.Question CONTAINS "maximale handknijpkracht is"))

*Dictionary*

| **Dutch** | **English** |
| --- | --- |
| Meet- en weeggegevens | measurement and weighing data |
| Handknijpkrachtmeting, meting rechterhand | handgrip measurement, right hand |
| meting rechterhand | right hand measurement |
| Handknijpkracht R1 | right handgrip strength at intake |
| Maximale handknijpkracht | maximal handgrip strength |
| Consult | consultation |
| Polikliniek: vervolgconsult | outpatient: follow-up consultation |
| Kliniek: vervolgconsult | clinic: follow-up consultation |
| Polikliniek: eerste consult | outpatient: first consultation |
| Telefonisch consult | telephone consultation |
| Kliniek: eerste consult | clinic: first consultation |
| diëtist / diëtetiek / Dietist JBZ / Dietist assistente / Inval Dietist | dietitian / dietetics / Dietitian JBZ / Dietitian assistant / Locum dietitian |

**Query 9 Fatfree mass at intake dietitian**

DEFINE variable Vetvrije massa 1 AS

(Form.Description = “Meet- en weeggegevens”

AND FormRespons.Question CONTAINS “VVM vetvrije massa”

AND Form.StartDate = DietetiekAfspraak1.StartDate)

*Dictionary*

| **Dutch** | **English** |
| --- | --- |
| Meet- en weeggegevens | measurement and weighing data |
| VVM vetvrije massa | Fat Free Mass |

**Query 10 Fatfree mass index at intake dietitian**

DEFINE variable Vetvrije massa index 1 AS

(Form.Description = “Meet- en weeggegevens”

AND FormRespons.Question CONTAINS “VVM vetvrije massa index”

AND Form.StartDate = DietetiekAfspraak1.StartDate)

*Dictionary*

| **Dutch** | **English** |
| --- | --- |
| Meet- en weeggegevens | measurement and weighing data |
| VVM vetvrije massa index | Fat Free Mass index |

**Query 11 Appetite at intake dietitian**

DEFINE variable Eetlust 1 AS

((Report.ReportType = "Consult”, “Polikliniek: vervolgconsult”, “Kliniek: vervolgconsult”, “Polikliniek: eerste consult”, “Telefonisch consult”, “Kliniek: eerste consult””

AND Report.CareProvider IN ["diëtist", "diëtetiek", "Diëtist", “Dietist JBZ”, “Dietist assistente”, ‘Inval Dietist”]

AND Report.Content CONTAINS "Verminderde eetlust"

OR Report.Content CONTAINS “Eetlust VAS-score”

AND Report.ReportDate = DietetiekAfspraak1.StartDate)

OR

(Form.Description = "Dietistisch onderzoek & dietistische diagnose"

AND (FormResponse.Question CONTAINS "Eetlust VAS score")

AND Form.StartDate = DietetiekAfspraak1.StartDate))

*Dictionary*

| **Dutch** | **English** |
| --- | --- |
| Consult | consultation |
| Polikliniek: vervolgconsult | outpatient: follow-up consultation |
| Kliniek: vervolgconsult | clinic: follow-up consultation |
| Polikliniek: eerste consult | outpatient: first consultation |
| Telefonisch consult | telephone consultation |
| Kliniek: eerste consult | clinic: first consultation |
| diëtist / diëtetiek / Diëtist JBZ / Dietist assistente / Inval Dietist | dietitian / dietetics / Dietitian JBZ / Dietitian assistant / Locum dietitian |
| Verminderde eetlust | reduced appetite |
| Eetlust VAS-score | appetite VAS score |
| Dietistisch onderzoek & dietistische diagnose | dietetic assessment & dietetic diagnosis |
| Eetlust 1 | appetite at intake |

**Query 12 Alcohol use at intake dietitian**

DEFINE variable Alcoholgebruik 1 AS

(Report.ReportType = "Consult”, “Polikliniek: vervolgconsult”, “Kliniek: vervolgconsult”, “Polikliniek: eerste consult”, “Telefonisch consult”, “Kliniek: eerste consult””

AND Report.CareProvider IN ["diëtist", "diëtetiek", "Diëtist", “Dietist JBZ”, “Dietist assistente”, ‘Inval Dietist”]

AND Report.Content CONTAINS "Alcoholgebruik"

AND Report.ReportDate = DietetiekAfspraak1.StartDate)

*Dictionary*

| **Dutch** | **English** |
| --- | --- |
| Consult | consultation |
| Polikliniek: vervolgconsult | outpatient: follow-up consultation |
| Kliniek: vervolgconsult | clinic: follow-up consultation |
| Polikliniek: eerste consult | outpatient: first consultation |
| Telefonisch consult | telephone consultation |
| Kliniek: eerste consult | clinic: first consultation |
| diëtist / diëtetiek / Diëtist JBZ / Dietist assistente / Inval Dietist | dietitian / dietetics / Dietitian JBZ / Dietitian assistant / Locum dietitian |
| Alcoholgebruik 1 | alcohol use at intake |

**Query 13 Smoking at intake dietitian**

DEFINE variable Roken 1 AS

(Report.ReportType = "Consult”, “Polikliniek: vervolgconsult”, “Kliniek: vervolgconsult”, “Polikliniek: eerste consult”, “Telefonisch consult”, “Kliniek: eerste consult””

AND Report.CareProvider IN ["diëtist", "diëtetiek", "Diëtist", “Dietist JBZ”, “Dietist assistente”, ‘Inval Dietist”]

AND Report.Content CONTAINS "Roken"

AND Report.ReportDate = DietetiekAfspraak1.StartDate)

*Dictionary*

| **Dutch** | **English** |
| --- | --- |
| Consult | consultation |
| Polikliniek: vervolgconsult | outpatient: follow-up consultation |
| Kliniek: vervolgconsult | clinic: follow-up consultation |
| Polikliniek: eerste consult | outpatient: first consultation |
| Telefonisch consult | telephone consultation |
| Kliniek: eerste consult | clinic: first consultation |
| diëtist / diëtetiek / Diëtist JBZ / Dietist assistente / Inval Dietist | dietitian / dietetics / Dietitian JBZ / Dietitian assistant / Locum dietitian |
| Roken | smoking |

**Query 14 Suppletion at intake dietitian**

DEFINE variable Suppletie vit/min 1 AS

(Report.ReportType = "Consult”, “Polikliniek: vervolgconsult”, “Kliniek: vervolgconsult”, “Polikliniek: eerste consult”, “Telefonisch consult”, “Kliniek: eerste consult””

AND Report.Content CONTAINS "Vitaminen-/mineralensuppletie"

AND Report.ReportDate = DietetiekAfspraak1.StartDate)

*Dictionary*

| **Dutch** | **English** |
| --- | --- |
| Consult | Consult |
| vervolgconsult | follow-up consult |
| eerste consult | first consult |
| Kliniek | Clinic |
| Telefonisch | Telephone |
| Vitaminen-/mineralensuppletie | vitamin/mineral supplementation |

**Query 15 Percentage of energy intake at intake dietitian**

DEFINE variable % Energie-inname 1 AS

((Report.ReportType = "Consult”, “Polikliniek: vervolgconsult”, “Kliniek: vervolgconsult”, “Polikliniek: eerste consult”, “Telefonisch consult”, “Kliniek: eerste consult””

AND Report.CareProvider IN ["diëtist", "diëtetiek", "Diëtist", “Dietist JBZ”, “Dietist assistente”, ‘Inval Dietist”]

AND Report.Content CONTAINS "% van energiebehoefte (kcal/dag)"

AND Report.ReportDate = DietetiekAfspraak1.StartDate)

OR

(Form.Description = "Voedingsintake & behoefte

AND (FormResponse.Question CONTAINS "% van Energiebehoefte kcal dag")

AND Form.StartDate = DietetiekAfspraak1.StartDate))

*Dictionary*

| **Dutch** | **English** |
| --- | --- |
| Consult | Consult |
| vervolgconsult | follow-up consult |
| eerste consult | first consult |
| Kliniek | Clinic |
| Telefonisch | Telephone |
| Voedingsintake & behoefte | Nutritional intake & requirements |
| % van energiebehoefte (kcal/dag) | % of energy requirement (kcal/day) |

**Query 16 Percentage of protein intake at intake dietitian**

DEFINE variable % Eiwit-inname 1 AS

((Report.ReportType = "Consult”, “Polikliniek: vervolgconsult”, “Kliniek: vervolgconsult”, “Polikliniek: eerste consult”, “Telefonisch consult”, “Kliniek: eerste consult””

AND Report.CareProvider IN ["diëtist", "diëtetiek", "Diëtist", “Dietist JBZ”, “Dietist assistente”, ‘Inval Dietist”]

AND Report.Content CONTAINS "% van eiwitbehoefte (kcal/dag)"

AND Report.ReportDate = DietetiekAfspraak1.StartDate)

OR

(Form.Description = "Voedingsintake & behoefte

AND (FormResponse.Question CONTAINS "% van Energiebehoefte g dag")

AND Form.StartDate = DietetiekAfspraak1.StartDate))

*Dictionary*

| **Dutch** | **English** |
| --- | --- |
| Consult | Consult |
| vervolgconsult | follow-up consult |
| eerste consult | first consult |
| Kliniek | Clinic |
| Telefonisch | Telephone |
| Voedingsintake & behoefte | Nutritional intake & requirements |
| % van eiwitbehoefte g dag | % of protein requirement (g/day) |

*Dietitian outtake data*

**Query 1 Time of date last appointment**

DEFINE variable Datum afspraak 2 AS

(Appointment.Specialism = “Dietetiek”

AND Appointment.Description CONTAINS “CP PREHA”

AND Appointment.Status IN [“Voldaan”, “Onbekend”]

AND DietetiekAfspraak2.StartDate < Surgery.Date)

*Dictionary*

| **Dutch** | **English** |
| --- | --- |
| Datum afspraak 2 | date of last appointment |
| Dietetiek | dietitian |
| Voldaan | completed |
| Onbekend | unknown |

**Query 2 Body weight at outtake dietitian**

DEFINE variable Gewicht 2 AS

(Form.Description = “Meet- en weeggegevens”

AND FormRespons.Question CONTAINS “Gewicht”

AND Form.StartDate = DietetiekAfspraak2.StartDate)

*Dictionary*

| **Dutch** | **English** |
| --- | --- |
| Meet- en weeggegevens | measurement and weighing data |
| Gewicht 2 | body weight at outtake |

**Query 3 Physical limitations handgrip strength at outtake dietitian**

DEFINE variable Beperkingen handknijpkracht 2 AS

((Form.Description = “Meet- en weeggegevens”

AND FormRespons.Question CONTAINS “Geeft de patient eventuele beperkingen aan de hand aan”

AND Form.StartDate = DietetiekAfspraak2.StartDate)

OR (Form.Description = “Handknijpkrachtmeting”

AND FormRespons.Question CONTAINS “Geeft de patient eventuele beperkingen aan de hand aan”

AND Form.StartDate = DietetiekAfspraak2.StartDate))

*Dictionary*

| **Dutch** | **English** |
| --- | --- |
| Meet- en weeggegevens | measurement and weighing data |
| Handknijpkrachtmeting | handgrip strength measurement |
| Beperkingen handknijpkracht 2 | handgrip limitations at outtake |
| Geeft de patient eventuele beperkingen aan de hand aan | patient indicates any hand limitations |

**Query 4 Left handgrip strength at outtake dietitian**

DEFINE variable Handknijpkracht L2 AS

((Form.Description = "Meet- en weeggegevens"

AND (FormResponse.Question CONTAINS "Maximale handknijpkracht is")

AND Form.StartDate = DietetiekAfspraak2.StartDate)

OR

(Form.Description CONTAINS "Handknijpkrachtmeting, meting linkerhand"

AND (FormResponse.Question CONTAINS "Maximale handknijpkracht is")

AND Form.StartDate = DietetiekAfspraak2.StartDate)

OR

(Form.Description CONTAINS "meting linkerhand"

AND (FormResponse.Question CONTAINS "maximale handknijpkracht is"))

*Dictionary*

| **Dutch** | **English** |
| --- | --- |
| Meet- en weeggegevens | measurement and weighing data |
| Handknijpkrachtmeting, meting linkerhand | handgrip measurement, left hand |
| meting linkerhand | left hand measurement |
| Handknijpkracht L2 | Left handgrip strength at outtake |
| Maximale handknijpkracht | maximal handgrip strength |

**Query 5 Right handgrip strength at outtake dietitian**

DEFINE variable Handknijpkracht R2 AS

((Form.Description = "Meet- en weeggegevens"

AND (FormResponse.Question CONTAINS "Maximale handknijpkracht is")

AND Form.StartDate = DietetiekAfspraak2.StartDate)

OR

(Form.Description CONTAINS "Handknijpkrachtmeting, meting rechterhand"

AND (FormResponse.Question CONTAINS "Maximale handknijpkracht is")

AND Form.StartDate = DietetiekAfspraak2.StartDate)

OR

(Form.Description CONTAINS "meting rechterhand"

AND (FormResponse.Question CONTAINS "maximale handknijpkracht is"))

*Dictionary*

| **Dutch** | **English** |
| --- | --- |
| Meet- en weeggegevens | measurement and weighing data |
| Handknijpkrachtmeting, meting rechterhand | handgrip measurement, right hand |
| meting rechterhand | right hand measurement |
| Handknijpkracht R2 | right handgrip strength at outtake |
| Maximale handknijpkracht | maximal handgrip strength |

**Query 6 Fatfree mass at outtake dietitian**

DEFINE variable Vetvrije massa 2 AS

(Form.Description = “Meet- en weeggegevens”

AND FormRespons.Question CONTAINS “VVM vetvrije massa”

AND Form.StartDate = DietetiekAfspraak2.StartDate)

*Dictionary*

| **Dutch** | **English** |
| --- | --- |
| Meet- en weeggegevens | measurement and weighing data |
| VVM vetvrije massa | Fat Free Mass |

**Query 7 Fatfree mass index at outtake dietitian**

DEFINE variable Vetvrije massa index 2 AS

(Form.Description = “Meet- en weeggegevens”

AND FormResponse.Question CONTAINS “VVM vetvrije massa index”

AND Form.StartDate = DietetiekAfspraak2.StartDate)

*Dictionary*

| **Dutch** | **English** |
| --- | --- |
| Meet- en weeggegevens | measurement and weighing data |
| VVM vetvrije massa index | Fat Free Mass index |

**Query 8 Alcohol use at outtake dietitian**

DEFINE variable Alcoholgebruik 2 AS

(Report.ReportType = "Consult”, “Polikliniek: vervolgconsult”, “Kliniek: vervolgconsult”, “Polikliniek: eerste consult”, “Telefonisch consult”, “Kliniek: eerste consult””

AND Report.CareProvider IN ["diëtist", "diëtetiek", "Diëtist", “Dietist JBZ”, “Dietist assistente”, ‘Inval Dietist”]

AND Report.Content CONTAINS "Alcoholgebruik"

AND Report.ReportDate = DietetiekAfspraak2.StartDate)

*Dictionary*

| **Dutch** | **English** |
| --- | --- |
| Consult | consultation |
| Polikliniek: vervolgconsult | outpatient: follow-up consultation |
| Kliniek: vervolgconsult | clinic: follow-up consultation |
| Polikliniek: eerste consult | outpatient: first consultation |
| Telefonisch consult | telephone consultation |
| Kliniek: eerste consult | clinic: first consultation |
| diëtist / diëtetiek / Diëtist JBZ / Dietist assistente / Inval Dietist | dietitian / dietetics / Dietitian JBZ / Dietitian assistant / Locum dietitian |
| Alcoholgebruik 2 | alcohol use at outtake |

**Query 9 Smoking at outtake dietitian**

DEFINE variable Roken 2 AS

(Report.ReportType = "Consult”, “Polikliniek: vervolgconsult”, “Kliniek: vervolgconsult”, “Polikliniek: eerste consult”, “Telefonisch consult”, “Kliniek: eerste consult””

AND Report.CareProvider IN ["diëtist", "diëtetiek", "Diëtist", “Dietist JBZ”, “Dietist assistente”, ‘Inval Dietist”]

AND Report.Content CONTAINS "Roken"

AND Report.ReportDate = DietetiekAfspraak2.StartDate)

OR (Form.Description = “Roken”

AND FormResponse.Question CONTAINS “Roken”

AND Report.ReportDate = DietetiekAfspraak2.StartDate))

*Dictionary*

| **Dutch** | **English** |
| --- | --- |
| Consult | consultation |
| Polikliniek: vervolgconsult | outpatient: follow-up consultation |
| Kliniek: vervolgconsult | clinic: follow-up consultation |
| Polikliniek: eerste consult | outpatient: first consultation |
| Telefonisch consult | telephone consultation |
| Kliniek: eerste consult | clinic: first consultation |
| diëtist / diëtetiek / Diëtist JBZ / Dietist assistente / Inval Dietist | dietitian / dietetics / Dietitian JBZ / Dietitian assistant / Locum dietitian |
| Roken | smoking |

**Query 10 Suppletion at outtake dietitian**

DEFINE variable Suppletie vit/min 2 AS

(Report.ReportType = "Consult”, “Polikliniek: vervolgconsult”, “Kliniek: vervolgconsult”, “Polikliniek: eerste consult”, “Telefonisch consult”, “Kliniek: eerste consult””

AND Report.Content CONTAINS "Vitaminen-/mineralensuppletie"

AND Report.ReportDate = DietetiekAfspraak2.StartDate)

*Dictionary*

| **Dutch** | **English** |
| --- | --- |
| Consult | Consult |
| vervolgconsult | follow-up consult |
| eerste consult | first consult |
| Kliniek | Clinic |
| Telefonisch | Telephone |
| Vitaminen-/mineralensuppletie | vitamin/mineral supplementation |

**Query 11 Percentage of protein intake at outtake dietitian**

DEFINE variable % Eiwit-inname 2 AS

((Report.ReportType = "Consult”, “Polikliniek: vervolgconsult”, “Kliniek: vervolgconsult”, “Polikliniek: eerste consult”, “Telefonisch consult”, “Kliniek: eerste consult””

AND Report.CareProvider IN ["diëtist", "diëtetiek", "Diëtist", “Dietist JBZ”, “Dietist assistente”, ‘Inval Dietist”]

AND Report.Content CONTAINS “Inname tijdens het prehabilitatie traject”

AND Report.ReportDate = DietetiekAfspraak2.StartDate)

OR

(Form.Description = "Voedingsintake & behoefte

AND (FormResponse.Question CONTAINS "Voedingsadvies met betrekking tot eiwitinname")

AND Form.StartDate = DietetiekAfspraak2.StartDate))

*Dictionary*

| **Dutch** | **English** |
| --- | --- |
| Consult | Consult |
| vervolgconsult | follow-up consult |
| eerste consult | first consult |
| Kliniek | Clinic |
| Telefonisch | Telephone |
| Voedingsintake & behoefte | Nutritional intake & requirements |
| % van eiwitbehoefte g dag | % of protein requirement (g/day) |

*Physiotherapist data*

**Query 1 Number of training sessions**

DEFINE variable Aantal trainingen AS

((Appointment.Specialism = "Fysiotherapie"

AND (Appointment.Description CONTAINS "Pre Habilitatie"

OR Appointment.Description CONTAINS "Intake Pre Hab")

AND Appointment.StartDate BETWEEN (Surgery.Date - 3 months) AND (Surgery.Date - 1 day))

*Dictionary*

| **Dutch** | **English** |
| --- | --- |
| Fysiotherapie | Physiotherapy |
| Pre Habilitatie | Prehabilitation |
| Intake Pre hab | Prehabilitation intake |

**Query 2 6-minute walk test**

DEFINE variable 6MWT AS

(Appointment.Specialism = “Fysiotherapie”

AND Report.ReportType = “Polikliniek: vervolgconsult”

AND Report.Content CONTAINS "6MWT")

*Dictionary*

| **Dutch** | **English** |
| --- | --- |
| Fysiotherapie | Physiotherapy |
| Polikliniek: vervolgconsult | Outpatient: follow-up consult |

**Query 3 Steep Ramp Test**

DEFINE variable SRT AS

(Appointment.Specialism = “Fysiotherapie”

AND Report.ReportType = “Polikliniek: vervolgconsult”

AND Report.Content CONTAINS "steepramptest")

*Dictionary*

| **Dutch** | **English** |
| --- | --- |
| Fysiotherapie | Physiotherapy |
| Polikliniek: vervolgconsult | Outpatient: follow-up consult |
