# Supplementary material for: Predicting which colorectal cancer patients are most likely to improve their functional capacity with pre-surgery prehabilitation: a retrospective study based on the 6-min walk distance
Source: Support Care Cancer. 2026 Jul 27;34(8):805. doi: 10.1007/s00520-026-11039-5 (PMC13407944; doi:10.1007/s00520-026-11039-5)
Supplement: Supplementary file 3 — (DOCX 26.5 KB) [file 520_2026_11039_MOESM3_ESM.docx]

Predicting which colorectal cancer patients are most likely to improve their functional capacity with pre-surgery prehabilitation: A retrospective study based on the six-minute walk distance. Supportive Care in Cancer. M. de Klerk, M.J.W. van der Linden, A.P.M. Kerckhoffs, B.R. Meijboom, E.G.G. Verdaasdonk, E. de Vries. Tranzo Scientific Centre for Care and Wellbeing, Tilburg School of Social and Behavioral Sciences, Tilburg University, Warandelaan 2 5037 AB Tilburg, The Netherlands, m.deklerk@tilburguniversity.edu

**Supplementary Information 3. Overview of prehabilitation studies including the 6-minute walk distance in colorectal surgery**

This supplementary table summarizes key studies evaluating multimodal prehabilitation in colorectal cancer (CRC) surgery that included the 6-minute walk distance (6MWD) as a measure of functional capacity.

**Supplementary Table S1. Summary key studies evaluating 6MWD**

| Study | Population & Design | 6MWD Assessment | Outcomes | Key Findings |
| --- | --- | --- | --- | --- |
| Molenaar et al., 2023[1] | RCT, CRC patients (prehabilitation versus usual care) | Baseline and 4 weeks postoperative (change in 6MWD, >20m improvement) | Postoperative complications, 6MWD change, quality of life | Prehabilitation group maintained or improved 6MWD; controls declined. More patients achieved >20 m improvement. |
| Gillis et al., 2022[2] | Pooled analysis of 5 trials | Pre-op 6MWD (% predicted and <400m) | Functional improvement by nutritional status | Prehabilitation benefits greatest in moderately malnourished patients (PG-SGA 4–8). |
| Barrett-Bernstein et al., 2019[3] | Secondary analysis of 2 prospective studies | Baseline, 4 and 8 weeks postoperative (<400m and >20m improvement) | Functional performance and capacity, psychological symptoms | Prehabilitation improved 6MWD, especially in patients with depressive symptoms. |
| Carli et al., 2010[4] | RCT, CRC patients (prehabilitation versus usual care) | Prehabilitation period and postoperative follow-up (% predicted, >20m improvement) | Functional capacity | No significant between-group difference in 6MWD; supervised protocols may yield better gains. |
| Gillis et al., 2014[5] | RCT, CRC patients | Baseline, preoperative, 4 and 8 weeks postoperative 6MWD (% predicted, <400m, >20m improvement) | 6MWD and recovery trajectory | Prehabilitation improved 6MWD by +25 m vs decline of −16 m in controls; mean difference 41.7 m. |
| Li et al., 2013[6] | Pilot RCT, CRC patients | Baseline, preoperative, 4 and 8 weeks postoperative | Functional recovery | Prehabilitation improved 6MWD by +40 m preoperatively; 6MWD <392 m predicted complications. |

**References**

1. Molenaar CJL, Minnella EM, Coca-Martinez M, Ten Cate DWG, Regis M, Awasthi R, et al. Effect of Multimodal Prehabilitation on Reducing Postoperative Complications and Enhancing Functional Capacity Following Colorectal Cancer Surgery. JAMA Surgery. 2023;158(6):572. https://doi.org/ 10.1001/jamasurg.2023.1553.

2. Gillis C, Fenton TR, Gramlich L, Keller H, Sajobi TT, Culos-Reed SN, et al. Malnutrition modifies the response to multimodal prehabilitation: a pooled analysis of prehabilitation trials. Applied physiology, nutrition, and metabolism. 2022;47(2):141–50. https://doi.org/10.1139/APNM-2021-0299.

3. Barrett-Bernstein M, Carli F, Gamsa A, Scheede-Bergdahl C, Minnella E, Ramanakumar AV, et al. Depression and Functional Status in Colorectal Cancer Patients Awaiting Surgery: Impact of a Multimodal Prehabilitation Program. Health Psychology. 2019; 38(10): 900-909. doi: 10.1037/hea0000781.

4. Carli F, Charlebois P, Stein B, Feldman L, Zavorsky G, Kim DJ, et al. Randomized clinical trial of prehabilitation in colorectal surgery. Br J Surg. 2010; 97(8): 1187-97. https://doi.org/ 10.1002/bjs.7102.

5. Gillis C, Li C, Lee L, Awasthi R, Augustin B, Gamsa A, et al. Prehabilitation versus rehabilitation: a randomized control trial in patients undergoing colorectal resection for cancer. Anesthesiology. 2014;121(5):937–47. https://doi.org/10.1097/ALN.0000000000000393.

6. Li C, Carli F, Lee L, Charlebois P, Stein B, Liberman AS, et al. Impact of a trimodal prehabilitation program on functional recovery after colorectal cancer surgery: a pilot study. Surgical endoscopy. 2013;27(4):1072–82. https://doi.org/10.1007/s00464-012-2560-5.
