# Supplementary material for: Predicting which colorectal cancer patients are most likely to improve their functional capacity with pre-surgery prehabilitation: a retrospective study based on the 6-min walk distance
Source: Support Care Cancer. 2026 Jul 27;34(8):805. doi: 10.1007/s00520-026-11039-5 (PMC13407944; doi:10.1007/s00520-026-11039-5)
Supplement: Supplementary file 4 — (DOCX 24.5 KB) [file 520_2026_11039_MOESM4_ESM.docx]

Predicting which colorectal cancer patients are most likely to improve their functional capacity with pre-surgery prehabilitation: A retrospective study based on the six-minute walk distance. Supportive Care in Cancer. M. de Klerk, M.J.W. van der Linden, A.P.M. Kerckhoffs, B.R. Meijboom, E.G.G. Verdaasdonk, E. de Vries. Tranzo Scientific Centre for Care and Wellbeing, Tilburg School of Social and Behavioral Sciences, Tilburg University, Warandelaan 2 5037 AB Tilburg, The Netherlands, m.deklerk@tilburguniversity.edu

**Supplementary Information 4. Methods: Statistical Analysis**Statistical analyses were conducted in R version 4.3 using the lme4, tidyverse, tidymodels, sjPlot, performance, rpart, xgboost, ranger, baguette, and ModelMetrics packages.

In the first statistical analysis (analysis A, see below), models with the meters walked as longitudinal numerical outcome variable (whether measured before or after prehabilitation) were built using linear mixed effects regression (lmer function) with the patient as the grouping variable. All comparisons between models were based on the Akaike Information Criterion (AIC) using ANOVA (analysis of variance), with the lowest AIC indicating the best model.

As a first step in analysis A (step A1), a null-model A (i.e., meterswalked ~ 6MWDtimepoint + (1|pseudoid)) and an all-variables-model A were created (i.e., all independent variables were added as fixed effects to the null-model). Independent variables were selected based on previous literature demonstrating their association with functional capacity or response to prehabilitation, combined with the domain expertise in the CRC team and their (inherent) availability before initiation of the prehabilitation program. We included comorbidities (measured with Charlson Comorbidity Index), CFS, age, sex, ASA score, hemoglobin level, intoxications (alcohol and drugs use), tumor location, number of tumors, neoadjuvant therapies, location of prehabilitation program, and GLIM-defined malnutrition including BMI and weight loss[1-4]. Although adherence to the exercise program is likely to influence improvement in 6MWD, it cannot be incorporated into a prediction model intended for pre-prehabilitation decision-making because attendance is (inherently) unknown at baseline. Definitions of these independent variables are shown in Table 1 in the main article.

Next, each independent variable was tested separately as fixed effect added to the null-model; each of these models was compared to the null-model using ANOVA, and the independent variables whose models performed better than the null-model (based on the lowest AIC) were saved for use in the third step of analysis A.

As a second step in this analysis (step A2), the independent variables described in step A1 were all added to the null-model A as fixed effects (all-variables-model A) and thereafter removed one-by-one with replacement (all-variables-model A minus variable 1, all-variables-model A minus variable 2, etc.). All these models were compared using ANOVA, and independent variables where the model with them had a lower AIC than without them were saved for use in the third step of analysis A.

As a third step in the analysis (step A3), the remaining independent variables from steps A1 and A2 with more than a mild correlation were tested by comparing three models: the one variable added as fixed effect to the null-model, the other variable added as fixed effect to the null-model, and as a pair of variables added as fixed effect to the null-model. Only when the model containing the pair of variables showed the lowest AIC, both variables were retained, otherwise the variable with the lowest AIC in the model with one variable as fixed effect was retained, and the other variable was no longer used in analysis A (for each tested pair).

As a fourth step in the analysis (step A4), the independent variables with more than a mild correlation derived from step A3 were added as pairs to the null-model A in combinations with or without two-way interaction. The two-way interaction was only retained in analysis A when the model with interaction had a lower AIC than the model without.

As a fifth step in the analysis (step A5), the step A2 method was repeated with the remaining variable(s) and two-way interaction(s). Only for variable(s) and interaction(s) where the AIC of the model with was lower than the model without were retained and combined in the final-model A. Final-model performance was visualized using the check_model function and the marginal R^2^ (variance explained by the fixed effects), conditional R^2^ (variance explained by both fixed and random effects), and the root mean square error (RMSE; evaluates the average prediction error).

In the second statistical analysis (analysis B), linear regression (lm function) was used to build the best model for the numerical outcome variable ‘absolute improvement in 6MWD’ (after vs. before prehabilitation), and thereafter – following the same steps – for the numerical outcome variable ‘relative improvement in 6MWD’.

As a first step in this analysis (step B1), all independent variables were tested separately as predictor of the outcome (lm(absolute improvement in 6MWD~ [independent variable]); variables with a p-value being ≥0.05 were no longer used in analysis B for that outcome.

As a second step in this analysis (step B2), the independent variables that remained from step B1 were all used together (all-model B) and thereafter removed one-by-one with replacement (all-model B minus variable 1, all-model B minus variable 2, etc.). All these models were compared using ANOVA, and independent variables where the model without them significantly had lower residuals than the all-model B were no longer used in analysis B. The remaining independent variables were checked for interaction effects as in step A4.

As a third step in this analysis (step B3), the remaining independent variables and interactions from step B2 were combined in the final-model B which was compared with the all-model B using ANOVA; the final-model B was retained unless the all-model B had significantly lower residuals.

In the third statistical analysis (analysis C), logistic regression (glm function) was used to build the best model for the categorical outcome variables: at least 14 meters increase (outtake minus intake value) reached, at least 20 meters increase (outtake minus intake value) reached, at least 400 meters (outtake value) reached, normative value for age (outtake value) reached, and normative value for sex (outtake value) reached. For analysis C, the same recipe and naming of steps and models as described for analysis B was followed consecutively for each categorical outcome variable, with the exception that both the p-value (<0.05) and the AIC (lowest) were used for selection, and the coefficient of discrimination (Tjur’s R^2^; 0 = no discrimination, 1 = perfect discrimination) was used for showing the variance explained.

**References**

1. Perez T, Deslée G, Burgel PR, Caillaud D, Le Rouzic O, Zysman M, et al. Predictors in routine practice of 6-min walking distance and oxygen desaturation in patients with COPD: impact of comorbidities. International Journal of Chronic Obstructive Pulmonary Disease. 2019;14:1399–410. https://doi.org/10.2147/COPD.S188412.

2. Bautmans I, Lambert M, Mets T. The six-minute walk test in community dwelling elderly: influence of health status. BMC Geriatrics. 2004;4(1): 6. https://doi.org/ 10.1186/1471-2318-4-6.

3. Sesso J, Walston J, Bandeen-Roche K, Wu C, Bertoni A, Shah S, et al. Association of Cardiovascular Fibrosis, Remodeling, and Dysfunction With Frailty, Prefrailty, and Functional Performance: The Multi-Ethnic Study of Atherosclerosis. The journals of gerontology Series A, Biological sciences and medical sciences. 2024;79(8). https://doi.org/ 10.1093/gerona/glae142.

4. Gillis C, Fenton TR, Gramlich L, Keller H, Sajobi TT, Culos-Reed SN, et al. Malnutrition modifies the response to multimodal prehabilitation: a pooled analysis of prehabilitation trials. Applied physiology, nutrition, and metabolism. 2022;47(2):141–50. https://doi.org/10.1139/APNM-2021-0299.
