# Supplementary material for: Predicting which colorectal cancer patients are most likely to improve their functional capacity with pre-surgery prehabilitation: a retrospective study based on the 6-min walk distance
Source: Support Care Cancer. 2026 Jul 27;34(8):805. doi: 10.1007/s00520-026-11039-5 (PMC13407944; doi:10.1007/s00520-026-11039-5)
Supplement: Supplementary file 5 — (DOCX 435 KB) [file 520_2026_11039_MOESM5_ESM.docx]

Predicting which colorectal cancer patients are most likely to improve their functional capacity with pre-surgery prehabilitation: A retrospective study based on the six-minute walk distance. Supportive Care in Cancer. M. de Klerk, M.J.W. van der Linden, A.P.M. Kerckhoffs, B.R. Meijboom, E.G.G. Verdaasdonk, E. de Vries. Tranzo Scientific Centre for Care and Wellbeing, Tilburg School of Social and Behavioral Sciences, Tilburg University, Warandelaan 2 5037 AB Tilburg, The Netherlands, m.deklerk@tilburguniversity.edu

**Supplementary Information 5. Results***Statistical analysis A: Linear Mixed Effects Regression (formulas see Methods in the main article and Supplementary Information 4)*

Model A focused on the meters walked using the 6MWD (whether intake or outtake data). In step A1, we constructed the null-model A and the all-variables-model A by respectively adding no and all independent variables as fixed effects to the null model, see Methods. When added individually as fixed effects to the null model, the following variables did not result in a lower AIC compared to the null model and were no longer used in analysis A: smoking, number of tumors, neoadjuvant therapy, and number of training sessions. The remaining variables were entered as fixed effects into the null model (all-variables-model-A) and sequential one-by-one removal with replacement showed that including the variable GLIM did not result in a lower AIC (using ANOVA); it was no longer used in analysis A. In step A3, models with variable pairs with more than a mild correlation were compared with their corresponding single-variable models, resulting in the exclusion of the variable ASA score. Two-way interaction effects between remaining pairs were tested in step A4. Only the two-way interaction between the variables CFS and age had a lower AIC than the model without and was included in further analysis. In Step A5, the remaining variables and two-way interaction were again excluded one-by-one with replacement; the variables alcohol consumption, tumor location, location of prehabilitation program, BMI, and hemoglobin level were excluded because of an equal or lower AIC without them. The final model therefore was: meterswalked ~ 6MWDtimepoint + sex + (CFS * age) + Charlson comorbidity index + weight loss + (1|pseudoid)). Visual demonstration using the check_model function showed a reasonably good fit (Figure 2A). The final model explained 58% of the fixed effects (marginal R^2^) and 94% of the combined fixed and random effects (conditional R^2^); the root mean squared error (RMSE) was 18.8 meters. The relationship between age, meters walked at intake, CFS and achieved 400 meters walked at outtake is shown in Figure 3A, the relationship between meters walked at intake and outtake, age and CFS is shown in Figure 3B.


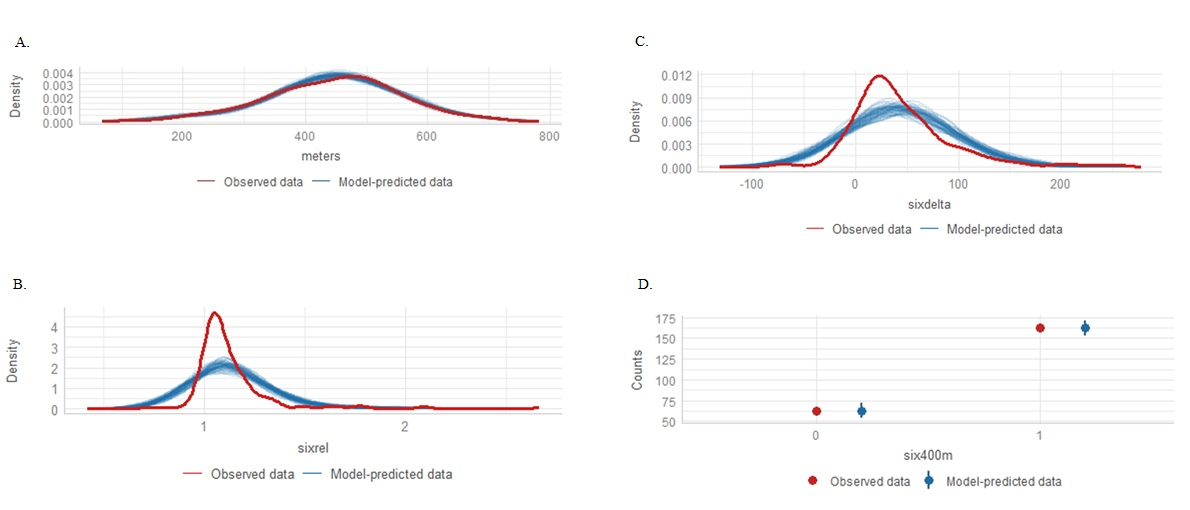


Figure 2 Posterior predictive check of the model A fit, B. absolute improvement in 6MWD (variable name ‘sixdelta’ in figure) fit, C. relative improvement in 6MWD (variable name ‘sixrel’ in figure) fit, D. 6MWD > 400 meters at outtake (variable name ‘six400m’ in figure) fit. For model A, B, and C, good fit model is indicated when the model-predicted lines closely resemble the observed data line, which is observed for model A. For model D, good model fit is indicated when the model predicted intervals include the observed data points without overlap, which is also the case in this panel.


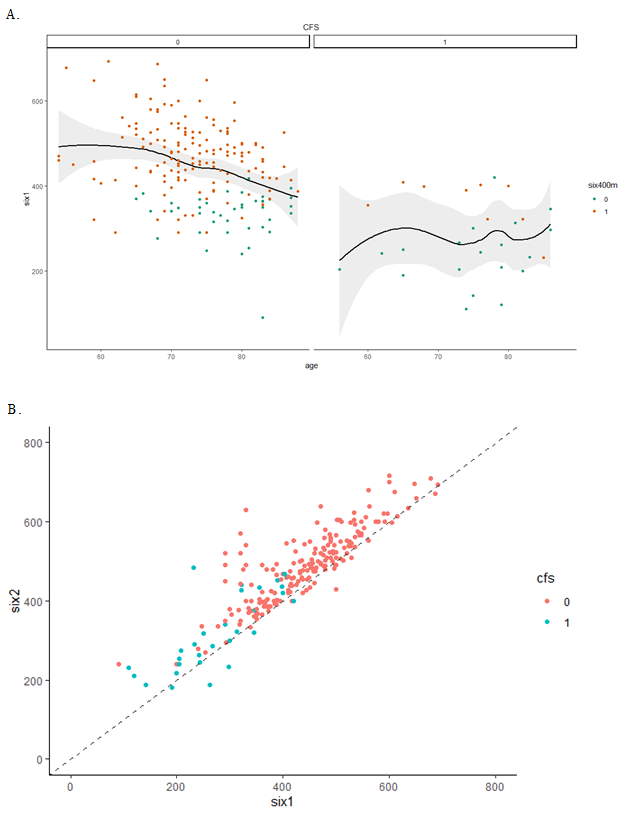


Figure 3A. The relationship between age, meters walked at baseline (six1 in figure), CFS (0 = non-frail on the left, 1 = frail on the right), and 6MWD > 400 meters at outtake(six400 in figure, 0 = false, 1 = true), as modelled using the loss function. B. The relationship between meters walked at baseline (six1 in figure) and meters walked at outtake (six2 in figure), and CFS (0 = non-frail, 1 = frail). The figure includes the 45 degree (dashed) line “x equals y”. Points above this line indicate better performance at outtake than at intake.

*Analysis B: Linear Regression Models*Model B-absolute focused on the absolute increase in meters walked (outtake minus intake) during the 6MWD. Step B1 involved univariate screening, during which variables without significant association (p ≥ .05) were no longer used in analysis B-absolute, being age, CFS, ASA score, hemoglobin level, tumor location, neoadjuvant therapy, number of training sessions, Charlson comorbidity index, BMI, and GLIM. In Step B2, the all-model included the remaining independent variables from step B1. Sequential removal of variables with replacement and model comparison using ANOVA showed that only models with the variables baseline 6MWD, location of prehabilitation program, and weight loss showed significantly lower residuals than without them; no significant interaction effects were found. The remaining independent variables were combined in the final-model B absolute improvement in 6MWD. However, in Step B3 the final-model [lm(absolute improvement in 6MWD ~ baseline 6MWD+location of prehabilitation program+weight loss)] did not differ significantly from the all-model. Visual demonstration using the check_model function showed poor fit of the final-model as shown in Figure 2B.

Model B-relative focused on the relative increase in meters walked during the 6MWD. The same steps as for model B-absolute were followed, and the same final-model was found albeit that here including a three-way interaction between the remaining independent variables showed significantly lower residuals [lm(relative improvement in 6MWD ~ baseline 6MWD*location of prehabilitation program*weight loss)]. However, visual demonstration using the check_model function showed poor fit of the final-model as shown in Figure 2C.

*Analysis C: Logistic Regression Models*Models C focused on the categorical outcome variables (see Methods) and used the same steps as in analysis B. Model C-14m (≥ 14-meter increase in 6MWD) and model C-20m (≥ 20-meter increase in 6MWD) did not show a good fit (Tjur’s R^2^ 0.064 and 0.060 respectively). Model C-pred (achieved predicted normative 6MWD at outtake) did show a reasonable fit (Tjur’s R^2^ = 0.471), although the model predicted data overlapped substantially between patients who achieved the predicted normative 6MWD and those who did not (for the posterior predictive check visual see Supplementary Information 5, Figure S1). Model C-400m (6MWD > 400 meters at outtake) did show a good fit of the final model (glm(6MWD > 400 meters at outtake ~ baseline 6MWD+age)), see Figure 2D (posterior predictive check), with a Tjur’s R^2^ of 0.592.


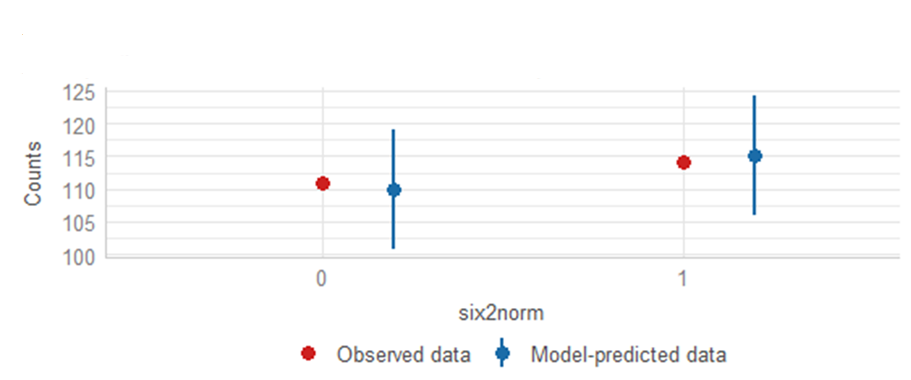


Supplementary Figure S1 Posterior predictive check of the model C-pred (achieved predicted normative 6MWD at outtake [variable name ‘six2norm’ in figure]). Good model fit is indicated when the model predicted intervals include the observed data points without overlap.

*Machine learning*The machine learning models provided a comparative exploration of predictive performance using logistic regression, decision tree, bagged tree, boosted tree, random forest, and support vector machines algorithms, respectively, both all independent variables included in analyses A, B, and C in one set of models (except for boosted tree which does not accept categorical variables), as well as only the independent variables being part of the final-models A, B, and C using the same outcome variable (14m, 20m, 400m, see above) in another set of models in combination with the various outcomes as described. For the outcomes of achieving at least 14 meters or 20 meters improvement at outtake none of the tested algorithms produced a suitable predictive model (MCCs <0.2; ROC-AUCs 0.4-0.6). In contrast, for predicting whether patients reached 400 meters on the 6MWD at outtake, reasonably good model fit could be obtained when using only the independent variables being part of the final models A, B, and C with all the used algorithms except support vector machines which consistently only predicted positives. Upon visual inspection, logistic regression and boosted tree algorithms showed the best fit, the latter also showed the best MCC (but is computationally more intensive, and less straightforward to explain). See Supplementary Table S2 and Supplementary Figure S2A-E.

Supplementary Table S2 A. Confusion matrix using logistic regression for > 400 meters 6MWD at outtake, B. decision tree, C. bagged tree, D. boosted tree, E. random forest.

| *A. Logistic regression* | | |
| --- | --- | --- |
|  | **Truth = 0** | **Truth = 1** |
| **Prediction = 0** | 14 true negative | 6 false negative |
| **Prediction = 1** | 2 false positive | 35 true positive |
| *B. Decision tree* | | |
|  | **Truth = 0** | **Truth = 1** |
| **Prediction = 0** | 14 true negative | 5 false negative |
| **Prediction = 1** | 2 false positive | 36 true positive |
| *C. Bagged tree* | | |
|  | **Truth = 0** | **Truth = 1** |
| **Prediction = 0** | 15 true negative | 7 false negative |
| **Prediction = 1** | 1 false positive | 34 true positive |
| *D. Boosted tree* | | |
|  | **Truth = 0** | **Truth = 1** |
| **Prediction = 0** | 12 true negative | 2 false negative |
| **Prediction = 1** | 4 false positive | 39 true positive |
| *E. Random forest* | | |
|  | **Truth = 0** | **Truth = 1** |
| **Prediction = 0** | 15 true negative | 5 false negative |
| **Prediction = 1** | 1 false positive | 36 true positive |


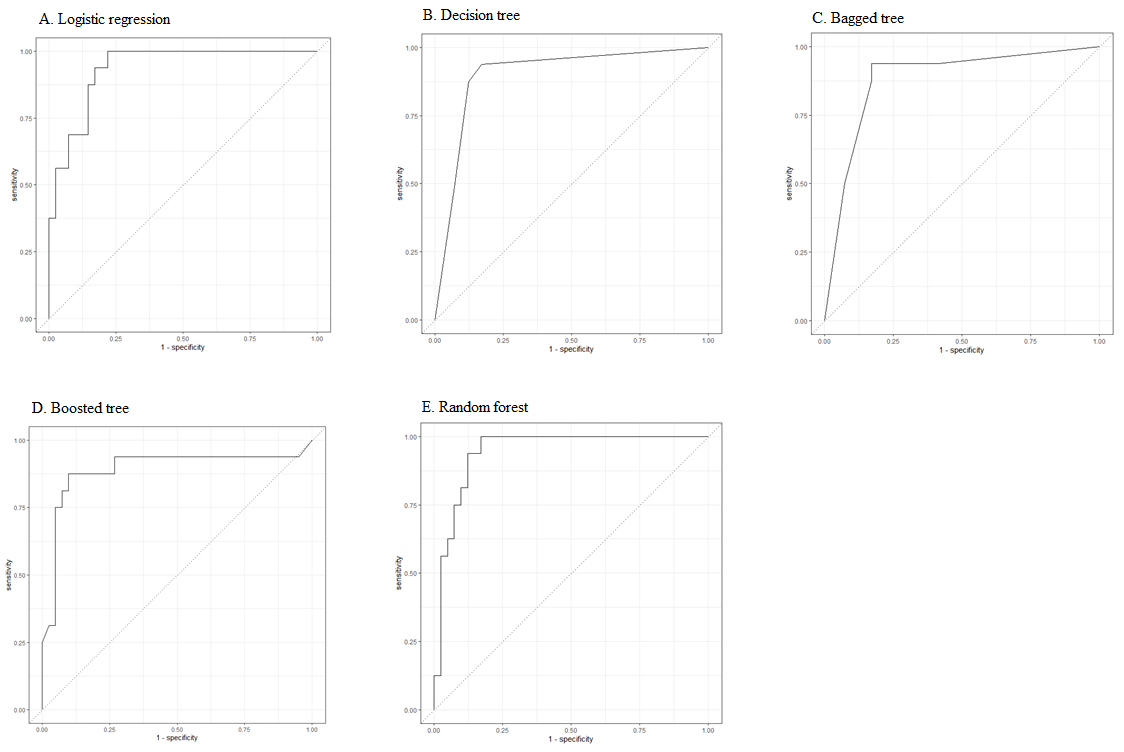


Supplementary Figure S2 A. Predictive model using logistic regression for > 400 meters 6MWD at outtake, B. decision tree, C. bagged tree, D. boosted tree, E. random forest.
